# GSK3 inhibition reduces ECM production and prevents age-related macular degeneration–like pathology

**DOI:** 10.1172/jci.insight.178050

**Published:** 2024-08-08

**Authors:** Sophia M. DiCesare, Antonio J. Ortega, Gracen E. Collier, Steffi Daniel, Krista N. Thompson, Melissa K. McCoy, Bruce A. Posner, John D. Hulleman

**Affiliations:** 1 Department of Ophthalmology, University of Texas Southwestern Medical Center, Dallas, Texas, USA.; 2 Department of Ophthalmology and Visual Neurosciences, University of Minnesota, Minneapolis, Minnesota, USA.; 3 Department of Biochemistry, University of Texas Southwestern Medical Center, Dallas, Texas, USA.

**Keywords:** Ophthalmology, Drug screens, Extracellular matrix, Retinopathy

## Abstract

Malattia Leventinese/Doyne honeycomb retinal dystrophy (ML/DHRD) is an age-related macular degeneration–like (AMD-like) retinal dystrophy caused by an autosomal dominant R345W mutation in the secreted glycoprotein, fibulin-3 (F3). To identify new small molecules that reduce F3 production in retinal pigmented epithelium (RPE) cells, we knocked-in a luminescent peptide tag (HiBiT) into the endogenous F3 locus that enabled simple, sensitive, and high-throughput detection of the protein. The GSK3 inhibitor, CHIR99021 (CHIR), significantly reduced F3 burden (expression, secretion, and intracellular levels) in immortalized RPE and non-RPE cells. Low-level, long-term CHIR treatment promoted remodeling of the RPE extracellular matrix, reducing sub-RPE deposit-associated proteins (e.g., amelotin, complement component 3, collagen IV, and fibronectin), while increasing RPE differentiation factors (e.g., tyrosinase, and pigment epithelium-derived factor). In vivo, treatment of 8-month-old R345W^+/+^ knockin mice with CHIR (25 mg/kg i.p., 1 mo) was well tolerated and significantly reduced R345W F3-associated AMD-like basal laminar deposit number and size, thereby preventing the main pathological feature in these mice. This is an important demonstration of small molecule–based prevention of AMD-like pathology in ML/DHRD mice and may herald a rejuvenation of interest in GSK3 inhibition for the treatment of retinal degenerative diseases, including potentially AMD itself.

## Abstract 2

Administration of a small-molecule enzyme inhibitor can prevent retinal pathology associated with macular degeneration in a mouse model.

## Graphical abstract

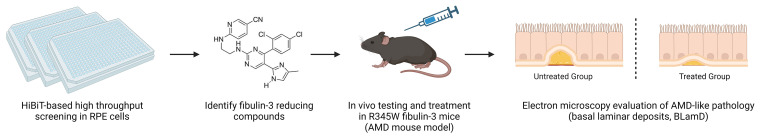

## Introduction

Fibulin-3 (F3) is a secreted extracellular matrix (ECM) glycoprotein that is a member of the core matrisome. F3 is produced in a variety of ocular tissues, including the corneal epithelium, trabecular meshwork ring, optic nerve, and neural retina/retinal pigment epithelium (RPE) (1, 2). Upon secretion from the RPE, F3 is incorporated as a primary component of the RPE basal lamina meshwork, which acts as a sieve between the RPE and the underlying layers of Bruch’s membrane (3). Appropriate maintenance of the RPE basal lamina (4, 5), as well as permeability of Bruch’s membrane as a whole (6, 7), are important determinants for retinal health.

Whereas loss-of-function mutations or premature stop codons in *EFEMP1* (the gene that encodes for F3) have been associated with the development of connective tissue diseases resembling Marfan syndrome (8–10), increased copy number/expression of *EFEMP1* correlates with increased risk for age-related macular degeneration (AMD) (11, 12). Moreover, autosomal dominant mutations in *EFEMP1* have been linked to eye diseases, including juvenile glaucoma (13, 14), primary open angle glaucoma (15), retinal degeneration (16), and a juvenile form of AMD called Malattia Leventinese/Doyne honeycomb retinal dystrophy (ML/DHRD) (17, 18). Presumably, autosomal dominant F3 mutations initiate their indicated associated disease by causing different degrees of protein misfolding (16, 19), increased F3 burden (i.e., altered extracellular/intracellular steady-state levels) (13, 20–22), changes to epithelial-mesenchymal transition (EMT) (23, 24), complement activation (5, 25, 26), or a combination thereof.

Yet, given F3’s broad ocular distribution, it may be surprising that both mice and humans lacking F3 show no clear defects in retinal structure or function (1). Moreover, removal of F3 appears to protect mice from environmentally induced basal laminar deposits (BLamDs) (27), extracellular masses that are harbingers of RPE stress in aging and in patients with AMD (3, 7). These results suggest that genetic or pharmacologic knockdown of F3 would be well-tolerated in the eye and that its removal may in fact protect against both autosomal dominant F3 diseases (e.g., glaucoma or ML/DHRD) and age-related diseases influenced by F3, such as AMD.

However, to date, no small-molecule therapeutics have been identified that can reduce the production of F3 from cells. Herein, we used CRISPR/Cas9 to genomically edit and tag endogenous F3 in cultured cells with an easily detectable peptide tag followed by high-throughput screening (HTS) for reducing compounds. We discovered that glycogen synthase kinase 3 (GSK3) appears to be a key node in regulating F3 production and is also responsible for controlling the expression of additional ECM proteins, particularly those involved in sub-RPE deposits. Excitingly, treatment of 8-month-old ML/DHRD R345W^+/+^ knockin mice with CHIR for 1 month significantly reduced R345W F3-associated BLamD number and size, thereby preventing the main pathological feature in these mice. Overall, these data strongly support the use of GSK3 inhibitors for reducing sub-RPE pathology associated with misfolded F3 and possibly in idiopathic AMD.

## Results

### Genome editing with HiBiT produces a sensitive method for following F3 production.

**Figure 1:**
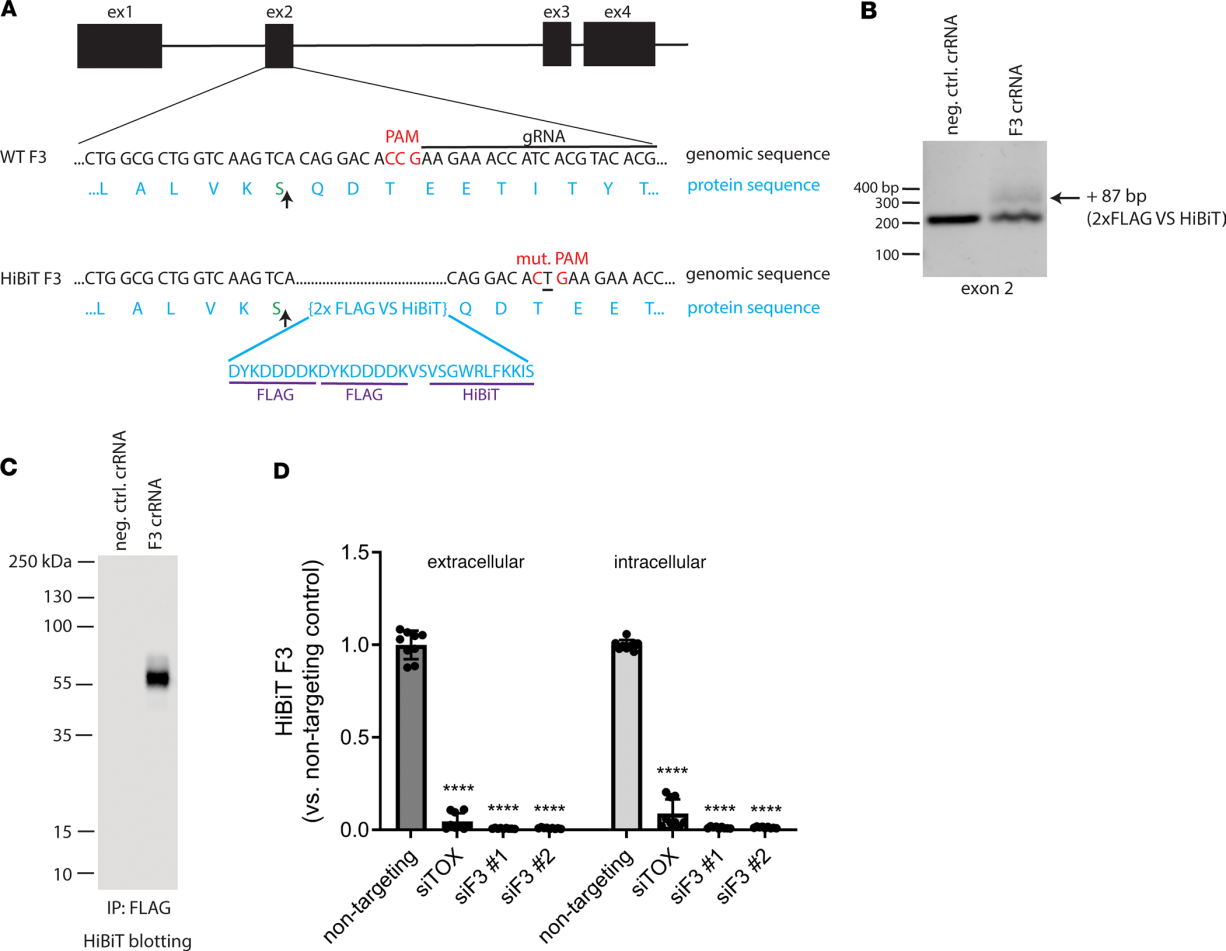
Design and validation of fibulin-3 HiBiT tagging in ARPE-19 cells using CRISPR. (**A**) Schematic of CRISPR editing of exon 2 of the fibulin-3 (F3) gene to knockin a 2xFLAG-VS-HiBiT sequence immediately proceeding the signal sequence cleavage site (upward arrow). (**B**) Successful editing was verified by gDNA amplification of exon 2. An additional band corresponding to insertion of the 87 bp 2x FLAG VS HiBiT tag was identified with a calculated editing efficiency of approximately 15%. (**C**) F3 HiBiT tagging results in a single extracellular protein species of correct molecular weight (~55 kDa), as identified by immunoprecipitation (FLAG beads) followed by elution and HiBiT blotting. (**D**) siRNA verifies that more than 95% of the HiBiT signal can be attributed to F3 gene translation. *n* = 3 independent experiments performed in biological triplicates. ****P ≤ 0.0001, 1-way ANOVA with Dunnett’s multiple comparison test vs. nontargeting siRNA.

As a secreted, ECM glycoprotein, endogenous F3 is typically difficult to detect in cultured cells (10) and in vivo (28). The challenge of monitoring F3 is compounded by its low levels, potential incorporation into the ECM, its monomeric molecular weight (55 kDa, the approximate size of BSA), and a dearth of F3 knockout-validated antibodies. We therefore designed a method to label endogenous F3 with an 11–amino acid HiBiT tag (29) (Figure 1A), which we theorized would facilitate quick and easy detection of endogenous F3 after complementation of HiBiT-tagged F3 with LgBiT and the NanoBiT substrate, furimazine, producing a bright and stable NanoBiT luminescent signal (29) proportional to F3 abundance. Using Sp. Cas9 ribonucleoprotein, we introduced a 2xFLAG-VS-HiBiT tag immediately after the F3 signal sequence through homology-directed repair (Figure 1A) in human adult retinal pigmented epithelial (RPE) cells (ARPE-19). This insertion was predicted to have no effect on F3 signal sequence processing (Supplemental Figure 1, A and B; supplemental material available online with this article; https://doi.org/10.1172/jci.insight.178050DS1) and avoided potential disruptions to F3 secretion by appending additional amino acids to the C-terminus of F3 (vis-à-vis select glaucoma mutations, ref. 13). Insertion of the HiBiT tag was verified by genomic DNA analysis, resulting in an amplicon with 87 additional base pairs (Figure 1B, ~15% editing efficiency). Validation of the specificity of the edit was assessed by using a scrambled negative control crRNA/tracrRNA duplex and performing immunoprecipitation of the 2xFLAG tag followed by HiBiT blotting, demonstrating a single species of approximately 55 kDa (Figure 1C) only in the F3 crRNA lane. Additional verification was accomplished by short interfering RNA (siRNA) knockdown of F3 (siF3 no. 1, siF3 no. 2) versus control siRNAs (nontargeting, Figure 1D). These observations suggest that our HiBiT editing approach is specific for F3 with little or no detectable off target effects.

### Miniaturization of the HiBiT F3 assay enables small-molecule HTS.

Increased F3 production has been associated with cancers such as gliomas (30) and prevalent vision disorders such as AMD (11, 12). Additionally, rare autosomal dominant, presumably gain-of-function, mutations in F3 have been associated with diseases such as juvenile open-angle glaucoma (13, 14) and the early-onset AMD-like disease, ML/DHRD (18). Accordingly, identifying genetic or small-molecule therapeutics designed to reduce F3 production could be therapeutically useful in these particular diseases. To facilitate this goal, we miniaturized the HiBiT assay into a 384-well format and performed HTS. An example mock plate (Supplemental Figure 2A) demonstrated consistent values in DMSO-treated wells (0.1%), with approximately 7% coefficient of variation for a whole-well analysis (HiBiT signal originating from both media and cells). We next screened HiBiT F3 ARPE-19 cells against a Prestwick Chemical Library (Alsace, France) and a NIH Clinical Collection. While we acknowledge that ARPE-19 cells do not accurately represent “true” RPE cells, as would be found in a human (31), we rationalized that they are appropriate to use from a simple cell biology perspective; they are a cell line that produces endogenous F3, adheres well to HTS plates (to increase consistency), and can be used at scale in HTS applications. Example data from 2 HTS plates (Supplemental Figure 2, B and C) demonstrate the consistency of the assay and highlight potential reducers and enhancers of F3 production in RPE cells, while Supplemental Figure 2, D and E, demonstrates the outstanding linearity of the HiBiT assay over 6 logs. Average *Z*′ score across all compound plates was excellent at 0.59 ± 0.06. One-hundred and twenty-seven F3 reducing compounds (*Z* score < –3) were identified in the primary screen (hit percentage = 127 of 1,646, 7.9% hit percentage). From these hits, 50 molecules were selected for confirmatory and counter screening. Compounds that affected HiBiT/LgBiT complementation or interfered with substrate binding to NanoBiT were identified in a counter screen using only HiBiT F3-containing media. Moreover, compounds that caused a more than 10% reduction in ATP levels (determined by a Cell Titer Glo 2.0 assay, Promega; Supplemental Table 4) over 48 hours at both 1.66 μM and 5 μM were excluded. For example, MG-132 was toxic at 5 μM, but not at 1.66 μM, and was therefore retained for subsequent dose-response assays. This triaging process yielded 8 hits (Supplemental Table 4), 7 of which were verified by dose response (Supplemental Figure 3, A and B). Within this set of verified hits, 2 compounds, AZD2858 and CHIR98014, were both identified as GSK3 inhibitors (32, 33). These compounds demonstrated dose-responsiveness (Supplemental Figure 3, A and B) and had favorable cytotoxicity profiles (Supplemental Table 4). Based on this enrichment within our data set, as well as the extensive use of GSK3 inhibitors for diverse diseases, including neurodegeneration (34, 35), we focused our efforts on characterizing inhibition of the GSK3 pathway as a unique way to regulate F3 production in cells.

**Figure 2:**
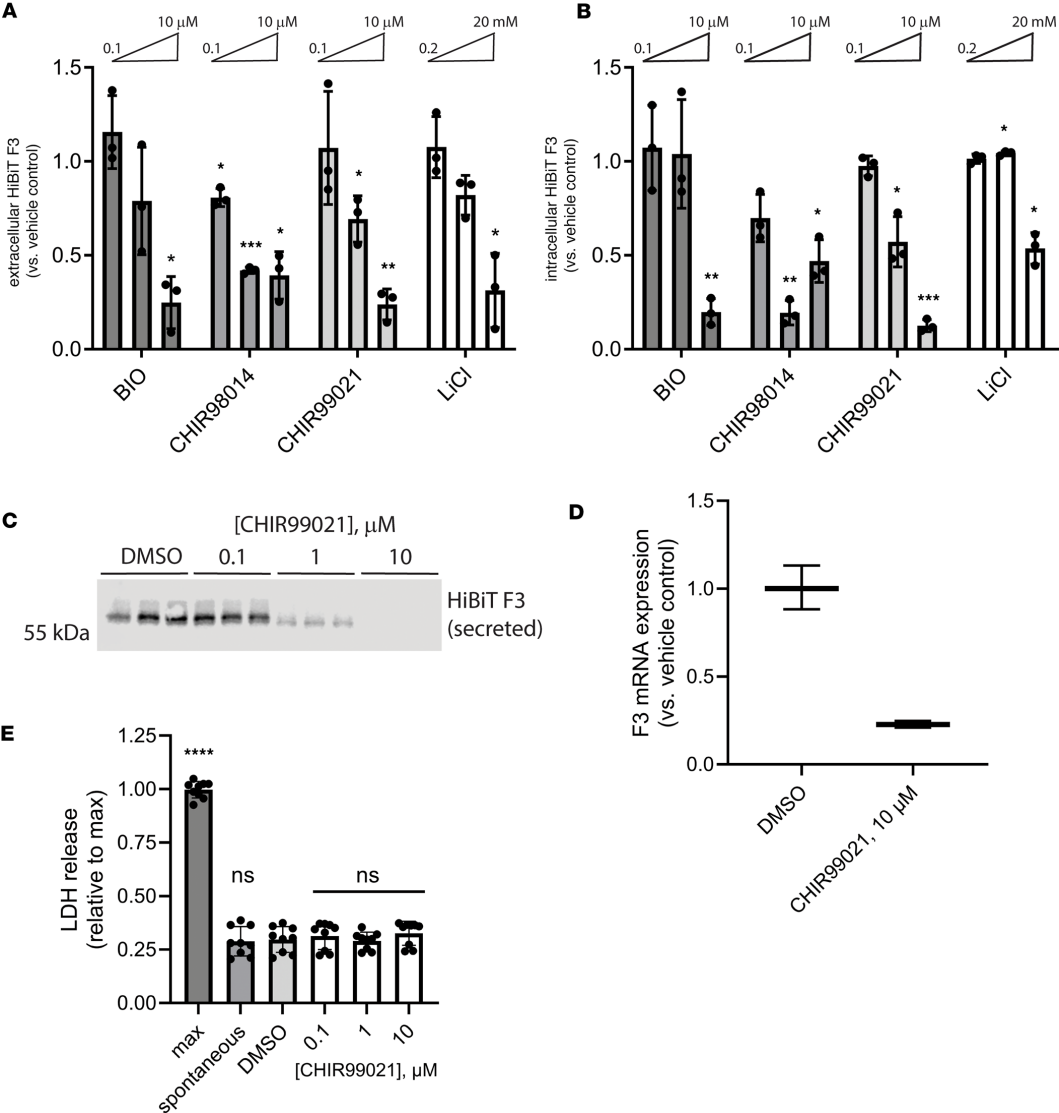
Structurally diverse glycogen synthase kinase 3 inhibitors reduce F3 transcripts and extracellular/intracellular levels without causing toxicity. (**A** and **B**) A series of chemically unrelated glycogen synthase kinase 3 (GSK3) inhibitors significantly reduced (**A**) extracellular and (**B**) intracellular HiBiT F3 levels after 72 hours of treatment. *n* = 3 independent experiments, mean ± SD. *P ≤ 0.05, **P ≤ 0.01, ***P ≤ 0.001, 1-sample *t* test vs. hypothetical unchanged value of 1 (vehicle treated). (**C**) CHIR99021-dependent HiBiT assay results in **A** were confirmed at the protein level by HiBiT blotting. Representative image from *n* ≥ 3 independent experiments. (**D**) Seventy-two-hour CHIR99021 treatment reduced F3 mRNA expression. Representative data from *n* = 3 independent experiments; mean ± SD of technical triplicates. (**E**) Treatment with CHIR did not elevate release of cytosolic lactate dehydrogenase (LDH). *n* = 3 independent experiments performed in biological triplicate. *P ≤ 0.05, ****P ≤ 0.0001, 1-way ANOVA with Dunnett’s multiple comparison test vs. vehicle-treated (DMSO) levels. BIO, 6-bromoindirubin-3-oxime; LiCl, lithium chloride.

In confirmatory assays, we validated the importance of the GSK3 pathway in regulating F3 production from RPE cells by expanding the diversity of GSK3 inhibitors tested, this time deconvoluting the whole well assay into secreted HiBiT F3 and intracellular HiBiT F3. Seventy-two-hour treatment with GSK3 inhibitors 6-bromoindirubin-3-oxime (BIO), CHIR98014, CHIR99021 (CHIR), and lithium chloride (LiCl) all significantly reduced HiBiT F3 secretion and intracellular levels (Figure 2, A and B) in a dose-responsive manner. Of these compounds, CHIR yielded the most consistent and effective results, reducing HiBiT F3 secretion up to 76% (10 μM, Figure 2A) and reducing intracellular HiBiT F3 up to 87% (10 μM, Figure 2B). Therefore, we prioritized subsequent testing of CHIR in blotting and quantitative PCR secondary assays. HiBiT blotting of conditioned media from RPE cells treated with CHIR (Figure 2C) confirmed the reduction in F3 secretion observed by HiBiT assay (Figure 2A). Moreover, it appears that CHIR reduced F3 secretion and intracellular levels by decreasing F3 transcription (23% ± 2% of vehicle-treated levels, Figure 2D). Moreover, lactate dehydrogenase (LDH) release assays confirmed that CHIR was nontoxic during these experiments (Figure 2E).

### siRNA knockdown of both GSK3α and GSK3β synergistically reduces F3 production.

**Figure 3:**
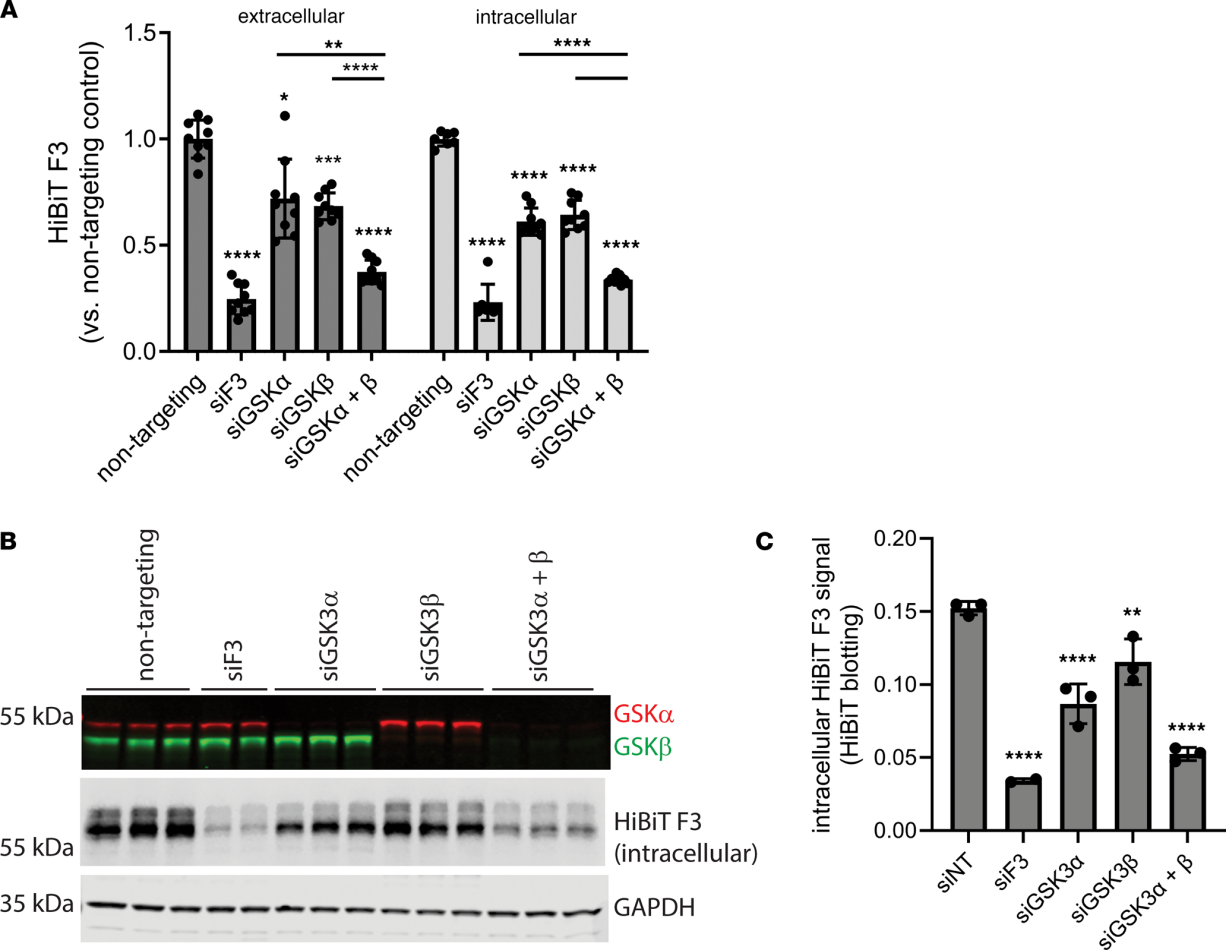
Genetic knockdown of GSK3 isoforms also reduces HiBiT F3 levels, paralleling pharmacologic GSK3 inhibitor effects. (**A**) Knockdown (96 h) of either GSK3α or GSK3β significantly lowered F3 production in ARPE-19 cells, and the effects of knocking down both α and β isoforms were additive. *n* = 3 independent experiments performed in biological triplicate. **P ≤ 0.01, ***P ≤ 0.001, ****P ≤ 0.0001, 1-way ANOVA with Dunnett’s T3 multiple comparison test. (**B**) Protein-level knockdown effects were verified by Western and HiBiT blotting, (**C**) followed by quantification of intracellular HiBiT F3. Representative images from *n* = 3 independent experiments. **P ≤ 0.01, ****P ≤ 0.0001, 1-way ANOVA with Dunnett’s multiple comparison test vs. siNT (nontargeting).

GSK3 has 2 structurally similar and potentially functionally redundant isoforms (36), GSK3α and GSK3β, both of which are produced in ARPE-19 cells (Figure 3, A and B) and both of which are targeted by many GSK3 inhibitors, such as CHIR, when it is used at elevated concentrations. To determine whether one GSK3 isoform was primarily responsible for reducing F3 production, we knocked down GSK3α and GSK3β individually and in combination by siRNA. Individual knockdown of GSK3α or GSK3β resulted in significant and nearly identical effects on HiBiT F3 secretion (~30% reduction, Figure 3A) and intracellular levels (~40% reduction, Figure 3A). Combination of GSK3α and GSK3β siRNAs further reduced HiBiT F3 levels in both the conditioned media (~55% reduction, Figure 3A) and cell lysates (~77% reduction, Figure 3A). GSK3α/β knockdown and HiBiT assay results were verified by Western/HiBiT blotting (Figure 3, B and C), demonstrating effective mRNA knockdown and providing an orthogonal confirmatory measurement of HiBiT F3 reduction. These results provide genetic confirmation that the CHIR-mediated effects on F3 are most likely occurring primarily through GSK3 inhibition.

### Testing the effects of CHIR in additional HiBiT-edited cell lines.

To determine whether the effects of GSK3 inhibition on F3 production were specific to ARPE-19 cells or represented a broader universal phenomenon conserved across cell lines, we genomically engineered two additional cell lines to express HiBiT F3: primary dermal fibroblasts and NIH-3T3 fibroblasts. Similar to ARPE-19 cells, CHIR significantly reduced both secreted and intracellular HiBiT F3 in human dermal fibroblasts in a dose-responsive manner (Supplemental Figure 4A). Moreover, long-term, 11-week treatment of primary porcine RPE cells with CHIR did not affect RPE morphology or tight junction formation (Supplemental Figure 4, A and B). Additionally, CHIR reduced F3 production in NIH-3T3 mouse cells as well (Supplemental Figure 5, A and B, and Supplemental Figure 6, A–C), demonstrating a cross-cellular and cross-species effects of CHIR.

Since F3 belongs to a family of similarly structured fibulin proteins (37), we subsequently tested if CHIR could also regulate other fibulin proteins, such as the highly homologous, short fibulin, fibulin-5 (F5 or *FBLN5*), or if its effect was more specific to F3. Using an identical HiBiT editing strategy (Supplemental Figure 7, A and B, and Supplemental Figure 8, A–D), we introduced a 2xFLAG HiBiT sequence onto the F5 protein in ARPE-19 cells and validated the specificity of editing using RNAi (Supplemental Figure 8B). Treatment of HiBiT F5-expressing cells with CHIR for 72 hours resulted in dose-responsive reduction of secreted F5 across all concentrations used (Supplemental Figure 8C). An extended, week-long treatment with CHIR at the same doses also significantly reduced F5 secretion, but only at 1 and 10 μM (Supplemental Figure 8D). However, high concentrations of certain GSK3 inhibitors can lead to a “rebound” effect, instead elevating F5 (Supplemental Figure 8D) or F3 levels (cf. Figure 2B, CHIR98014), possibly promoting intracellular retention or overriding the transcriptional reduction effect. Overall, these results suggest that the effects of CHIR on F3 appear to be cell line and species independent and that this compound may be acting more broadly on secreted or ECM proteins (e.g., fibulins) than initially thought.

### Low-level, long-term CHIR reduces HiBiT F3 secretion without triggering canonical Wnt signaling as indicated by T cell factor activation.

Regulation of GSK3 kinase activity is an important determinant in EMT decisions in multiple cell and in vivo contexts (38, 39). GSK3, in combination with casein kinase I, phosphorylates β-catenin and promotes its eventual degradation through the β-catenin destruction complex (40, 41). Thus, inhibition of GSK3 favors nuclear accumulation of β-catenin, which subsequently complexes with the transcriptional regulators lymphoid enhancing factor-1 and T cell factor (TCF), upregulating Wnt genes typically associated with increased EMT. Furthermore, complete genetic loss of *Gsk3* and, by extension, complete GSK3 inhibition in mouse progenitor cells leads to microphthalmia and overt ocular morphological defects (36). Moreover, EMT triggered by sustained high levels of GSK3 inhibition of RPE cells may play an important role in the pathology of retinal diseases (23, 42). Thus, we sought to determine whether low-level, longer-term CHIR treatment could still reduce F3 production but not trigger potential EMT through Wnt pathway activation.

**Figure 4:**
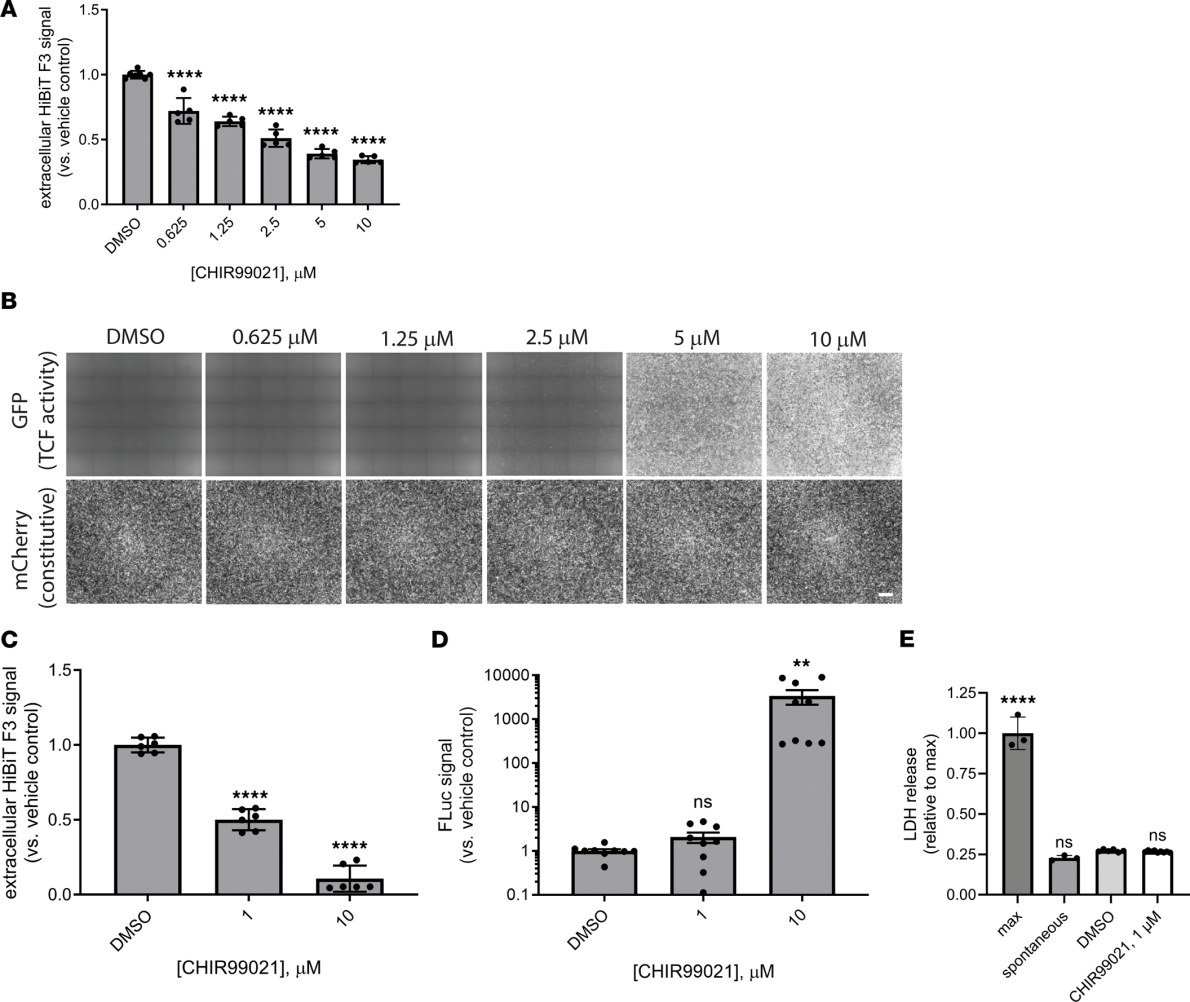
Low-level, prolonged CHIR99021 treatment reduces F3 production while avoiding triggering TCF4-dependent Wnt activation. (**A**) Seventy-two-hour CHIR99021 treatment dose-dependently reduced HiBiT F3 extracellular levels (**B**) without necessarily triggering TCF4-dependent green fluorescent protein (GFP) expression. *n* = 3 independent experiments performed in at least biological triplicate, with representative data presented. Scale bar: 200 μm. (**C**) One-week treatment with CHIR99021 (1 μM) significantly lowered HiBiT F3 extracellular levels (**D**) without activating TCF4-dependent firefly luciferase (FLuc). *n* = 3 independent experiments performed in biological replicates. (**E**) Prolonged low-level CHIR99021 did not increase cell death as indicated by LDH release. *n* = 3 independent experiments performed in biological replicates. *P ≤ 0.05, **P ≤ 0.01, ***P ≤ 0.001, ****P ≤ 0.0001, 1-way ANOVA with Dunnett’s multiple comparison test vs. vehicle (DMSO) treatment.

HiBiT F3-expressing RPE cells were transduced with lentivirus encoding for constitutively expressed mCherry and a green fluorescent protein (GFP) driven by 7 repeats of the TCF promoter (7TGC) or a puromycin-selectable lentivirus encoding for firefly luciferase driven by the same 7 TCF repeats (7TFP) (43). Treatment of the 7TGC HiBiT F3 cells with CHIR (0.675–10 μM) for 72 hours resulted in a predictable reduction in HiBiT F3 secretion (Figure 4A). Yet only concentrations greater than or equal to 2.5 μM resulted in any detectable GFP signal indicative of Wnt pathway activation (Figure 4B). Based on these findings, we next asked whether low-level CHIR treatment (≤1 μM) for longer periods of time would further reduce F3 production without triggering TCF activation. One-week treatment with CHIR significantly reduced F3 secretion at 1 μM (by 49.9% ± 7.0%, Figure 4C) without triggering TCF activation or toxicity (Figure 4, D and E). These data (as well as morphology data in primary porcine cells; Supplemental Figure 4, B and C) suggest that CHIR can be used at low concentrations to reduce F3 production while not detectably activating potentially detrimental Wnt/EMT-related signaling.

### Proteomics and transcriptomics confirm that low-level GSK3 inhibition reduces production of collagen, basement membrane, and ECM components.

Using our optimized CHIR dosage and treatment window, we next determined what additional secreted proteins were altered by low-level GSK3 inhibition. SDS-PAGE–separated concentrated media from CHIR-treated HiBiT F3 cells demonstrated a clear increase in total protein secretion at 10 μM but relatively little detectable change in banding pattern or intensity at 0.1 or 1 μM (Supplemental Figure 9). Proteomics unbiasedly determined that a total of 40 proteins were decreased by at least 50% (≥2-fold) compared with the vehicle control after 1 week of CHIR treatment (1 μM, Table 1, top 50 reduced proteins shown). F3 secretion was decreased in the proteomic data set by 37%, on par with our previous HiBiT observations (Figure 4C) but did not meet the 50% reduction inclusion criteria for this data set (Table 1). Nonetheless, notable proteins identified in this downregulated list include (a) collagens [collagen α-1(V), collagen α-2(IV), collagen α-1(IV), collagen α-3(IV)], (b) a major ECM protease [MMP2], (c) TGF-β–related proteins [plasminogen activator inhibitor 1 (PAI-1 or SERPINE1), latent TGF-β–binding protein 2 (LTBP2), and TGFβ1], (d) inflammatory/complement markers [high mobility group protein B1 (HMGB1), HMGB2, and complement component 3 (C3)], and (e) other members of the fibulin protein family [fibulin-1 (FBLN1) and fibulin-2 (FBLN2)]. Gene ontology (GO) analysis of the top 50 decreased proteins identified significant enrichment of proteins involved in collagen trimer formation, basement membrane, and ECM (Table 2). Conversely, more proteins were increased (154 proteins) by ≥2-fold (Supplemental Table 5, top 50 increased proteins shown) than were decreased. GO analysis of the top 50 of these proteins identified a range of cellular components including granule formation, chaperone complex, and ribosomal subunits (Supplemental Table 6).

**Figure 5:**
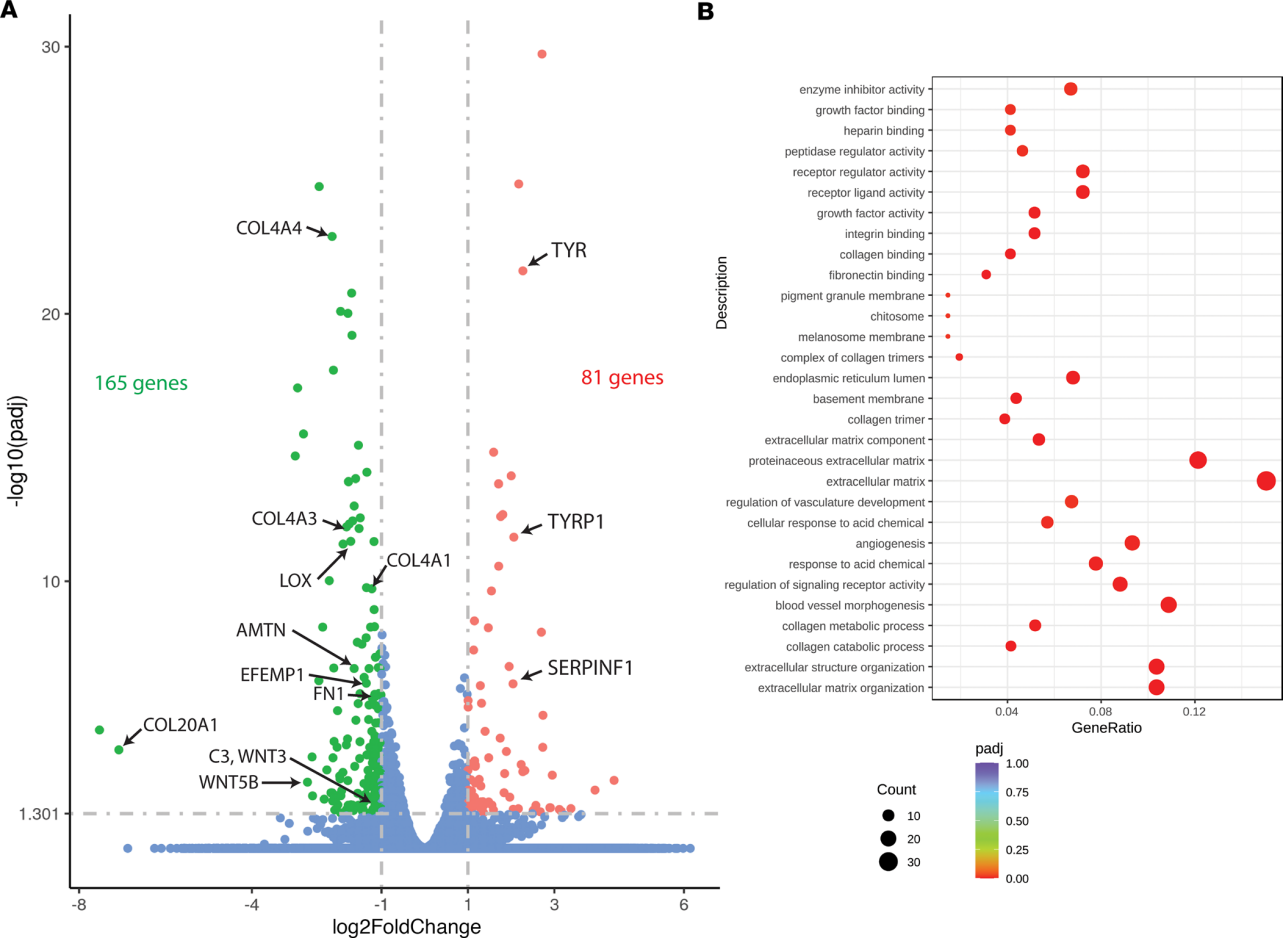
Low-level, 1-week CHIR99021 treatment reduces extracellular matrix and collagen-associated transcripts while upregulating RPE differentiation-associated genes. (**A**) Bulk RNA-Seq analysis of ARPE-19 cells treated with CHIR99021 indicated significant transcript reduction (≥2-fold) of genes implicated in extracellular matrix (ECM) formation and sub-RPE deposit formation while upregulating (≥2-fold) RPE differentiation genes. *n* = 3 independent experiments combined to produce these data. *P* adjusted < 0.05 using a negative binomial distribution model, green and red dots are significant. (**B**) Pathway enrichment of significantly altered genes from RNA-Seq data set using Gene ontology enrichment analysis.

In parallel experiments, RNA from HiBiT F3 cells treated identically with vehicle or CHIR for 1 week was sent for RNA-Seq analysis (Novogene). One-hundred and sixty-five genes were identified as being significantly downregulated by at least 50% (≥2-fold, Figure 5A and Supplemental Table 7), while 81 genes were identified as significantly upregulated by ≥2-fold (Figure 5A and Supplemental Table 8). Many of the proteins identified in the proteomic data set (e.g., COL4, F3, C3, etc.) were confirmed by RNA-Seq analysis (Supplemental Tables 7 and 8). Moreover, pathway analysis of genes that were significantly changed in either direction in the RNA-Seq data set further confirmed that low-level GSK3 inhibition with CHIR resulted in substantial changes to the production and organization of the ECM as well as the basement membrane composition (Figure 5B). Additionally, genes of interest pertaining to sub-RPE deposits, including amelotin (AMTN, ref. 44) and fibronectin (FN, ref. 45), ECM crosslinking enzymes, such as lysyl oxidase (LOX, ref. 46), and pro-EMT regulators, such as WNT3 (47) and WNT5A (48) (Figure 5A), were all downregulated. Interestingly, in the upregulated gene set, two important genes for pigment formation in the RPE (49), tyrosinase (TYR) and TYR-related protein 1 (TYRP1), were significantly upregulated by CHIR treatment (Figure 5A). An additional, potentially beneficial neuroprotective protein produced in the RPE (50), pigment epithelium-derived factor (PEDF, also known as SERPINF1), was also significantly upregulated in both the proteomics and RNA-Seq data sets by CHIR treatment (Supplemental Table 5 and Figure 5A). Similar observations regarding increases in TYR and PEDF have been made after supplementing CHIR during a human embryonic stem cell–to-RPE differentiation protocol (51).

### CHIR treatment reduces R345W F3-related pathology in vitro.

**Figure 6:**
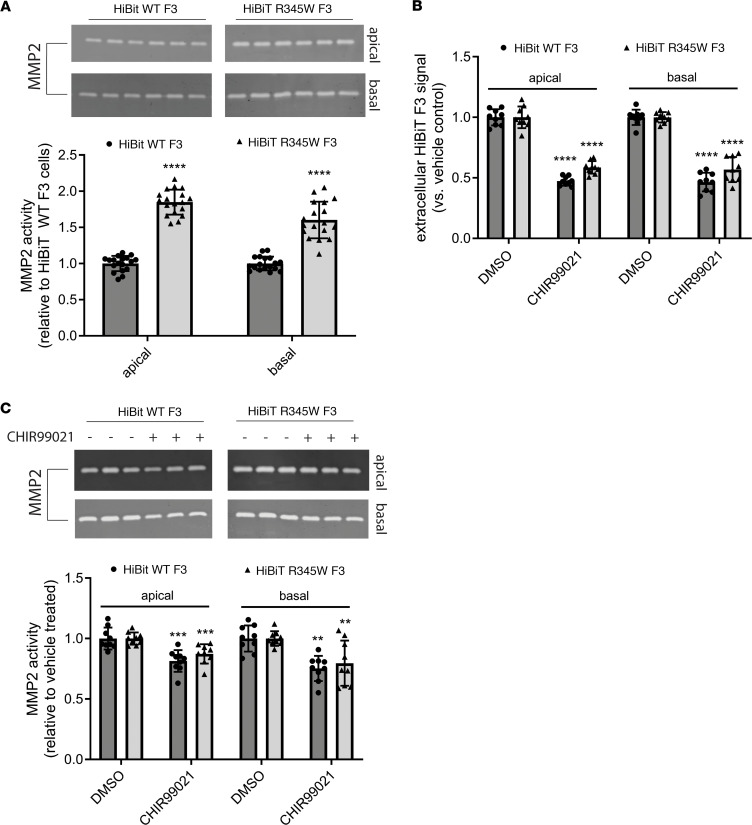
CHIR99021 reduces R345W F3-associated matrix metalloproteinase 2 alterations in culture. (**A**) ARPE-19 cells expressing HiBiT R345W F3 demonstrate significantly elevated apical and basal matrix metalloproteinase 2 (MMP2) activity levels compared with HiBiT WT F3 cells after 1 week on Transwells. *n* = 3 independent experiments, performed in sextuplet biological replicates. ****P ≤ 0.0001, t test vs. HiBiT WT F3 cells. (**B**) Treatment (1 week, 1 μM) with CHIR99021 significantly decreased HiBiT WT and R345W F3 both apically and basally in Transwell format (**C**) while simultaneously significantly decreasing MMP2 activity. *n* = 3 independent experiments, performed in triplicate biological replicates. **P ≤ 0.01, ***P ≤ 0.001, ****P ≤ 0.0001, *t* test vs. each respective vehicle-treated control.

Given the ability of CHIR to reduce several key phenomena that are associated with introduction of the R345W F3 ML/DHRD mutation in cells, humans, or mice (i.e., buildup of F3, refs. 21, 28, 52; collagen IV deposition, ref. 53; increased MMP2, ref. 5, 25; and activated C3, ref. 5, 54, 55), we decided to test whether this compound could serve as a multipronged approach to alleviate R345W F3-dependent pathology. To test this idea, initially we generated homozygous R345W-knockin ARPE-19 cells using CRISPR in a similar manner as described previously (5), isolated clonal edited cells, and then tagged R345W F3 in those cells with HiBiT, as described in Figure 1A (Supplemental Figure 10, A–C). HiBiT edited WT and R345W cells were seeded on Transwell inserts as described previously (5, 10) to promote cell polarization and ECM deposition in serum-free media. Consistent with previous observations (5), under untreated conditions, HiBiT R345W F3 cells demonstrated significantly increased apical (1.81- ± 0.18-fold) and basal (1.46- ± 0.15-fold) MMP2 levels based on zymography (Figure 6A). CHIR treatment (1 μM, 1 week) significantly reduced both apically and basally secreted HiBiT WT F3 and HiBiT R345W F3 (Figure 6B) to a similar extent to cells plated on plastic culture dishes (cf. Figure 4C). CHIR treatment also significantly reduced MMP2 levels in the apical and basal media of HiBiT WT F3 and HiBiT R345W F3 cells by 16%–17% in the apical media, and 29%–31% in the basal media (Figure 6C).

### CHIR enters the retina and demonstrates favorable pharmacokinetic properties.

Previous studies using CHIR in preclinical neurological disorders (i.e., bipolar disorder and Huntington’s disease) have demonstrated that it can cross the blood-brain barrier (56) and provide beneficial effects in the brain (57). However, no studies have validated whether it can also efficiently cross into the retina. Thus, we next performed pharmacokinetic (PK) studies to test CHIR penetrance into the mouse eye. CHIR trihydrochloride (25 mg/kg, i.p.) showed maximal distribution 30 minutes after i.p. dosing in plasma, liver, and retina (Table 3). In plasma and liver, CHIR was quickly metabolized/excreted (Table 3), yielding a plasma terminal half-life (*t*½) of 118 and 120 minutes, respectively, and a mean residence time of 49 minutes and 94 minutes, respectively (Table 4). Yet, levels of CHIR in the retina persisted, recording a substantially higher terminal *t*½ of 405 minutes and mean residence time of 363 minutes (Table 4). Based on the vitreous volume of the mouse eye (~4.4 μL, ref. 58), we estimated that the peak concentration of CHIR in the eye was approximately 1.3 μM after 30 minutes, falling to approximately 1.1 μM after 3 hours, 364 nM after 6 hours, and 151 nM after 24 hours. Thus, based on our cell culture observations, the concentration of CHIR falls within the range of concentrations capable to reduce F3 production but not trigger detectable Wnt activation.

### Prolonged in vivo administration of CHIR has no detrimental effect on retinal structure or function.

**Figure 7:**
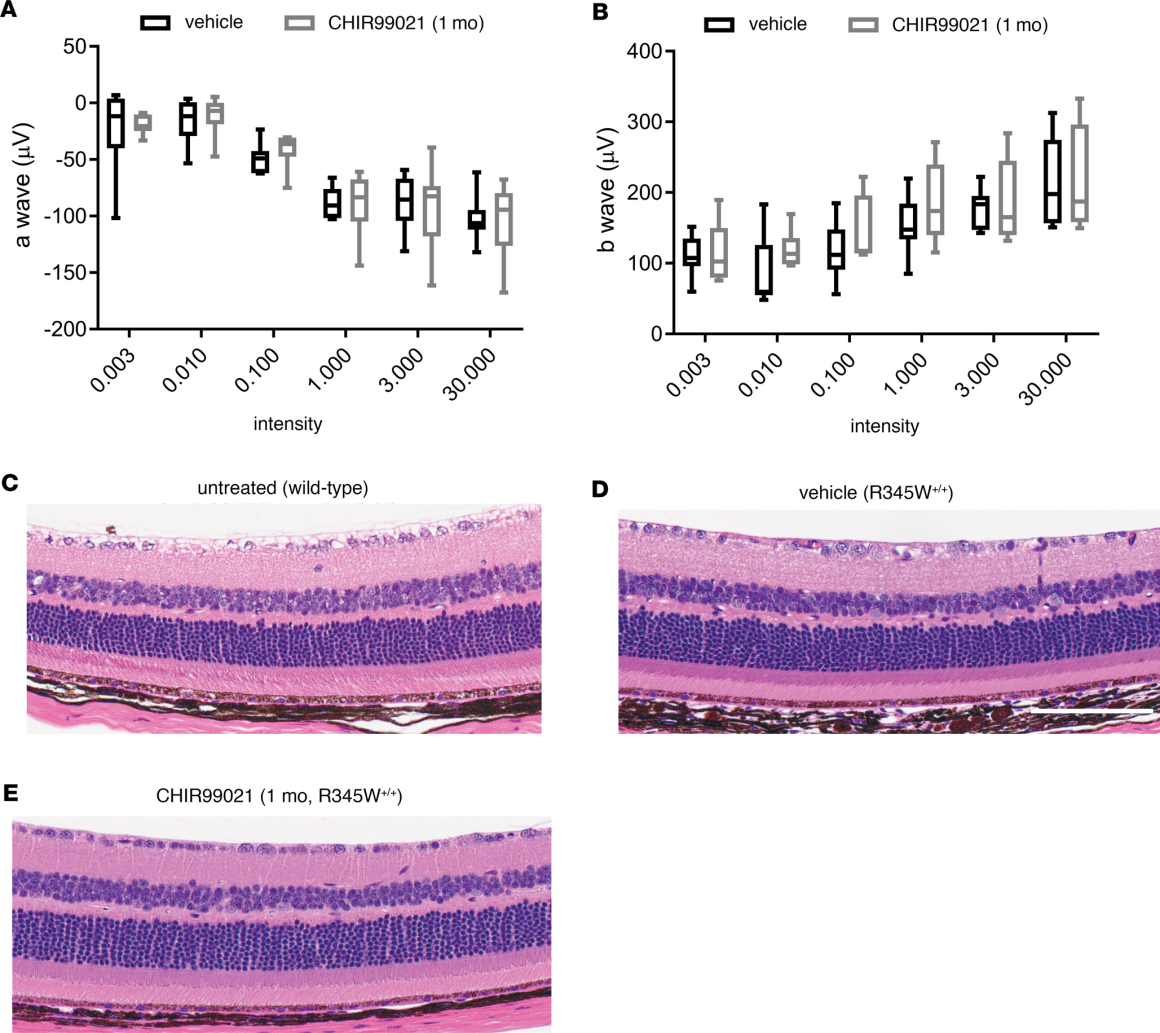
One-month CHIR99021 treatment does not affect retinal function or gross structure. (**A** and **B**) Eight-month-old R345W^+/+^ C57BL/6 mice were injected i.p. with vehicle (PBS) or CHIR99021 for 1 month (every weekday). Scotopic electroretinogram (ERG) readings demonstrated no difference between groups in either a-wave (outer retina, **A**) or b-wave (inner retina, **B**). Values were not significant by an ANOVA test. Box-and-whisker plots show average and minimum and maximum values. (**C**) An example H&E histology image of 8-month-old untreated WT C57BL/6 mice. (**D** and **E**) After completion of ERG evaluation, R345W^+/+^ mice were sacrificed and 1 eye was prepared for H&E histology, which demonstrated no observable differences between the 2 groups (vehicle, **D**; CHIR, **E**). *n* = 4 mice/treatment group, 2 male, 2 female. Scale bar: 100 μm.

Eight-month-old homozygous R345W (R345W^+/+^) F3-knockin mice (26) were treated with CHIR (25 mg/kg, i.p.) or vehicle every weekday for 1 month. We observed no physical changes (e.g., size, coat condition, or behavior) in the mice after CHIR treatment, consistent with previous studies (56, 57). Mice were then evaluated for scotopic electroretinogram (ERG) functional changes in their outer retina (photoreceptor cells, a-wave) or inner retina (ON bipolar cells, Muller cells, b-wave) at various light intensities. CHIR-treated mice showed no difference in ERG readings (a- or b-wave) when compared with vehicle-treated mice (Figure 7, A and B). Consistent with these functional observations, histology demonstrated no structural changes across all retinal cell layers (Figure 7, C–E), reaffirming the ocular safety of prolonged systemic CHIR treatment.

### CHIR reduces BLamD formation in R345W^+/+^ mice.

**Figure 8:**
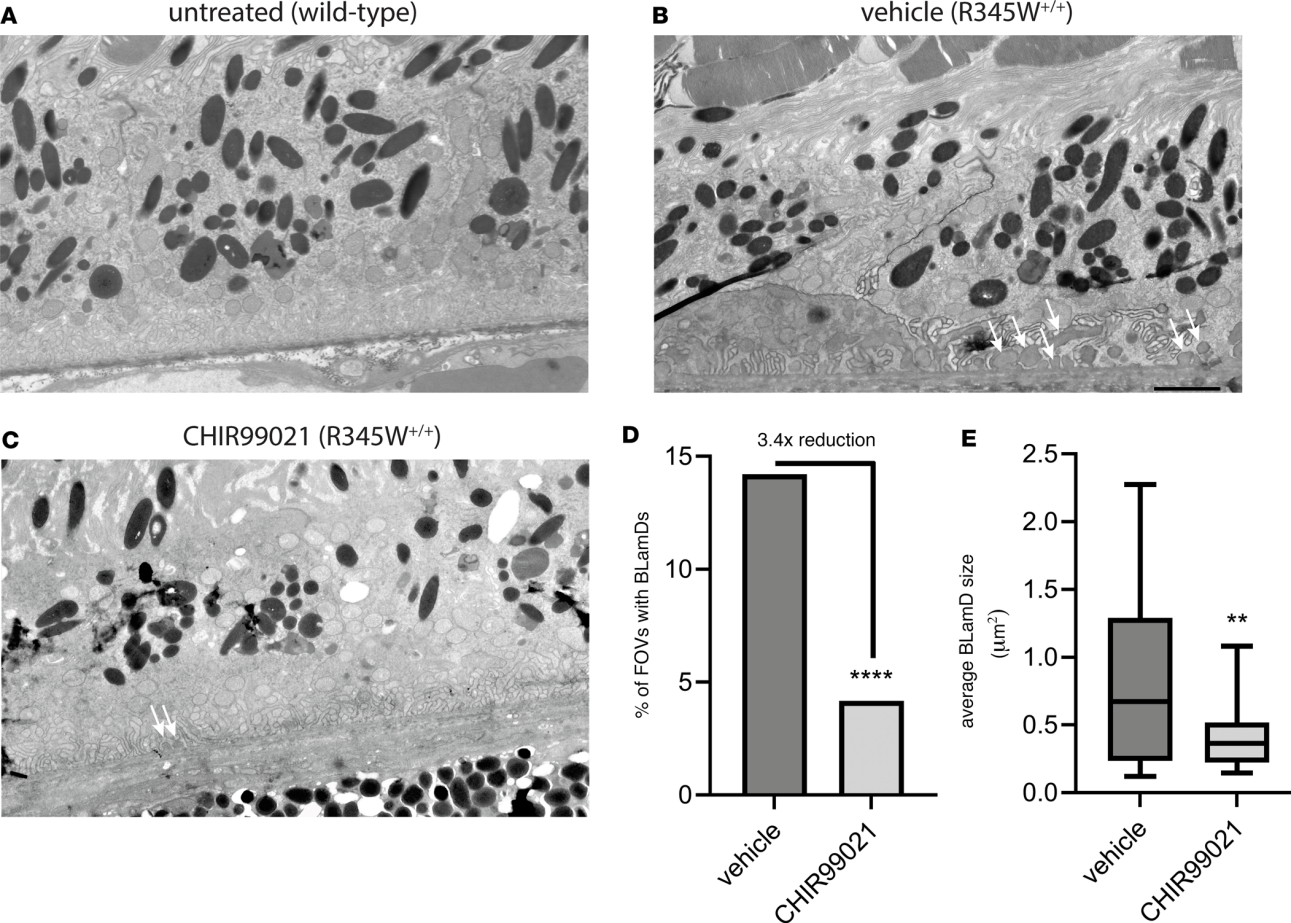
CHIR99021 significantly reduces the formation of basal laminar deposits in vivo. (**A**) Representative transmission electron microscopy (TEM) image of an 8-month-old WT C57BL/6 mice. (**B** and **C**) R345W^+/+^ mice after 1 month of vehicle (**B**) or CHIR99021 (**C**) treatment. Mice in **B** and **C** were 9 months old when sacrificed for basal laminar deposit (BLamD) (arrows) evaluation by TEM. Representative fields of view (FOV) of 22 fields are presented for vehicle or CHIR99021 treatment. Scale bar: 2 μm. (**D** and **E**) A masked observer systematically quantified (**D**) the number of FOV containing any BLamD (****P ≤ 0.0001, χ^2^ test) as well as (**E**) the average size of the BLamD, if at all present (**P ≤ 0.01, *t* test vs. vehicle-treated samples).

The canonical pathologic feature of the ML/DHRD macular dystrophy mouse model is the formation of sub-RPE BLamDs that increase with R345W F3 gene dosage and age (26, 52). Given the ability of CHIR to reduce the production of proteins associated with sub-RPE deposits (e.g., F3, FN1, AMTN, C3, and collagens) from cultured cells, we next asked whether CHIR treatment could prevent or slow the formation of BLamDs in vivo. Eight-month-old R345W^+/+^ mice were treated for 1 month as described above. At this age, R345W^+/+^ mice form a few isolated BLamDs (see Figure 8A for a WT comparison), some of which begin to coalesce to form continuous BLamDs across transmission electron microscopy fields of view (FOVs, Figure 8B) (26, 52). Thus, the BLamDs that form in these mice represent an early, but symptomatic, stage of retinal disease progression (3). One month of CHIR treatment significantly reduced the number of BLamDs formed, decreasing number of FOVs containing deposits from 14.205% (125 of 880 FOVs) in untreated mice (Figure 8, B and D) to 4.167% (30 of 720 FOVs) in treated mice (Figure 8, C and D). Example FOVs in which BLamDs were not observed can be found in the supplemental material (Supplemental Figure 11, A and B). Additionally, in FOVs in which BLamDs were observed, their average size was also significantly reduced from 0.817 μm^2^ in untreated mice to 0.416 μm^2^ in CHIR-treated mice (Figure 8E). These results are an important demonstration of small-molecule–mediated reduction in BLamD formation in mice, paving the way for future studies in additional preclinical models of macular dystrophy/macular degeneration characterized by sub-RPE deposit formation.

## Discussion

Herein, we have highlighted what we believe is the first small-molecule–based treatment that can prevent AMD-like pathology associated with ML/DHRD, an aggressive and currently incurable retinal disease that can affect individuals as young as 12 years of age (26). Natural history studies have indicated that ML/DHRD onset can vary substantially, even among siblings (59), which suggests that disease pathology is likely modifiable by genetic and/or environmental alterations. Reaffirming this idea, at least one 62-year-old male harboring the R345W mutation was identified as asymptomatic (59). The observations that ML/DHRD onset and severity are likely pliable are consistent with our findings that a small molecule, such as CHIR, can substantially affect R345W-dependent pathology both in vitro and in vivo. While the prevalence of ML/DHRD is indeed rare, with an unknown number of patients worldwide, like many inherited retinal degenerations, ML/DHRD is likely underdiagnosed. In fact, in some European populations, it may comprise up to 0.9% of all inherited retinal degenerations (60), amounting to over 3,000 affected individuals in Europe alone, representing a significant unmet patient population that could benefit from new therapeutic approaches.

More broadly, our observations that CHIR reduces BLamD formation in the ML/DHRD mouse model suggest that it may be possible that the main pathogenic feature of early and intermediate AMD (e.g., sub-RPE drusen deposits) is manipulatable using a single small molecule. Theoretically, a small molecule like CHIR, which we demonstrate reduces F3 transcription, would be preferred to alternative small molecules that inhibit F3 secretion (which could initiate stress response activation) or degradation-promoting molecules (which would need to affect ER-resident or secreted F3). Interestingly, there is precedence in translating pathology reduction in the ML/DHRD mice to meaningful benefit in humans with AMD. For example, a previous study indicated that genetic elimination of C3 also prevented BLamD formation in ML/DHRD mice (26). Just last year, pegcetacoplan (Syfovre) (61), a C3 peptide inhibitor, was approved as one of only two FDA-approved drugs to minimize progression of geographic atrophy in AMD. Thus, we are hopeful that our own encouraging findings in the ML/DHRD mouse model will contribute toward the development of therapeutic advances aimed at early and intermediate stages of AMD. Of course, one challenge of translating findings in mice to humans is that while mouse vision is primarily rod-centric, human vision is more complex, involving both rods and cones (the latter is more affected in AMD). Nonetheless, we hypothesize that therapies such as CHIR have the potential to delay the advancement of early and intermediate stages of AMD (affecting ~150 million individuals worldwide, refs. 62, 63) to later stages such as geographic atrophy and, thus, may be able to prevent eventual vision loss associated with dry AMD.

Our chemogenetic data strongly suggest that CHIR is primarily working through GSK3 inhibition. Yet, even after the identification of GSK3 40 years ago (64), its role(s) in regulating fundamental cellular processes, including cellular architecture, differentiation, cell fate, gene expression, and energy metabolism, and others (65, 66), is still being defined. Given its wide range of targets and biological effects, perhaps it is not surprising that GSK3 overactivity has been implicated in diverse diseases, including neurodegenerative diseases (e.g., Alzheimer’s disease, refs. 67, 68; Parkinson’s disease, ref. 69; and myotonic dystrophy, ref. 70) as well as psychiatric disorders (e.g., unipolar and bipolar affective disorders, refs. 71, 72). Accordingly, a plethora of GSK3 inhibitors have been developed (73) and safely used in multiple preclinical systems (73), even in models of retinitis pigmentosa (74, 75), optic nerve regeneration (76), and steroid-induced glaucoma (77). However, translating these observations into humans has been met with varying success (56, 78). As a result, LiCl, a commonly used antipsychotic (71, 72, 79), remains the only FDA-approved GSK3 inhibitor. Yet, its narrow therapeutic window all but prevents its use for neurodegenerative diseases (71, 72). Thus, there is a substantial opportunity to either develop more efficacious GSK3 inhibitors or to apply existing inhibitors for new diseases, such as ML/DHRD or AMD.

Given the wide number of GSK3 phosphorylation targets, it is likely that CHIR-mediated prevention of BLamD formation could originate from not just a single phenomenon such as a reduction in F3 transcription, as we had envisioned, but rather a combination of reduced F3 burden, ECM compositional changes, gene expression alterations (44, 80–82), and possibly metabolic remodeling (83, 84). Moreover, it is not clear whether broad GSK3 inhibition across tissues (or the body, for that matter) is necessary for the BLamD prevention we observed or if GSK3 inhibition in a particular retinal cell layer (i.e., in the neural retina vs. RPE vs. choroid) is sufficient to achieve BLamD reduction. Elucidation of these critical questions will enable more precise and targeted GSK3-based therapeutics while avoiding potential systemic side effects of broad GSK3 inhibition (78, 85). Ultimately, testing CHIR in nonhuman primates or humans to reverse existing sub-RPE deposit formation will serve as a challenging, but necessary, next endeavor.

## Methods

### Sex as a biological variable.

Our study examined male and female mice, and similar findings are reported for both sexes.

### Expanded methods.

For more in-depth experimental methods, please refer to the corresponding Supplemental Methods.

### Cell culture.

ARPE-19 cells (CRL-2302, ATCC) were cultured in DMEM/F12 (Corning) with 10% FBS (Omega Scientific) and 1% PSQ (Thermo Fisher Scientific). HEK293T cells (Life Technologies) were cultured in high-glucose DMEM (4.5 g/L) supplemented with 10% FBS and 1% PSQ. Mouse NIH-3T3 fibroblasts (CRL-1658, ATCC) were maintained in high-glucose DMEM with 10% calf serum and 1% PSQ. Primary dermal fibroblasts (PCS-201-012, ATCC) were maintained in either low-glucose DMEM with 10% FBS and 1% PSQ or fibroblast growth media (Fibroblast Basal Medium [PCS-201-012, ATCC] supplemented with Low-Serum Fibroblast Growth Kit [PCS-201-041, ATCC]).

### HiBiT F3 cell line generation.

ARPE-19 cells were genomically edited to introduce a 2xFLAG-VS-HiBiT sequence immediately after the signal sequence cleavage site (Ser16) of F3 (Figure 1A). This tag was not predicted to affect F3 signal sequence cleavage (Supplemental Figure 1, A and B), and similar strategies have been used previously (86). An in-depth description of our editing process has been published elsewhere (87). Briefly, Alt-R Sp. Cas9 Nuclease V3 (Integrated DNA Technologies, IDT) loaded with a crRNA/tracrRNA duplex (Supplemental Table 1) and accompanied by a single-stranded oligodeoxynucleotide template (Supplemental Table 1) were electroporated introduced into ARPE-19 cells (1400 V, 20 ms, 2 pulses, Neon Transfection System, Life Technologies). Cells were then incubated in antibiotic-free media containing a homology-directed repair enhancer (48 hours). Cultures were expanded, assayed for HiBiT, and verified genomically(Figure 1B, primers listed in Supplemental Table 2).

### HTS.

HiBiT F3 ARPE-19 cells were seeded at a density of approximately 5,000 cells/well with a MultiFlo (Agilent) in a white 384-well plate (Greiner) and incubated at 37°C for 24 hours. Media were changed, and compounds from the Prestwick Chemical Library (1,200 compounds) and the NIH clinical collection (446 compounds) were added using an Echo 655 (Beckman Coulter) at a final concentration of 5 μM and 0.1% DMSO. As a positive control for reduction of F3, brefeldin A (50 μM), was used. Twenty-four hours after compound addition, plates were cooled to room temperature followed by a whole-well HiBiT assay (Promega) on an Envision plate reader (PerkinElmer). HiBiT assay performance was calculated by determining the Z factor (Z’) (88).

Hit compounds were identified as having HiBiT F3 signal less than 3 SD from plate mean. A counter screen was used to identify toxic compounds Cell Titer Glo 2.0 (Promega).

Media alone was treated with the hit compounds to identify any false reductions caused by NanoBiT inhibition. Remaining hit compounds were verified in a confirmatory screen using fresh compound from a new source plate.

### siRNA.

siRNA knockdown was used to verify the specificity of CRISPR HiBiT editing. siRNAs (100 nM final concentration, Silencer Select, Ambion, Supplemental Table 3) were introduced into cells by reverse transfection. For a 24-well plate, 2.14 μL DharmaFECT4 (Horizon) was diluted into 250 μL OptiMEM (Thermo Fisher Scientific). Mixtures were vortexed for 15 seconds and then incubated at room temperature for 20 minutes. ARPE-19 cells were trypsinized and normalized to a density of 466,000 cells/mL in media. Two-hundred and fifty microliters of the siRNA/OptiMEM/DharmaFECT4 complex was added to a 24-well plate, after which an equivalent volume of cell suspension was added. After 24 and 72 hours following knockdown, the media were changed. HiBiT levels were determined the next day (96 hours after knockdown) via an extracellular and intracellular HiBiT assay.

### Non-HTS HiBiT assay.

Cells were plated at a high density of 200,000 cells/mL for all non-HTS HiBiT assay experiments. The following day, media containing the indicted compound was added and incubated for either 72 hours or 1 week. For 1-week treatment wells, media were replaced after 96 h with fresh media containing compound. After treatment, extracellular and intracellular assays were performed to determine levels of HiBiT-tagged F3 using the Nano-Glo HiBiT Detection Kits (Promega) according to standard assay parameters (read on a Synergy 2 (BioTek) or a GloMax (Promega)).

### Western blotting.

Cells were rinsed with HBSS followed by lysis (5–15 minutes) in buffer containing radioimmunoprecipitation buffer (RIPA, Santa Cruz), protease inhibitor (Pierce), and benzonase (MilliporeSigma) or the Nano-Glo HiBiT Lytic Detection System (Promega). The samples were spun at 4°C for 10 minutes at 21,000*g*, collecting the soluble supernatant. Where possible, a bicinchoninic acid assay (Pierce Thermo Scientific) was used to quantify protein levels and normalize them to 20 μg. For cells lysed in HiBiT Lytic buffer, samples were normalized by volume (due to DTT). Samples were boiled in reducing Laemmli buffer (5 minutes) and then loaded onto a 4%–20% Tris-Gly SDS-PAGE gel and run at 140 V (80 minutes). Proteins were transferred to 0.2 μm nitrocellulose membrane (P0 protocol, iBlot2, Life Technologies). Total protein was visualized with Ponceau S (Sigma-Aldrich), and blots were incubated overnight in blocking buffer (Intercept Blocking Buffer, LI-COR). Blots were then incubated for 1 hour at room temperature with primary antibody (GSK3α [sc-5264, Santa Cruz], GSK3β [9315S, Cell Signaling], or GAPDH [sc-47724, Santa Cruz]) diluted in 5% BSA in Tris-buffered saline (TBS) and 0.05% NaN_3_. Blots were rinsed in TBS with 0.05% Tween (TBS-T) and incubated for 40 minutes at room temperature in an appropriate anti-mouse or anti-rabbit secondary antibody (1:10,000 to 1:15,000, LI-COR) dissolved in 5% milk in TBS-T. Blots were washed again, imaged on an Odyssey CLx (LI-COR), and analyzed using ImageStudio Software (LI-COR). Full, uncropped blot and gel images are located in Supplemental Figure 12.

### HiBiT blotting.

Samples were lysed, prepared, separated by SDS-PAGE, and transferred to nitrocellulose membranes as described above for Western blotting. Once transferred, the membrane was incubated in TBS-T (10 minutes to 1 hour, room temperature). Blots were then incubated with blotting buffer containing 1:200 LgBiT for 1–2 hours, with rocking at room temperature (N2410, Promega). NanoGlo substrate was added (1:500), and the blots were incubated for 5–10 minutes (room temperature). Chemiluminescence was imaged on an Odyssey Fc (LI-COR).

### LDH release assay.

Cells were treated with CHIR for 72 hours or 1 week. Media were then collected and spun at 100*g* for 5 minutes. Fifty microliters (technical triplicates) of cleared media were mixed with an equal amount of LDH reaction buffer (LDH Cytotoxicity Assay Kit, Pierce) in a clear 96-well plate for 30 minutes at 37°C. A DMSO-treated media sample served as a control while cells treated with cell lysis buffer (provided in the LDH Cytotoxicity Kit) served as a positive control. Fifty microliters of stopping solution was added to each well, and absorbance at 490 nm was read (GloMax).

### 7xTCF-eGFP mCherry and 7xTCF-firefly luciferase puromycin lentivirus production.

VSV-g-pseudotyped replication incompetent lentivirus was generated as we have described previously (89, 90). Briefly, lentiviral plasmids (Addgene plasmids 24304 and 24308, gifts of Roel Nusse, ref. 43) were cotransfected with psPAX2 and VSV-g plasmids (Addgene plasmids 12260 and 12259, gifts of Didier Trono) into HEK293T cells with Lipofectamine 3000 (Life Technologies). The next day, media were changed, followed by collection 24 hours and 48 hours later. To establish 7xTCF-eGFP mCherry (7TGC) and 7xTCF-firefly luciferase puromycin (7TFP) cell lines, HiBiT WT F3 ARPE-19 cells were plated at 1 × 10^6^ cells/well of a 6-well plate and infected with lentivirus in full media containing polybrene for 24 hours. 7TFP cells were selected with puromycin (1 μg/mL) for 1–2 weeks.

### Firefly luciferase assay.

HiBiT WT F3 7TFP ARPE-19 cells were treated with CHIR for 1 week (with a 72-hour media change) and assayed for firefly luciferase expression. Briefly, after treatment, cells were washed with HBSS. Fifty microliters lysis buffer (Firefly Luciferase Glow Assay, Pierce) was added to each well and mixed in plate (15 minutes). Fifty microliters of working solution containing luciferin and Firefly Glow Assay Buffer was added to the wells of a black 96-well plate followed by 10 μL lysate and read on a Synergy2 plate reader.

### MMP2 zymography.

HiBiT WT F3 or HiBiT R345W F3 ARPE-19 cells were plated at a density of approximately 100,000 cells per well of a 12-well, 0.4 μm polyester Transwell insert (Corning), or approximately 30,000 cells per well of a 24-well, 0.4 μm polyester Transwell insert (Corning). Media were changed to serum-free media the following day and every 3–4 days thereafter. After 1 week, cells were treated with either DMSO (0.1%) or 1 μM CHIR. After 2 weeks on Transwells (1-week treatment), an extracellular HiBiT assay and MMP2 zymography were performed. Briefly, for zymography, media from the apical and basal chamber were collected and combined with nonreducing SDS buffer, followed by running on a 10% gelatin gel (Novex) for 90 minutes at 140 V. MMP2 was renatured in buffer (G-biosciences) for 30 minutes (room temperature). Gels were changed to developing buffer (30 minutes, room temperature, G-Biosciences). Fresh developing buffer was added, and gels were shaken overnight (37°C). Gels were stained in Coomassie R-250 for 1 hour, destained, and imaged on an Odyssey CLx (LI-COR). Bands were quantified using Image Studio software (LI-COR).

### Secreted proteome visualization and mass spectrometry.

HiBiT WT F3 ARPE-19 cells were treated with CHIR for 72 hours in serum-free media. Conditioned media were concentrated (Amicon Ultra 3,000 MWCO, MilliporeSigma) and run on a 4%–20% Tris-Gly SDS-PAGE gel for 80 minutes at 140 V. Protein bands were imaged by silver staining (SilverQuest Silver Staining Kit, Invitrogen). In parallel, an aliquot of the concentrated media sample was run for 10 minutes at 140 V on a 4%–20% Tris-Gly SDS-PAGE gel, stained with Coomassie Blue, and the single band corresponding to total secreted protein was excised and submitted for mass spectrometry (University of Texas Southwestern Medical Center Proteomics Core). See Supplemental Methods for exact mass spectrometry conditions.

### Sample preparation for RNA-Seq.

HiBiT WT F3 ARPE-19 cells were plated at 200,000 cells/mL in DMEM/F12 media with 10% FBS and 1% PSQ. The following day, media containing DMSO (1:1000) or 1 μM CHIR were added to the cells. Cells were treated for 96 hours before an additional media change. Seven days after beginning treatment, RNA was extracted using an Aurum Total RNA Kit (Bio-Rad) and stored at –80°C. Fifteen microliters of sample was provided to Novogene. One microgram of RNA was used as input material for library generation and analysis (listed in greater detail in Supplemental Methods).

### Animal housing, handling and approval.

All mice used in these studies were either obtained directly from The Jackson Laboratory (such as for the PK experiments listed below) or were housed under 12-hours-on/12-hours-off light cycles and fed standard rodent chow/water ad libitum at University of Texas Southwestern Medical Center or the University of Minnesota. Sex as a biological variable was considered, and equal numbers of males and females were used wherever possible.

### CHIR PK experiment.

C57BL/6J mice were dosed via i.p. injection with CHIR trihydrochloride (Tocris) dissolved in 2% DMSO with 5.7% Captisol (Ligand Pharmaceuticals) in 1x PBS at a concentration of 5 mg/mL. Mice were dosed at 25 mg/kg. The dosing time points were 30, 180, 360, 960, and 1440 minutes, with a 0-minute control group. Each time point included 3 mice, with at least 1 male and 1 female mouse in each group. At select time points, isoflurane was used to anesthetize mice prior to tissue collection. Plasma, liver, and neural retina (both eyes pooled) were collected, and the samples were flash frozen in liquid nitrogen. All samples were then sent to the University of Texas Southwestern Medical Center Preclinical Pharmacology Core, and the amount of CHIR was measured in each tissue using LC-MS/MS (see below and Supplemental Methods).

### LC-MS/MS analysis of CHIR.

Retina tissue, liver tissue, and plasma were analyzed for CHIR concentrations using an LC-MS/MS method. Retinas and livers were homogenized in PBS. Retina homogenates were made using BeadBug prefilled tubes with 3.0 mm Zirconium beads (Z763802, MilliporeSigma) and a BeadBug microtube homogenizer run for 1 minute at 800*g*. For standards, blank commercial plasma (Bioreclamation) or untreated liver or brain tissue homogenate was spiked with varying concentrations of compound. In-depth methods are included in the Supplemental Methods. Back-calculation of standard curve and quality control samples were accurate to within 15% for 85%–100% of these samples at concentrations ranging from 0.5 ng/mL to 10,000 ng/mL.

### In vivo treatment of CHIR.

As the PK experiment demonstrated successful penetration of CHIR into the retina, we used the same CHIR formulation for in vivo treatment of C57BL/6 R345W^+/+^ knockin mice. Eight-month-old R345W^+/+^ mice (26) were divided into a treatment group and control group (*n* = 4 mice/group, 2 male and 2 female mice). CHIR (25 mg/kg) or PBS (vehicle) was delivered via i.p. injections given every 24 hours during the 5-day work week for 1 month.

### ERG.

After the 1-month vehicle or CHIR treatment, scotopic ERG was performed. Mice were dark-adapted the night before ERG. A-waves and b-waves were monitored in a dark-adapted intensity series using 6 different light intensities (0.003–30 cd.s/m^2^, Celeris, Diagnosys). Each mouse was weighed and given a ketamine/xylazine solution diluted 1:1 in biostatic water (120 mg/kg and 16 mg/kg, respectively, final concentration). Once mice were unresponsive to a toe pinch, tropicamide (1% w/v, Akorn) was administered to dilate the pupil. A probe was placed into the skin between the eyes, and a ground wire was placed into the skin near the base of the tail. GenTeal Severe Dry Eye gel (Alcon) was placed onto each eye, and electrodes were aligned to face the retina. Interference was reduced to below 8.0 kΩ before starting the scan. This process was repeated for each mouse, and the a- and b-wave amplitudes were provided by the Espion software (Diagnosys). Outlier data points were identified using GraphPad Prism.

### Statistics.

Statistical tests (aside from RNA-Seq data) were all performed in Prism (GraphPad) or Excel (Microsoft). For most multiple-comparison tests, a 1-way ANOVA with Dunnett’s (or Dunnett’s T3) multiple-comparison adjustments was conducted. For some experiments where comparisons were to a hypothetical unchanged value of 1, a 1-sample, 2-tailed *t* test was performed. For single pairwise comparisons, a 2-sample, 2-tailed *t* test assuming equal variance was conducted. A χ^2^ test was performed to assess presence or absence measurements. RNA-Seq data were analyzed by Novogene using a negative binomial distribution model. *P* values of less than 0.05 were considered significant.

### Study approval.

All animal procedures were conducted under an Institutional Animal Care and Use Committee–approved animal protocol from the University of Texas Southwestern Medical Center or the University of Minnesota and followed the Association for Research in Vision and Ophthalmology guidelines for the Use of Animals in Ophthalmic and Vision Research.

### Data availability.

RNA-Seq data have been deposited into the NIH BioProject database (http://www.ncbi.nlm.nih.gov/bioproject; BioProject ID PRJNA1123278). The mass spectrometry proteomics data have been deposited to the ProteomeXchange Consortium (http://proteomecentral.proteomexchange.org) via the MassIVE partner repository, with the data set identifier PXD052966. Supporting data values for all data points shown on graphs (rounded to the hundredths or thousandths position for simplicity in instances) are reported in the Supporting Data Values file.

## Author contributions

SMD designed and performed the majority of experiments, analyzed data, and wrote the manuscript. AJO, GEC, SD, and MKM performed experiments and analyzed data. KNT analyzed data. BAP supervised the HTS work. JDH designed and performed experiments, analyzed data, supervised the remainder of the work, and wrote the manuscript. All the authors contributed to editing and accepted the final draft.

## Supplementary Material

Supplemental data

Unedited blot and gel images

Supporting data values

## Figures and Tables

**Table 1:**
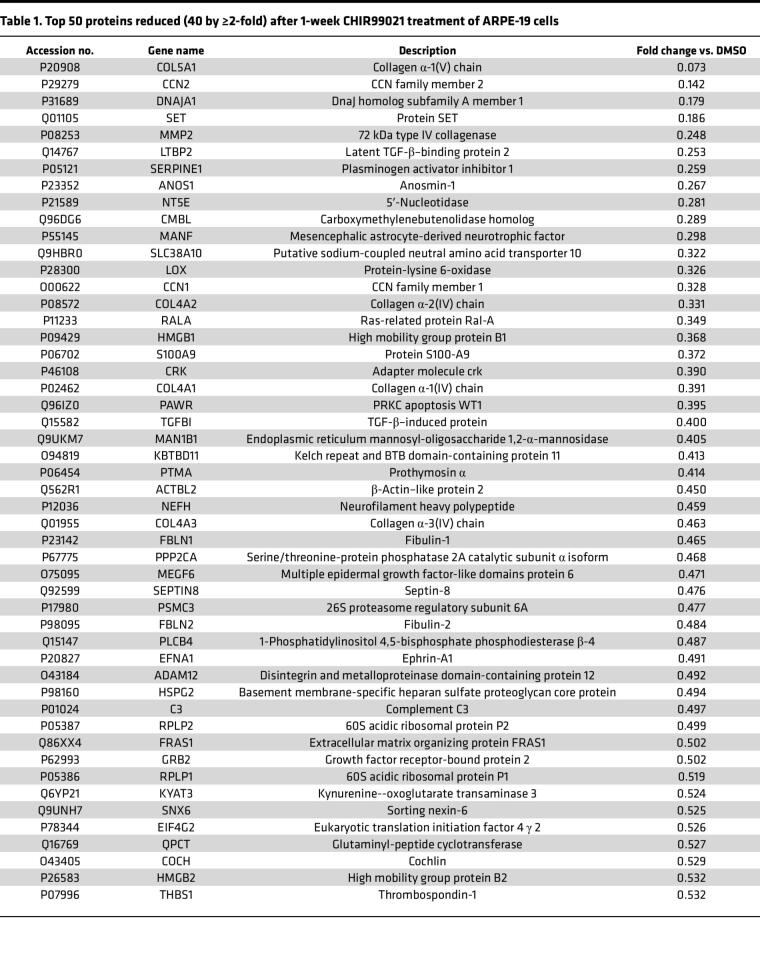
Top 50 proteins reduced (40 by ≥2-fold) after 1-week CHIR99021 treatment of ARPE-19 cells

**Table 2:**
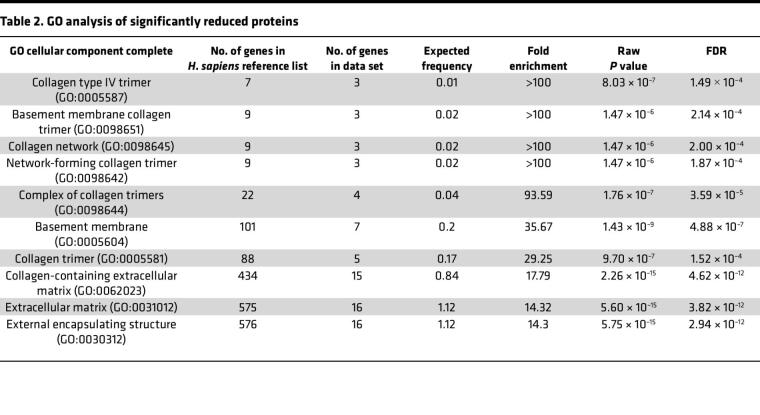
GO analysis of significantly reduced proteins

**Table 3:**
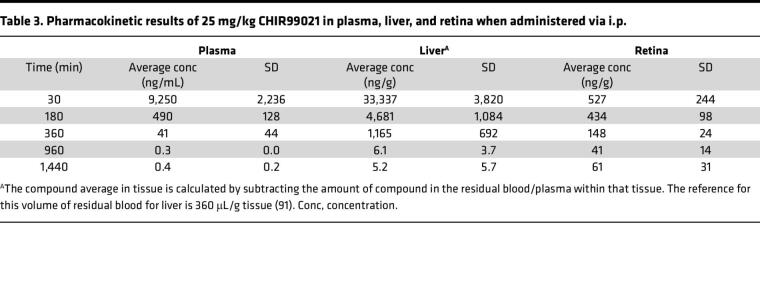
Pharmacokinetic results of 25 mg/kg CHIR99021 in plasma, liver, and retina when administered via i.p.

**Table 4:**
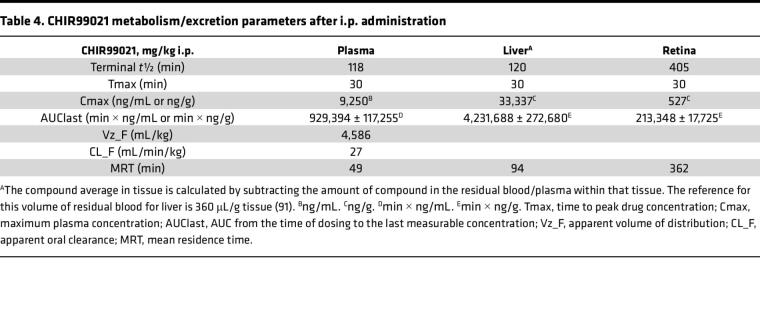
CHIR99021 metabolism/excretion parameters after i.p. administration

## Version 1 08/08/2024

Electronic publication
