# Supplementary material for: GSK3 inhibition reduces ECM production and prevents age-related macular degeneration–like pathology
Source: JCI Insight. 2024 Aug 8;9(15):e178050. doi: 10.1172/jci.insight.178050 (PMC11383595; doi:10.1172/jci.insight.178050)
Supplement: Supplemental data [file jciinsight-9-178050-s220.pdf]

SUPPLEMENTAL MATERIAL FOR:

GSK3 inhibition reduces ECM production and prevents age-related macular degeneration-like pathology

Sophia M. DiCesare[1], Antonio J. Ortega[2], Gracen E. Collier[1], Steffi Daniel[2], Krista N. Thompson[1], Melissa K. McCoy[3], Bruce A. Posner[3], and John D. Hulleman[2, Ω]

[1] Department of Ophthalmology, University of Texas Southwestern Medical Center, 5323 Harry Hines Blvd, Dallas, Texas, 75390, United States

[2] Department of Ophthalmology and Visual Neurosciences, University of Minnesota, 2001 6th St. SE, Minneapolis, Minnesota, 55455, United States

[3] Department of Biochemistry, University of Texas Southwestern Medical Center, 5323 Harry Hines Blvd, Dallas, Texas, United States

[Ω]Tel.: 1 612 624 8267

[Ω]Email: hulleman@umn.edu

The authors declare no conflicts of interest

SUPPLEMENTAL METHODS

Expanded Methods from Main Document

Cell culture. ARPE-19 cells (CRL-2302, ATCC) were cultured in DMEM/F12 (Corning) with 10% FBS (Omega Scientific) and 1% PSQ (ThermoFisher). Cells were kept at 37°C with 5% CO_2, and passages were performed every 3-4 days, or when confluent. For experiments, ARPE-19 cells were plated at confluence (unless otherwise noted), typically at a concentration of ~100,000 cells/cm^2, which equates to ~200,000 cells/mL. HEK-293T cells (Life Technologies) were cultured in DMEM High Glucose (4.5 g/L) supplemented with 10% FBS and 1% PSQ. Mouse NIH-3T3 fibroblasts (CRL-1658, ATCC) were maintained in DMEM high-glucose with 10% calf serum and 1% PSQ. Primary dermal fibroblasts (PCS-201-012, ATCC) were maintained in either low glucose DMEM with 10% FBS and 1% PSQ, or fibroblast growth media (Fibroblast Basal Medium [PCS-201-012, ATCC] supplemented with Low-Serum Fibroblast Growth Kit [PCS-201-041, ATCC]. Cells were periodically confirmed to be free of mycoplasma contamination using the Universal Mycoplasma Detection Kit (30-1012k, ATCC).

HiBiT F3 cell line generation. Low passage ARPE-19 cells were genomically edited to introduce a 2xFLAG-VS-HiBiT sequence immediately after the signal sequence cleavage site (Ser16) of F3 (Figure 1A). This area in F3 was specifically chosen because an extreme N-terminal tag would be cleaved off after co-translational import into the endoplasmic reticulum (ER), and because we have anecdotally noticed that C-terminal appendages (of any size) compromise the secretion efficiency of F3, in line with recent observations that a stop codon mutation in *EFEMP1* results in intracellular retention and causes a juvenile form of glaucoma (12). Introduction of the 29 amino acid 2xFLAG-VS-HiBiT tag at this position was not predicted to affect the native F3 signal sequence cleavage (Supplemental Figure 1, A and B), and FLAG tags have been used in a similar manner to force signal sequence cleavage immediately prior to the tag (85). For an in-depth

description of the editing process, please refer to a recent protocol paper (86). Briefly, a CRISPR/Cas9 ribonucleoprotein (RNP) was generated using Alt-R Sp. Cas9 Nuclease V3 (Integrated DNA Technologies, IDT) loaded with a crRNA/tracrRNA duplex (Supplemental Table 1). This RNP, combined with a single-stranded oligodeoxynucleotide (ssODN) repair template (Supplemental Table 1) were introduced into ARPE-19 cells using electroporation (1400 V, 20 ms, 2 pulses, Neon Transfection System, Life Technologies). After electroporation, cells were incubated in antibiotic-free media containing the DNA ligase IV inhibitor, SCR7 (1 μM, Sigma) for 48 h to promote homology-directed repair (HDR). Heterogenous cultures were expanded, assayed for secreted HiBiT via a luciferase assay and HiBiT blotting, and verified genomically for insertion of the 2xFLAG-VS-HiBiT sequence (Figure 1B, primers listed in Supplemental Table 2). Full, uncropped gel images are located in Supplemental Figure 12.

High-throughput screening (HTS). Heterogenous ARPE-19 edited cells were used for all screening experiments due to their growth properties in comparison to single colony clones, which generally had much slower growth, making them subpar for large-scale HTS purposes. HiBiT F3 ARPE-19 cells were seeded at a density of ~5,000 cells/well (in 30 μL with a MultiFlo, BioTek) in a white 384 well plate (781098, Greiner), and incubated at 37°C for 24 h. Media was exchanged using an EL406 microplate washer (BioTek). Compounds from the Prestwick Compound Library (1,200 compounds, Prestwick) and the National Institutes of Health clinical collection (446 compounds) were added using an Echo 655 (Beckman Coulter) at a final concentration of 5 μM and 0.1% DMSO. As a positive control for reduction of F3 secretion/production, brefeldin A (BFA, 50 μM) was used for the first column of each screening plate. Twenty-four hours after compound addition, plates were cooled to RT on the benchtop for 20 min followed by assaying for HiBiT signal in a whole well reading. LgBiT and lytic substrate (Promega) were added 1:100 and 1:50, respectively, into lytic buffer to generate a master mix. Fifteen microliters of the

lytic/LgBiT/substrate master mix was dispensed into each plate using a MultiFlo, shaken for 5 min, and luminescence was detected on an Envision plate reader (PerkinElmer).

HiBiT assay performance was calculated by determining the Z factor (Z') using the following equation:

$$Z' = 1 - \left(\frac{3 * (\sigma_{DMSO} + \sigma_{BFA})}{\mu_{DMSO} - \mu_{BFA}}\right)$$

Hit compounds were identified as having HiBiT F3 values less than three standard deviations from the mean of all compounds. A counter screen was used to identify toxic compounds that would be predicted to yield a false positive result (i.e., reduction in F3 signal due to cell death). Cells were plated using the same methods as the initial screen. Cherry-picked hit compounds were added the following day at the standard screening concentration (5 µM) and an additional dose that was 0.5 log below the screening dose. The Cell Titer Glo 2.0 (Promega) viability assay was performed after 24 h of treatment. A whole-well analysis was completed by adding 10 µL of Cell Titer Glo diluted 1:1 with PBS + 0.1% TX-100 to the plate and shaking for 5 min. Luminescence was again read on the Envision plate reader (PerkinElmer).

In addition to the cell viability counter screen, media alone was treated with the hit compounds to identify any false reductions in luminescent signal caused by NanoBiT luciferase inhibition. Briefly, conditioned media was collected from ARPE-19 HiBiT F3 cells, spun, and plated into 384-well plates. Hit compounds were added in a dose-response format and incubated for 24 h. Lytic buffer/LgBiT/substrate master mix was added to the plates, shaken for 5 min, then read on an Envision plate reader. Wells that had significantly lower amounts of HiBiT signal were noted and the corresponding compounds were removed from subsequent experiments. Remaining hit compounds were verified in a confirmatory screen using fresh compound from a new source plate.

Additional secondary metrics verified the effect of a hit compound in multiple assays and in different cell lines.

Short interfering RNA (siRNA). siRNA knockdown was used to verify the specificity of CRISPR HiBiT editing of F3 or fibulin-5 (F5). siRNAs (Silencer Select, Ambion, Supplemental Table 3) were introduced into cells containing HiBiT-tagged human F3, mouse F3, or human F5 by reverse transfection. For a 24 well plate, 2.14 μL of DharmaFECT4 (Horizon) was diluted into 250 μL OptiMEM (Thermo Fisher Scientific). siRNAs were added at a concentration of 100 nM. All samples were vortexed for 15 sec, then incubated at RT for 20 min. During this incubation period, ARPE-19 cells were trypsinized and normalized to a density of 466,000 cells/mL in full DMEM/F12 media. Two-hundred and fifty microliters of the siRNA/OptiMEM/DharmaFECT4 complex was added to a 24-well plate, after which an equivalent volume of cell suspension was added, yielding a final siRNA concentration of 50 nM. Plates were rocked to ensure cell distribution and to prevent clumping. After 24 h, the media was changed with full DMEM/F12. Seventy-two hours post knockdown, the media was changed again, and the HiBiT levels were determined the next day (96 h post knockdown) via an extracellular and intracellular HiBiT assay.

Non-HTS HiBiT assay. Cells were plated at a high density of 200,000 cells/mL for all non-HTS HiBiT assay experiments. The following day, media containing the indicted compound was added and incubated for either 72 h or 1 week. For one-week treatment wells, media was replaced after 96 h with fresh media containing compound. After treatment, extracellular and intracellular assays were performed to determine levels of HiBiT-tagged F3 using either the Nano-Glo HiBiT Extracellular Detection System or the Nano-Glo HiBiT Lytic Detection System (Promega). For the extracellular HiBiT protocol, 25 µL of the conditioned media was reacted with an equal volume of HiBiT master mix (extracellular or lytic buffer, 1:100 LgBiT, and 1:50 extracellular or lytic substrate) in a black-bottomed 96-well plate for 5 min with shaking. For intracellular HiBiT

detection, cells were washed in well with HBSS (Gibco), followed by addition of 50 µL of lytic HiBiT master mix (described above) and 5 min shaking. Forty microliters of the lysate was transferred to a black 96-well plate for a luminescent reading on a Synergy 2 (BioTek) or a GloMax (Promega).

<u>Western blotting.</u> Cells for western blotting were rinsed with HBSS followed by lysis in buffer containing radioimmunoprecipitation buffer (RIPA, Santa Cruz), protease inhibitor (Pierce) and benzonase (Millipore Sigma), or the Nano-Go HiBiT Lytic Detection System. Lysis buffer was added to each well at a volume of 50 µL (for a 24 well plate, for example) followed by shaking for 5-15 min, and lysate was collected into Eppendorf tubes on ice. The samples were spun at 4°C for 10 min at 21,000 x g, and the soluble supernatant was collected. For cells lysed in RIPA buffer, a bicinchoninic acid assay (BCA, Pierce Thermo Scientific) was used to quantify protein levels and normalize them to 20 µg. For cells lysed in HiBiT Lytic buffer, samples were normalized by volume due to the presence of DTT, which interferes with the BCA assay. Samples were boiled in 1x reducing Laemmli buffer for 5 min, then loaded onto a 4-20% Tris-Gly SDS-PAGE gel and run at 140 V for 80 min. Proteins were transferred to 0.2 µm nitrocellulose membrane using a semidry apparatus (P0 protocol, iBlot2, Life Technologies). Total transferred protein was visualized with Ponceau S (Sigma-Aldrich) and incubated overnight in blocking buffer (Intercept Blocking Buffer, LI-COR). The following day, blots were incubated for 1 h at RT with primary antibody [GSK3α (sc-5264, Santa Cruz), GSK3β (9315S, Cell Signaling), or GAPDH (sc-47724, Santa Cruz)] diluted in 5% bovine serum albumin (BSA) in Tris-buffered saline (TBS) and 0.05% NaN_3. Blots were rinsed in TBS with 0.05% Tween (TBS-T) and incubated for 40 min at RT in an appropriate anti-mouse or anti-rabbit secondary antibody (1:10,000-15,000, LI-COR) dissolved in 5% milk in TBS-T. Blots were again washed in TBS-T, then TBS, followed by water, and imaged

on an Odyssey CLx (LI-COR), followed by analysis using the corresponding ImageStudio Software (LI-COR). Full, uncropped blot images are located in Supplemental Figure 12.

HiBiT blotting. Samples were lysed, prepared, and separated by SDS-PAGE as described above for western blotting. Normalized protein was reduced, denatured, and run through an SDS-PAGE gel, followed by transfer to a nitrocellulose membrane (P0 program, iBlot2). Once transferred, the membrane was incubated in TBS-T (10 min to 1 h, RT) to expose the HiBiT epitope. Blots were then incubated with blotting buffer containing 1:200 LgBiT for 1-2 h with rocking at RT (N2410, Promega). Next, NanoGlo substrate was added (1:500) and the blots were incubated for 5-10 min at RT. The resulting chemiluminescence signal was imaged on an Odyssey Fc (LI-COR). Full, uncropped blot images are located in Supplemental Figure 12.

Lactate dehydrogenase (LDH) release assay. Cells were treated with CHIR for either 72 h or 1 week. Media was then collected and spun at 1,000 RPM (100 x g) for 5 min to remove potential floating cells. Fifty microliters (technical triplicates) of cleared media was mixed with an equal amount of LDH reaction buffer (LDH Cytotoxicity Assay Kit, Pierce) in a clear 96-well plate for 30 min at 37°C. A DMSO-treated media sample served as a control while cells treated with cell lysis buffer (provided in the LDH Cytotoxicity Kit) served as a positive control. Fifty microliters of stopping solution was added to each well and the absorbance at 490 nm was read on a GloMax plate reader(Promega).

7xTCF-eGFP mCherry (7TGC) and 7xTCF-firefly luciferase puromycin (7TFP) lentivirus production. VSV-g-pseudotyped replication incompetent lentivirus was generated as we have described previously (88, 89). Briefly, lentiviral plasmids (Addgene plasmids #24304 and #24308, kind gifts of Roel Nusse (42)) were co-transfected with psPAX2 and VSV-g plasmids (Addgene plasmids #12260 and #12259, gifts of Didier Trono) into low passage HEK-293T cells plated at

$1x10^6$ cells/well of a poly-D-lysine-coated 6-well plate using Lipofectamine 3000 (Life Technologies). The next day, media was discarded and replaced with fresh media. Media collected 24 h and 48 h later was pooled and filtered through a 0.45 μm filter. Lenti-X GoStix (Takeda) were used to confirm the production of virus. To establish 7TGC and 7TFP cell lines, HiBiT F3 ARPE-19 cells were plated at $1x10^6$ cells/well of a 6 well plate and infected with lentivirus in full media containing polybrene for 24 h. 7TGC cells were not put under selective pressure whereas 7TFP cells were then selected with 1 μg/mL of puromycin for 1-2 weeks.

Firefly luciferase (FLuc) assay. The HiBiT F3 7TFP ARPE-19 cells were treated with CHIR for 1 week (with a 72 h media change) and assayed for FLuc expression. Briefly, after a total of 1 week of treatment, media was aspirated and cells were washed with HBSS. Fifty microliters lysis buffer (Firefly Luciferase Glow Assay, Pierce) was added to each well, then the plate was rocked for 15 min. To monitor FLuc activity, 50 µL of working solution containing luciferin and Firefly Glow Assay Buffer was added to a black 96-well plate. Ten microliters of the lysate was added to respective wells, then read on a Synergy2 plate reader.

MMP2 zymography. HiBiT WT F3 or HiBiT R345W F3 ARPE-19 cells were plated at a density of ~100,000 cells per well of a 12-well, or ~30,000 cells per well of a 24-well, 0.4 μm polyester transwell plate (Corning), and media was changed to serum free media the following day and every 3-4 days thereafter. After 1 week growing on transwells, cells were treated with either DMSO (0.1%) or 1 μM CHIR in serum free media. After 2 weeks on transwells (1 week of treatment), an extracellular HiBiT assay and MMP2 zymography were performed. Briefly, for zymography, media from the apical and basal chamber was collected and combined with non-reducing SDS buffer followed by running on a 10% gelatin gel (Novex) for 90 minutes at 140 V. MMP2 was renatured in buffer (G-biosciences) for 30 min at RT. Gels were then changed to

developing buffer (G-Biosciences) for an additional 30 min at RT. After changing to fresh developing buffer, gels were gently shaken overnight at 37°C. The following day, the gels were stained in Coomassie R-250 for 1 h, then destained and imaged on an Odyssey Clx (LI-COR). Bands were quantified using Image Studio software (LI-COR). Full, uncropped gel images are located in Supplemental Figure 12.

Secreted proteome visualization and mass spectrometry. HiBiT ARPE-19 cells were treated with CHIR for 72 h in serum-free DMEM/F12 media. To visualize separated total protein, conditioned media was concentrated (Amicon Ultra 0.5 mL centrifuge unit, 3,000 MWCO, Millipore Sigma) and run on a 4-20% Tris-Gly SDS-PAGE gel for 80 min at 140 V. Protein bands were imaged by silver staining (SilverQuest Silver Staining Kit, Invitrogen). In parallel, to prepare samples for mass spectrometry, an aliquot of the same concentrated media sample was run for 10 min at 140 V on a 4-20% Tris-Gly SDS-PAGE gel, stained with Coomassie Blue, and the single band corresponding to total secreted protein was excised and submitted for mass spectrometry to the UT Southwestern Proteomics Core. Briefly, samples were digested overnight with trypsin (Pierce) following reduction and alkylation with DTT and iodoacetamide (Sigma Aldrich). The samples then underwent solid-phase extraction cleanup with an Oasis HLB plate (Waters) and 2 μL of each sample was injected onto a QExactive HF mass spectrometer (Thermo Fisher Scientific) coupled to an Ultimate 3000 RSLC-Nano liquid chromatography system (Thermo Fisher Scientific). Samples were injected onto a 75 μm i.d., 15-cm long EasySpray column (Thermo) and eluted with a gradient from 0-28% buffer B over 90 min with a flow rate of 250 nL/min. Buffer A contained 2% (v/v) ACN and 0.1% formic acid in water, and buffer B contained 80% (v/v) ACN, 10% (v/v) trifluoroethanol, and 0.1% formic acid in water. The mass spectrometer operated in positive ion mode with a source voltage of 2.5 kV and an ion transfer tube temperature of 275°C. MS scans were acquired at 120,000 resolution in the Orbitrap and up to 20 MS/MS spectra were obtained

for each full spectrum acquired using higher-energy collisional dissociation (HCD) for ions with charges 2-8. Dynamic exclusion was set for 20 s after an ion was selected for fragmentation. Raw MS data files were analyzed using Proteome Discoverer v. 2.4 SP1 (Thermo Fisher Scientific), with peptide identification performed using Sequest HT searching against the human protein database from UniProt (https://www.uniprot.org/). Fragment and precursor tolerances of 10 ppm and 0.6 Da were specified, and three missed cleavages were allowed. Carbamidomethylation of Cys was set as a fixed peptide modification, with oxidation of Met set as a peptide variable modification. The false-discovery rate (FDR) cutoff was 1% for all peptides.

Sample preparation for RNAseq. HiBiT F3 ARPE-19 cells were plated at 200,000 cells/mL in DMEM/F12 media with 10% FBS and 1% PSQ. The following day, media containing DMSO (1:1000) or 1 μM CHIR was added to the cells. Cells were treated for 96 h before an additional media change. Seven days after beginning treatment, RNA from vehicle (DMSO)-treated and 1 μM CHIR-treated cells was extracted using an Aurum Total RNA Kit (BioRad) and stored at -80°C. Fifteen microliters of sample was provided to Novogene. A total amount of 1 μg RNA per sample was used as input material for the RNA sample preparations. Sequencing libraries were generated using NEBNext UltraTM RNA Library Prep Kit for Illumina (NEB) following manufacturer's recommendations and index codes were added to attribute sequences to each sample. Briefly, mRNA was purified from total RNA using poly-T oligo-attached magnetic beads. Fragmentation was carried out using divalent cations under elevated temperature in NEBNext First Strand Synthesis Reaction Buffer (5X). First strand cDNA was synthesized using random hexamer primer and M-MuLV Reverse Transcriptase (RNase H). Second strand cDNA synthesis was subsequently performed using DNA Polymerase I and RNase H. Remaining overhangs were converted into blunt ends via exonuclease/polymerase activities. After adenylation of 3' ends of DNA fragments, NEBNext Adaptor with hairpin loop structure were ligated to prepare for

hybridization. In order to select cDNA fragments of preferentially 150~200 bp in length, the library fragments were purified with AMPure XP system (Beckman Coulter). Then 3 μL USER Enzyme (NEB) was used with size-selected, adaptor ligated cDNA at 37 °C for 15 min followed by 5 min at 95 °C before PCR. Then PCR was performed with Phusion High-Fidelity DNA polymerase, Universal PCR primers and Index (X) Primer. At last, PCR products were purified (AMPure XP system) and library quality was assessed on the Agilent Bioanalyzer 2100 system.

CHIR PK experiment. C57BL/6J (originally from Jackson Laboratories) were dosed via intraperitoneal injection with CHIR trihydrochloride (Tocris) dissolved in 2% DMSO with 5.7% Captisol (Ligand Pharmaceuticals) in 1x PBS at a concentration of 5 mg/mL. Mice were dosed at 25 mg/kg (average weight of 24.7 g). The dosing time points were 30, 180, 360, 960, and 1440 min, with a 0-min control group. Each timepoint included three mice, with at least one male and one female in each group. At select time points, isoflurane was used to anesthetize mice prior to tissue collection. Plasma, liver, and neural retina (both eyes pooled) were collected, and the samples were flash frozen in liquid nitrogen. All samples were then sent to the UT Southwestern Preclinical Pharmacology Core and the amount of CHIR was measured in each tissue using LC-MS/MS (see below).

LC-MS/MS analysis of CHIR: Retina tissue, liver tissue and plasma were analyzed for CHIR concentrations using an LC-MS/MS method. Retinas and livers were homogenized in PBS. Retina homogenates were made using BeadBug prefilled tubes with 3.0 mm Zirconium beads (Z763802, Sigma) and a BeadBug microtube homogenizer run for 1 min at 2800 RPM (800 x g). For standards, blank commercial plasma (Bioreclamation) or untreated liver or brain tissue homogenate was spiked with varying concentrations of compound. Standards and samples were mixed with three-fold volume of 100% acetonitrile containing 0.133% formic acid and 33.3 ng internal standard (tolbutamide, Sigma), vortexed, and then spun 5 min at 16,100 x g. Supernatant

was removed and spun again and the resulting second supernatant was put into an HPLC 96-well plate and analyzed by LC-MS/MS using a Sciex Triple Quad™ 4500 mass spectrometer coupled to a Shimadzu Prominence LC. CHIR was detected with the mass spectrometer in MRM (multiple reaction monitoring) mode by following the precursor to fragment ion transition 465 → 146.1. An Agilent Zorbax XDB-C18 column (50 x 4.6mm, 5 μm packing) was used for chromatography with the following conditions: Buffer A: dH20 + 0.1% formic acid, Buffer B: MeOH + 0.1% formic acid, 1.5mL/min flow rate, 0-1.5 min 3%B, 1.5-2.0 min gradient to 100%B, 2.0-3.5 min 100%B, 3.5-3.6 min gradient to 3%B, 3.6-4.5 3%B. Tolbutamide (transition 271.2 → 91.2) was used as an internal standard. Back-calculation of standard curve and quality control samples were accurate to within 15% for 85-100% of these samples at concentrations ranging from 0.5 ng/ml to 10000 ng/ml.

Additional Methods (appearing only in Supplemental Material)

Porcine retinal pigment epithelium (RPE) culture. To assess the long-term effects of CHIR on RPE tight junction formation and maintenance, primary porcine RPE cultures were used. Briefly, procured pig eyes were processed according to Toops et al 2014 Exp. Eye Res. with slight modification. Posterior eye cups were dissected and RPE were isolated by trypsinization followed by expansion in T25 culture dishes (5 eyes per dish) for 2 weeks in differentiation media (with 10 mM nicotinamide). Cells were then trypsinized and seeded on transwells at a density of 300,000 cells/cm^2 on collagen-coated (5 μg/cm^2) 0.4 μm transwells (Corning). Cells were allowed to differentiate for 2 additional weeks prior to treatment with DMSO (1:1,000) or CHIR (1 μM). Cells were then washed 3x with HBSS, fixed in 4% PFA (30 min, RT), washed again with HBSS, and stained with CytoPainter phalloidin-iFluor 647 (Abcam, 1:250 in PBS, 1% BSA, 0.5% Triton-X-100, 0.5% Tween-20, 1 h, RT). Transwells were washed again, excised and mounted using Fluoromount G (ThermoFisher) on microscope slides and imaged on a Keyence BZ-X810 at 40x magnification.

HiBiT mouse F3 cell line generation. To add HiBiT to mouse F3, NIH-3T3 fibroblasts (ATCC CRL-1658) were used. Similar to the procedure described above, a 2xFLAG VS HiBiT sequence was inserted immediately after the Ser17 residue of mouse F3 (Supplemental Figure 5, A and B). Unlike in *EFEMP1*, no mutation of the PAM site was required in *Efemp1* due to significant disruption of the gRNA sequence after HiBiT insertion (Supplemental Figure 6A). RNP, ssODN, and electroporation enhancer were introduced into the NIH-3T3 cells (1400 V, 20 ms, 2 pulses, Neon Transfection System). After electroporation, cells were incubated in antibiotic-free media containing HDR Enhancer V3 (1 μM, IDT) for 48 h to promote homology-directed repair. Heterogenous cultures were expanded and assayed for secreted HiBiT via a luciferase assay.

HiBiT fibulin-5 (F5) cell line generation. As a control cell line for HiBiT F3 cells, we appended HiBiT onto endogenous fibulin-5 (FBLN5, DANCE, or F5), a highly homologous protein to F3 (19), in a similar manner, immediately after the F5 signal sequence cleavage after residue Ala23 (Supplemental Figure 8A). No mutation of the PAM site was required in *FBLN5* due to significant disruption of the gRNA sequence after HiBiT insertion (Table S1). RNP, ssODN, and electroporation enhancer were introduced into ARPE-19 cells using electroporation (1400V, 20 ms, 2 pulses, Neon Transfection System). After electroporation, cells were incubated in antibiotic-free media containing HDR Enhancer V3 (1 μM, IDT) for 48 h to promote homology-directed repair. Heterogenous cultures were expanded and assayed for secreted HiBiT via a luciferase assay.

SUPPLEMENTAL FIGURE LEGENDS

Supplemental Figure 1: Introduction of the 2xFLAG-VS-HiBiT tag is not predicted to alter human F3 signal sequence cleavage. (A) SignalP5.0 predicted signal sequence cleavage of human wild-type (WT) F3 and (B) human F3 after introduction of the 2xFLAG-VS-HiBiT tag.

Supplemental Figure 2: Example data from HTS plate indicating assay uniformity and identification of potential hit compounds. (A) Example plate data from a whole-well HiBiT assay mock screen performed with DMSO and BFA (as a positive control) to identify an assay Z'-score. (B, C) Representative screening data identifying a potential F3 reducer (B) or a potential F3 enhancer (C). (D, E) Linearity of the HiBiT luminescence assay. Conditioned media from WT HiBiT F3 cells was diluted over a range of concentrations followed by a HiBiT assay. Regression analysis shows excellent linearity over 6 logs of dilution. n = technical triplicates performed in two independent experiments for D, E.

Supplemental Figure 3: Select hit compounds identified in the primary screen were confirmed in dose-response. (A) Seven hit compounds were tested at 0.2 nM – 50 μM for 24 h. All hits reproduced and demonstrated varying levels of dose-responsiveness. (B) Table of 24 h, whole-well EC_{50} values obtained from panel A.

Supplemental Figure 4: Verification of CHIR99021 activity toward F3 in a human non-RPE cell line and primary porcine RPE. (A) F3 in primary human dermal fibroblasts was edited with a HiBiT tag followed by treatment with CHIR99021 for 72 h. $n \geq 3$ independent experiments with mean of each experiment presented as a single data point in this graph. ** $p < 0.01$, one sample t-test vs. hypothetical mean of 1 (i.e., unchanged). (B,C) Primary porcine RPE grown on

transwells were treated with DMSO (1:1,000) or CHIR (1 μM) for 11 weeks followed by staining for phalloidin (F-actin) to delineate RPE cell borders. Scale bar = 100 μm.

Supplemental Figure 5: Introduction of the 2xFLAG-VS-HiBiT tag is not predicted to alter mouse F3 signal sequence cleavage. (A) SignalP5.0 predicted signal sequence cleavage of mouse (mus) wild-type (WT) F3 and (B) human F3 after introduction of the 2xFLAG-VS-HiBiT tag.

Supplemental Figure 6: HiBiT editing of mouse fibroblast NIH-3T3 cells followed by treatment with CHIR99021 demonstrates cross species activity of the compound. (A) Schematic demonstrating the 2xFLAG-VS-HiBiT F3 design for genomic insertion into the mouse genome. The upward arrow indicates the predicted signal sequence cleavage site based on SignalP5.0 prediction. (B) The origins of the HiBiT signal were confirmed to be from mouse F3 as demonstrated by siRNA knockdown experiments. n = 3 independent experiments, **** $p < 0.0001$, t-test vs. non-targeting siRNA. (C) Seventy-two hour treatment with CHIR99021 reduces F3 production in mouse cells. n = 3 independent experiments, ** $p < 0.01$, *** $p < 0.001$, **** $p < 0.0001$, one-way ANOVA with Dunnett's multiple comparison test vs. vehicle (DMSO) treated samples.

Supplemental Figure 7: Introduction of the 2xFLAG-VS-HiBiT tag is not predicted to alter human fibulin-5 (F5) signal sequence cleavage. (A) SignalP5.0 predicted signal sequence cleavage of human wild-type (WT) F5 and (B) human F5 after introduction of the 2xFLAG-VS-HiBiT tag.

Supplemental Figure 8: Production of HiBiT-tagged human F5 is also reduced by CHIR99021 treatment. (A) Schematic demonstrating the design of 2xFLAG-VS-HiBiT genomic

insertion onto the N-terminus of human F5 in ARPE-19 cells. The origins of the resulting HiBiT signal were confirmed to be F5 gene expression-dependent using siRNA. Representative data of n = 3 independent experiments, average ± S.D. of technical triplicates. *** $p < 0.001$. (C) HiBiT F5-expressing ARPE-19 cells were treated with CHIR99021 for 72 h, followed by a HiBiT assay on conditioned media. n = 3 independent experiments performed in biological duplicates each time, * $p < 0.05$, **** $p < 0.0001$. (D) One-week CHIR99021 treatment also leads to reduced extracellular and intracellular F5 levels. n = 3 independent experiments performed in biological triplicate, ** $p < 0.01$, **** $p < 0.0001$. one-way ANOVA with Dunnett's multiple comparison test vs. non-targeting or vehicle (DMSO) treated samples for all panels.

Supplemental Figure 9: Low level, 1-week CHIR99021 treatment does not substantially affect the secreted proteome from ARPE-19 cells. Total concentrated secreted protein after 1 week of CHIR99021 treatment silver stained to visualize differences in overall protein abundance. Representative data of three independent experiments shown in biological duplicate.

Supplemental Figure 10: Design and validation of R345W$^{+/+}$ ARPE-19 knockin cells and their subsequent HiBiT editing. (A) Design of the R345W knockin strategy using CRISPR/Cas9 editing and homology-directed repair. (B) Genomic DNA from single colonies was isolated and validated to be homozygous ($^{+/+}$) for the R345W mutation. (C) The validated R345W$^{+/+}$ clone was then edited to include the 2xFLAG-VS-HiBiT tag.

Supplemental Figure 11: Not all TEM fields of view show BLamD. Some TEM fields were found to contain no BLamDs in both (A) vehicle (A) and CHIR99021-treated mice (B). Note the healthy basal infoldings in these images also. Scale bar = 2 μm.

Supplemental Figure 12: Full gel and blot images for the indicated figure.

Supplemental Table 1: CRISPR reagent sequences used for guide RNAs, PAM sites, and ssODN design for insertion of the 2xFLAG-VS-HiBiT tag.

Supplemental Table 2: Primer sequences for exon 2 amplification and gDNA verification of 2xFLAG-VS-HiBiT insertion into the F3 gene.

Supplemental Table 3: siRNA sense sequences used for confirming the origins of the HiBiT signal in generated cell lines.

Supplemental Table 4: Processed data received from HTS with eight hit compounds of interest and additional information found about MOA, SMILES, HiBiT readout, etc.

Supplemental Table 5: Top 50 increased proteins between DMSO and CHIR treated cells.

Supplemental Table 6: GO analysis of top proteins and their involvement in cellular composition.

Supplemental Table 7: RNA-seq genes identified to have ≥ 2-fold decrease between CHIR treated and DMSO treated cells.

Supplemental Table 8: RNA-seq genes identified to have ≥ 2-fold increase between CHIR treated and DMSO treated cells.

Supplemental Figure 1.

A

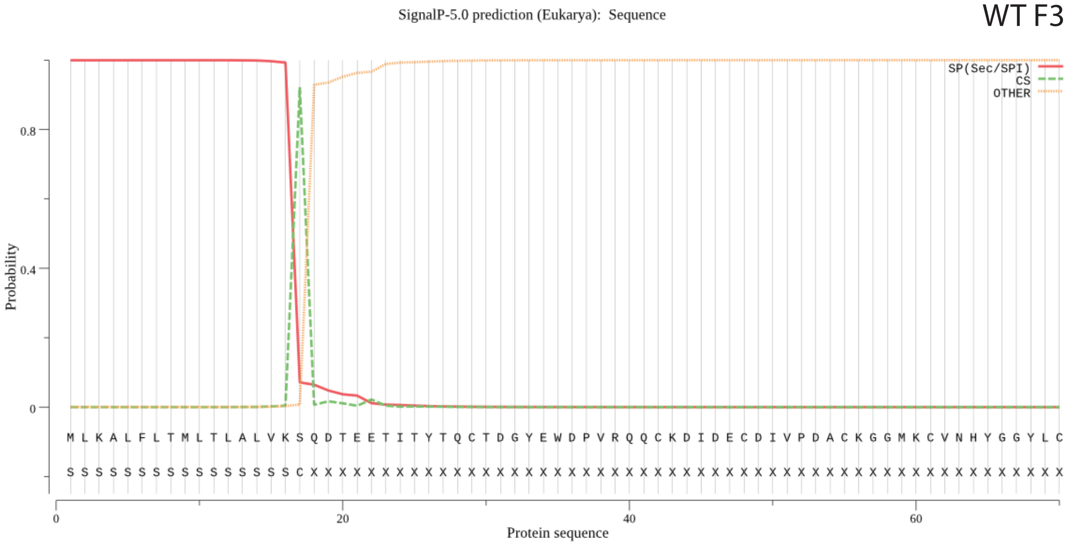

B

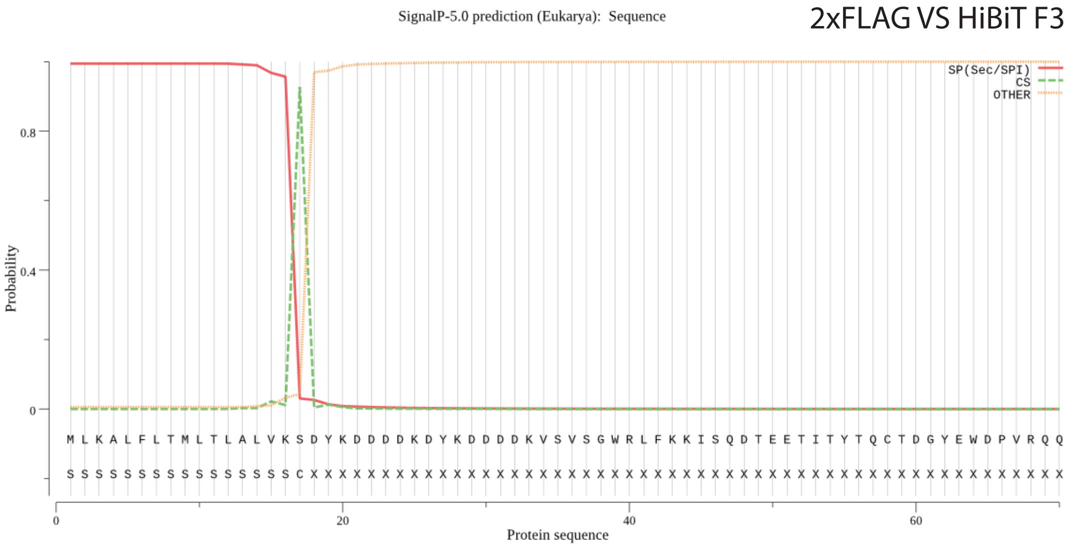

Supplemental Figure 2.

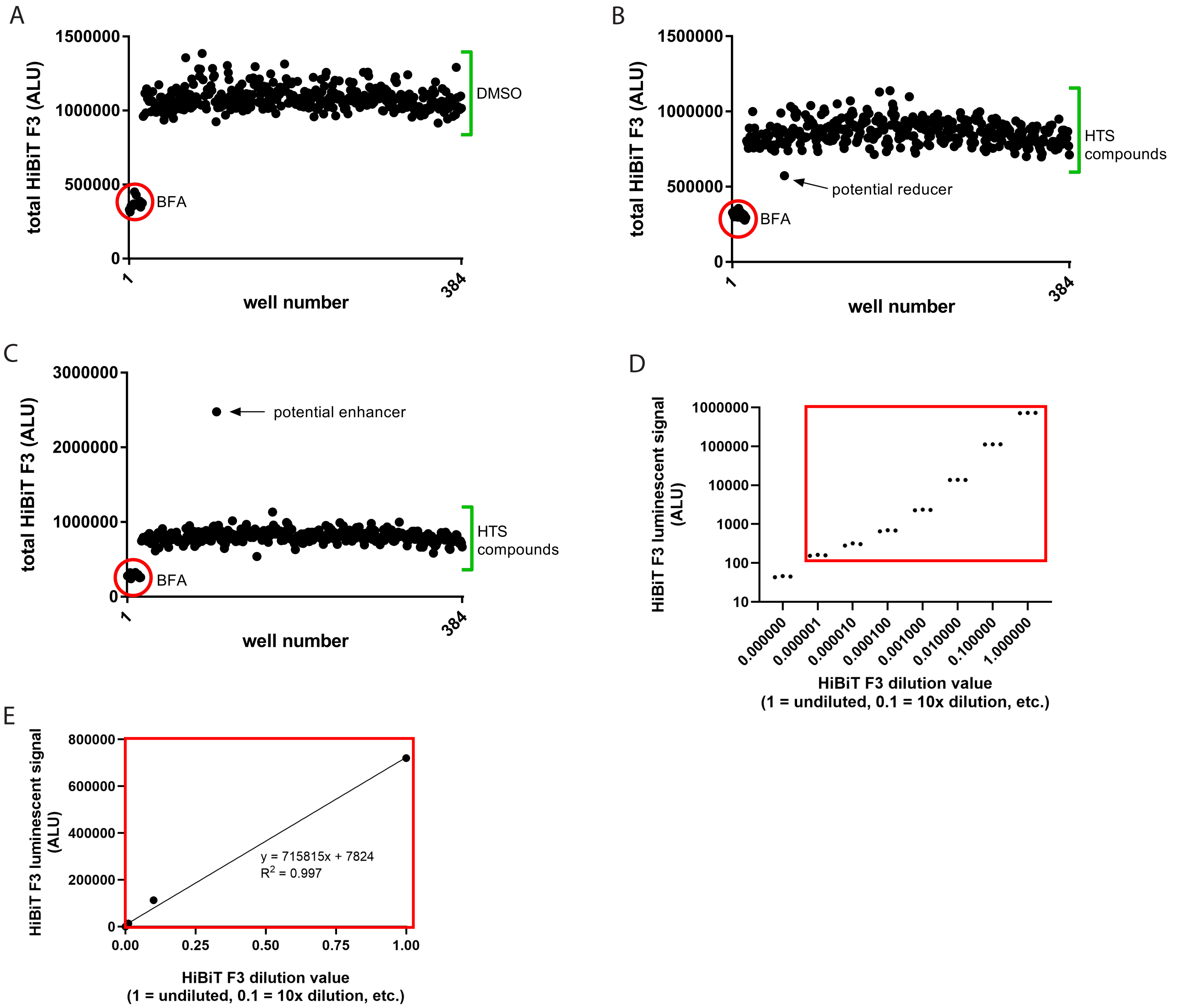

Supplemental Figure 3.

A

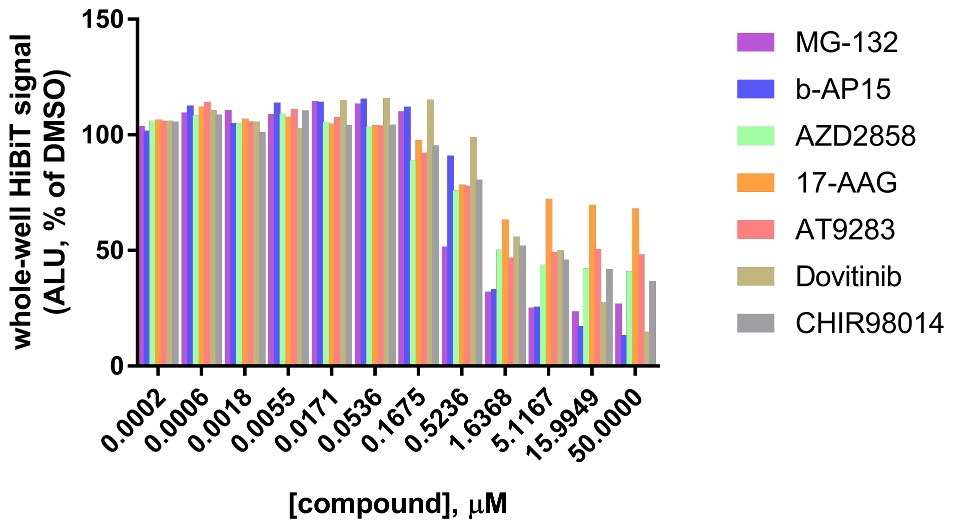

B

	whole well 24 h EC_{50}
**MG-132**	490.9 nM
**b-AP15**	1,120 nM
**AZD2858**	495.7 nM
**17-AAG**	264.6 nM
**AT9283**	393.7 nM
**Dovitinib**	2,212 nM
**CHIR98014**	721 nM

Supplemental Figure 4

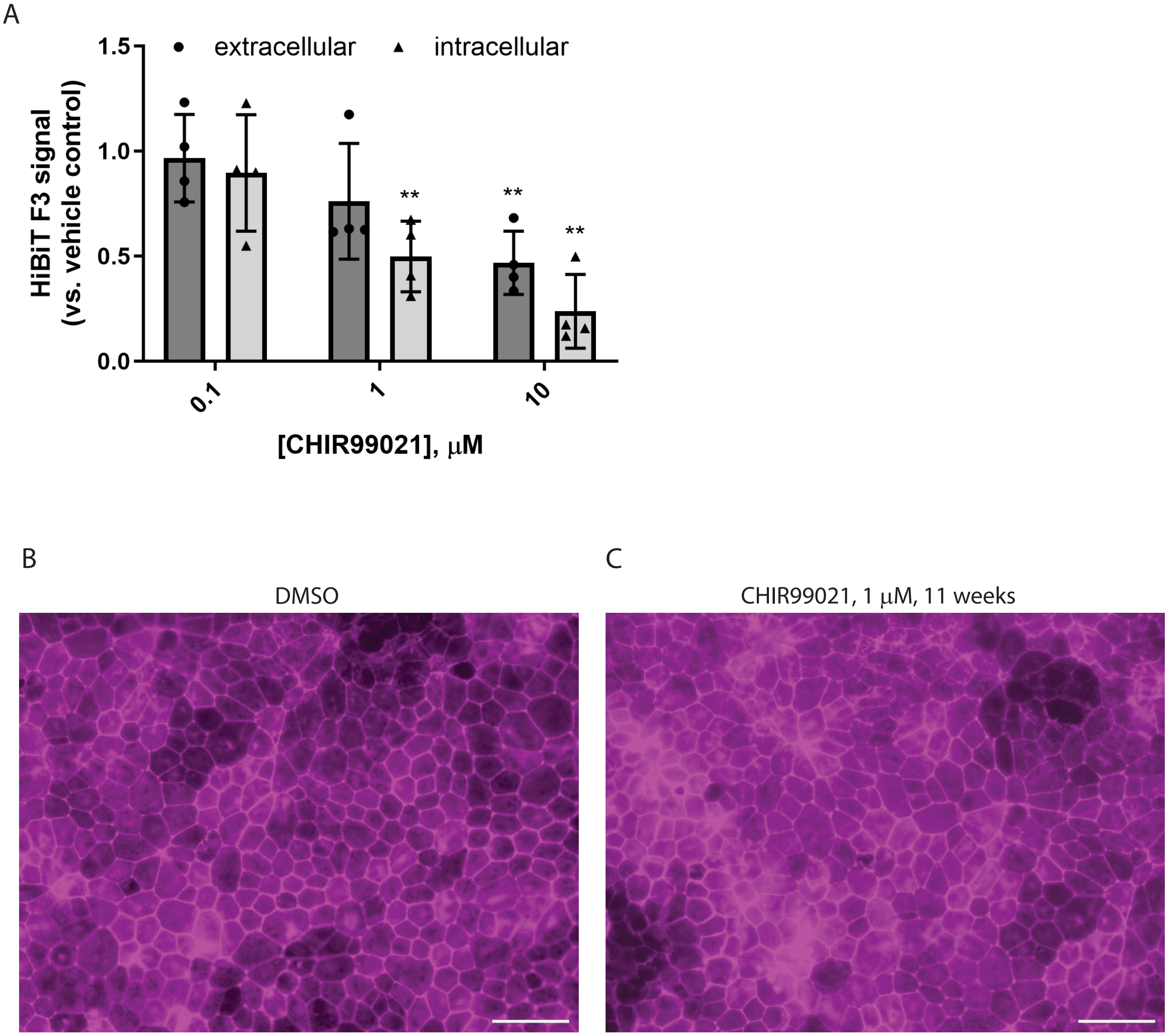

Supplemental Figure 5.

A

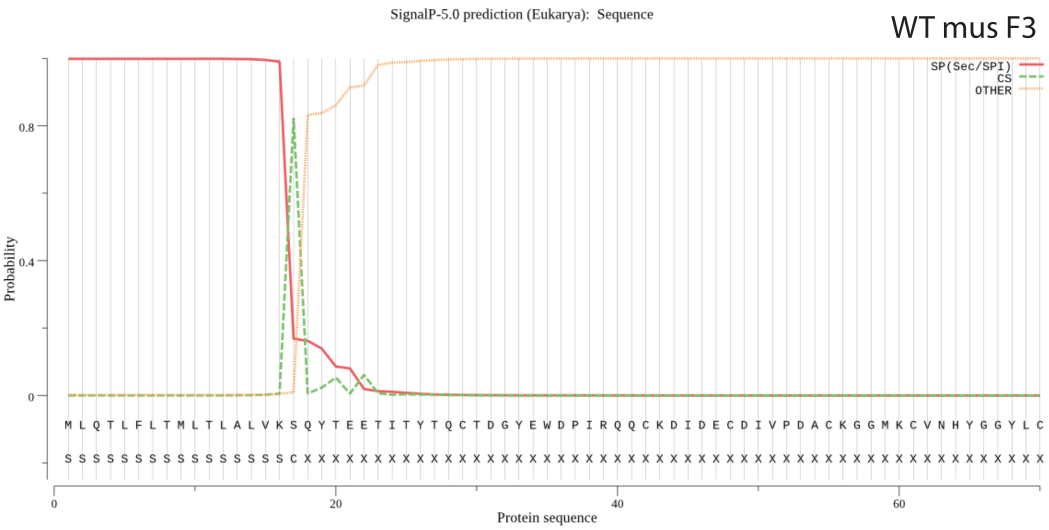

B

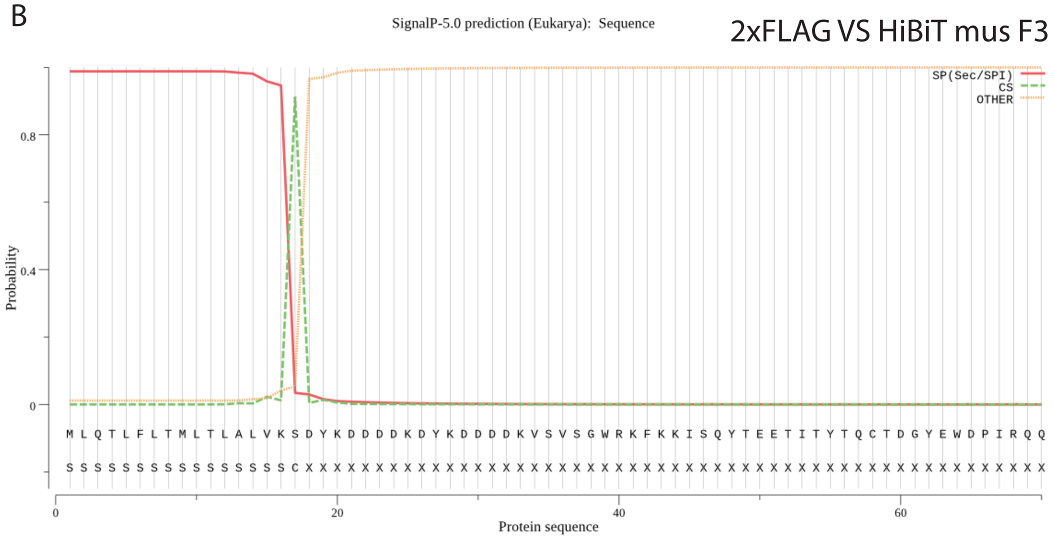

Supplemental Figure 6.

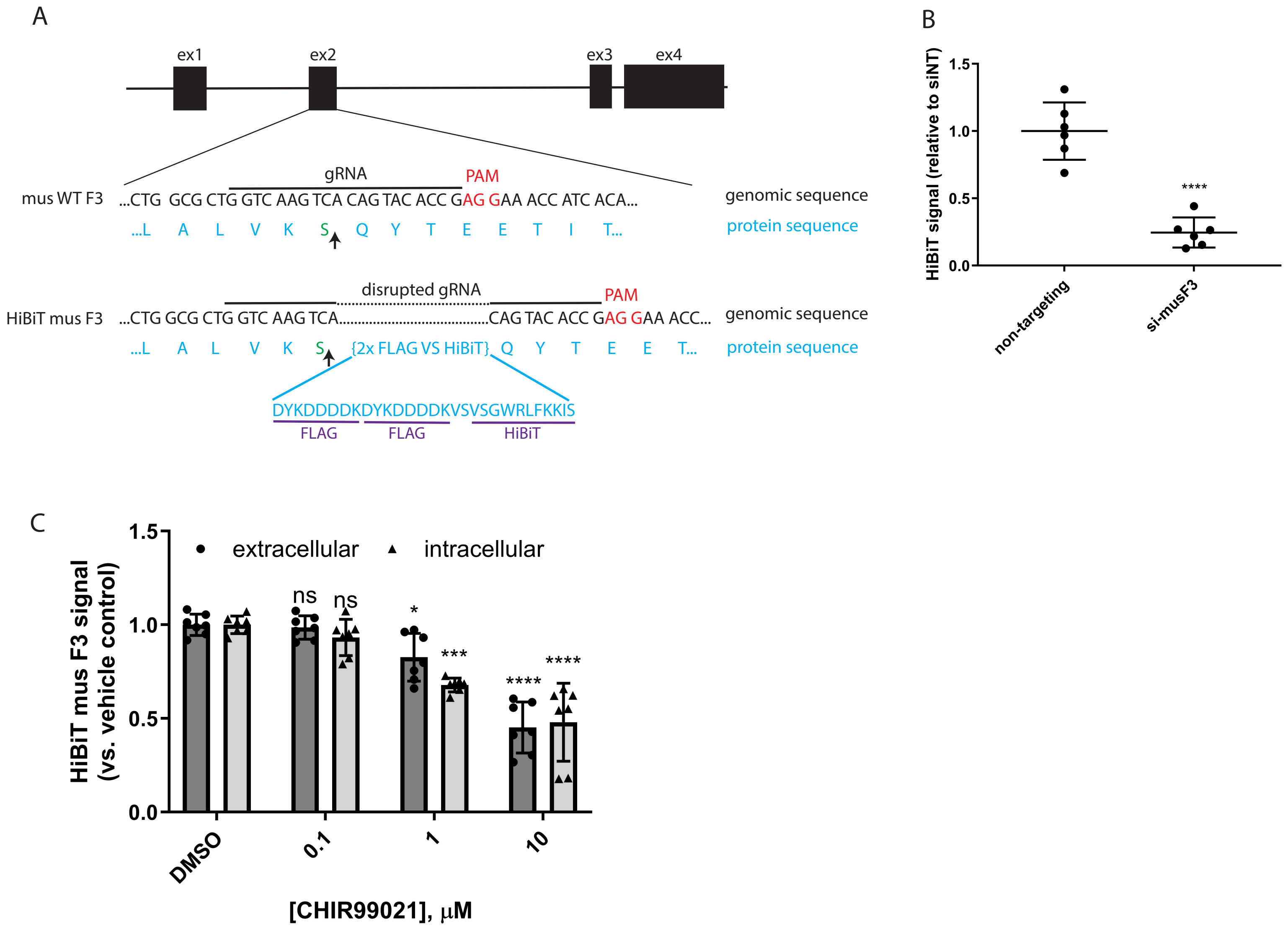

Supplemental Figure 7.

A

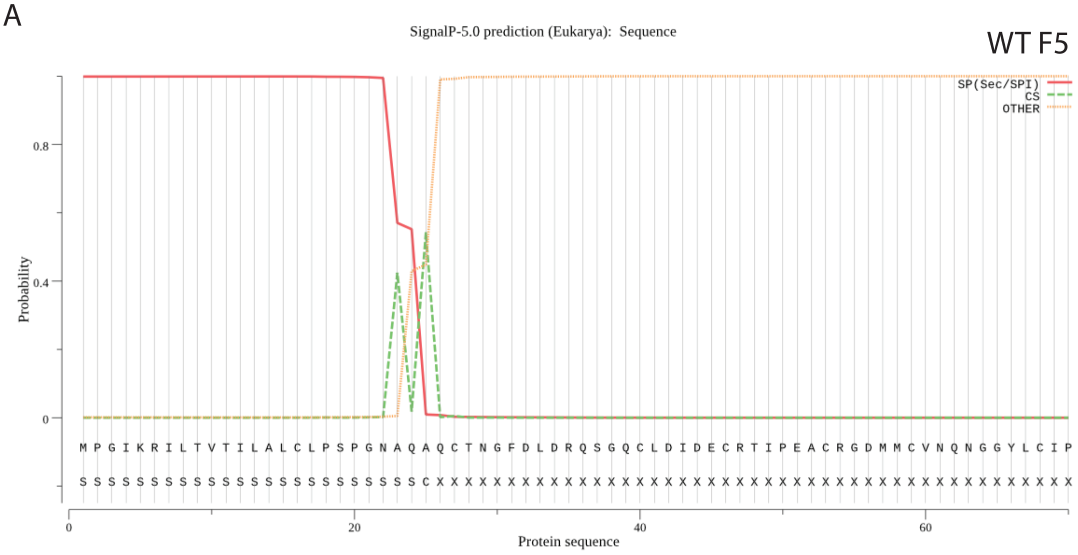

B

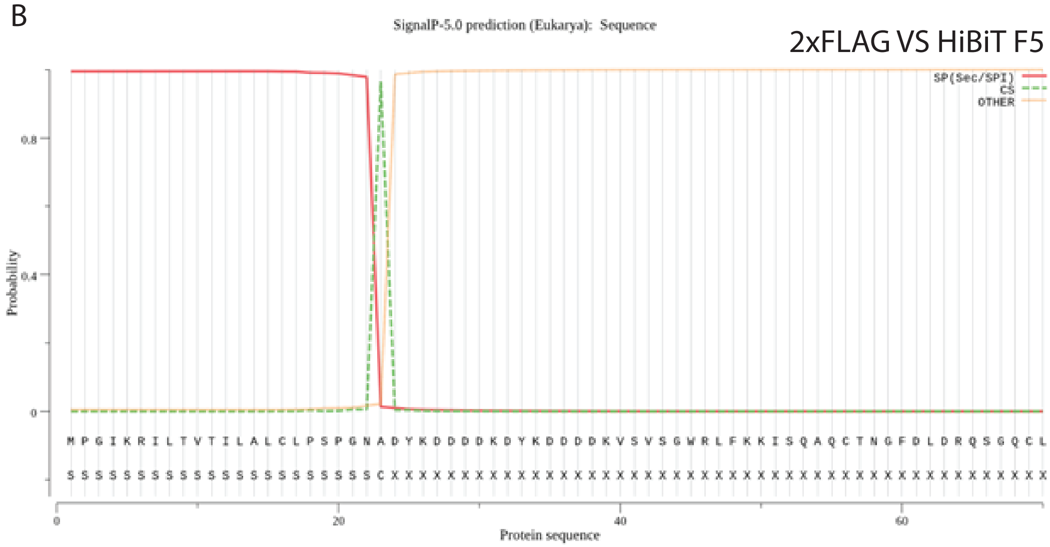

Supplemental Figure 8.

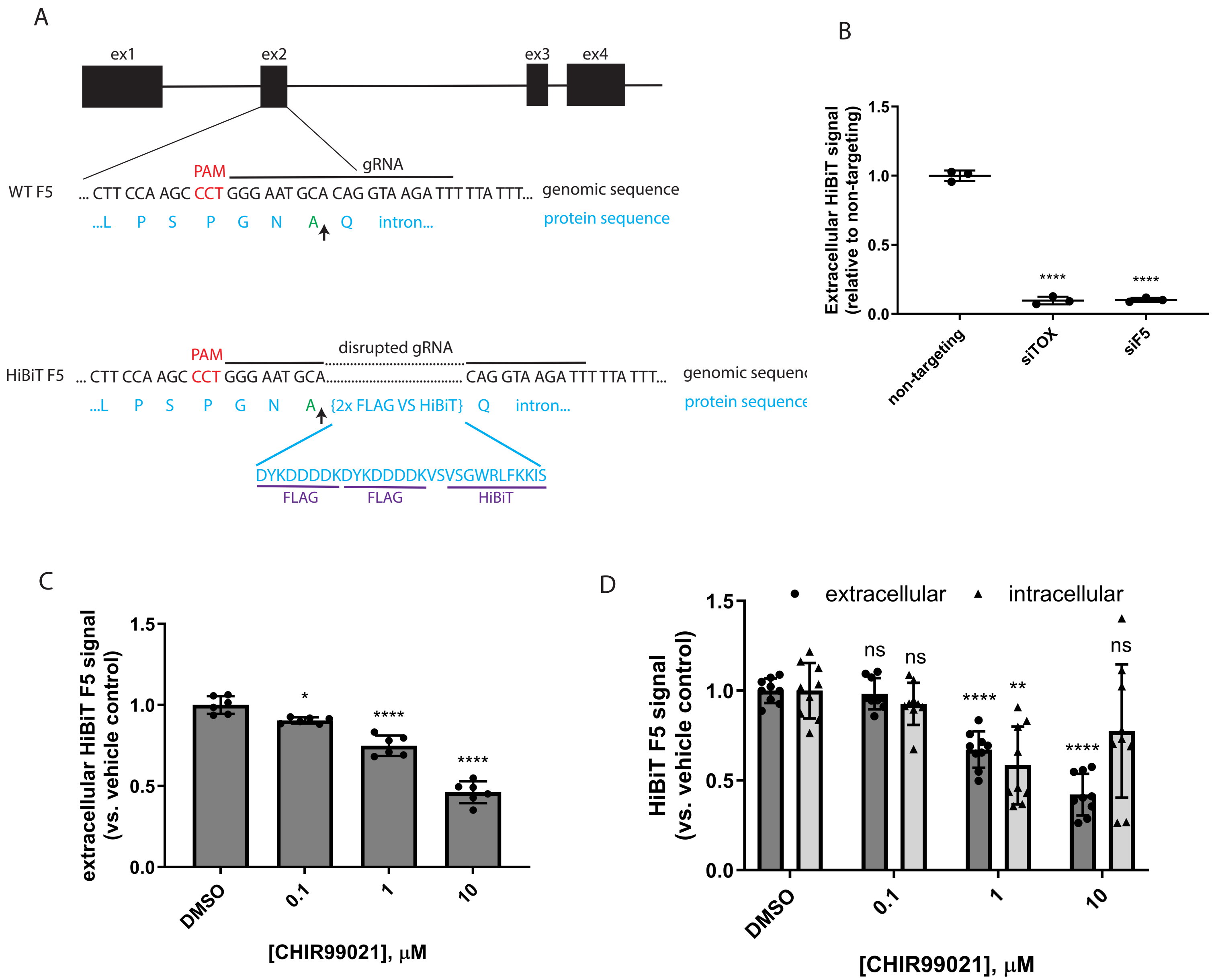

Supplemntal Figure 9.

A

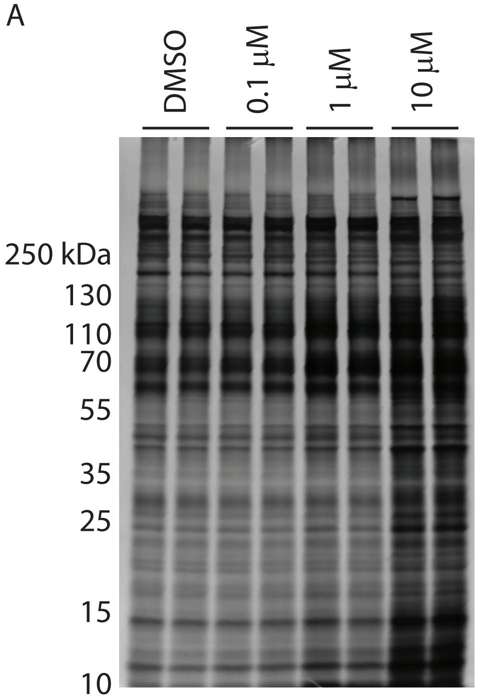

Supplemental Figure 10.

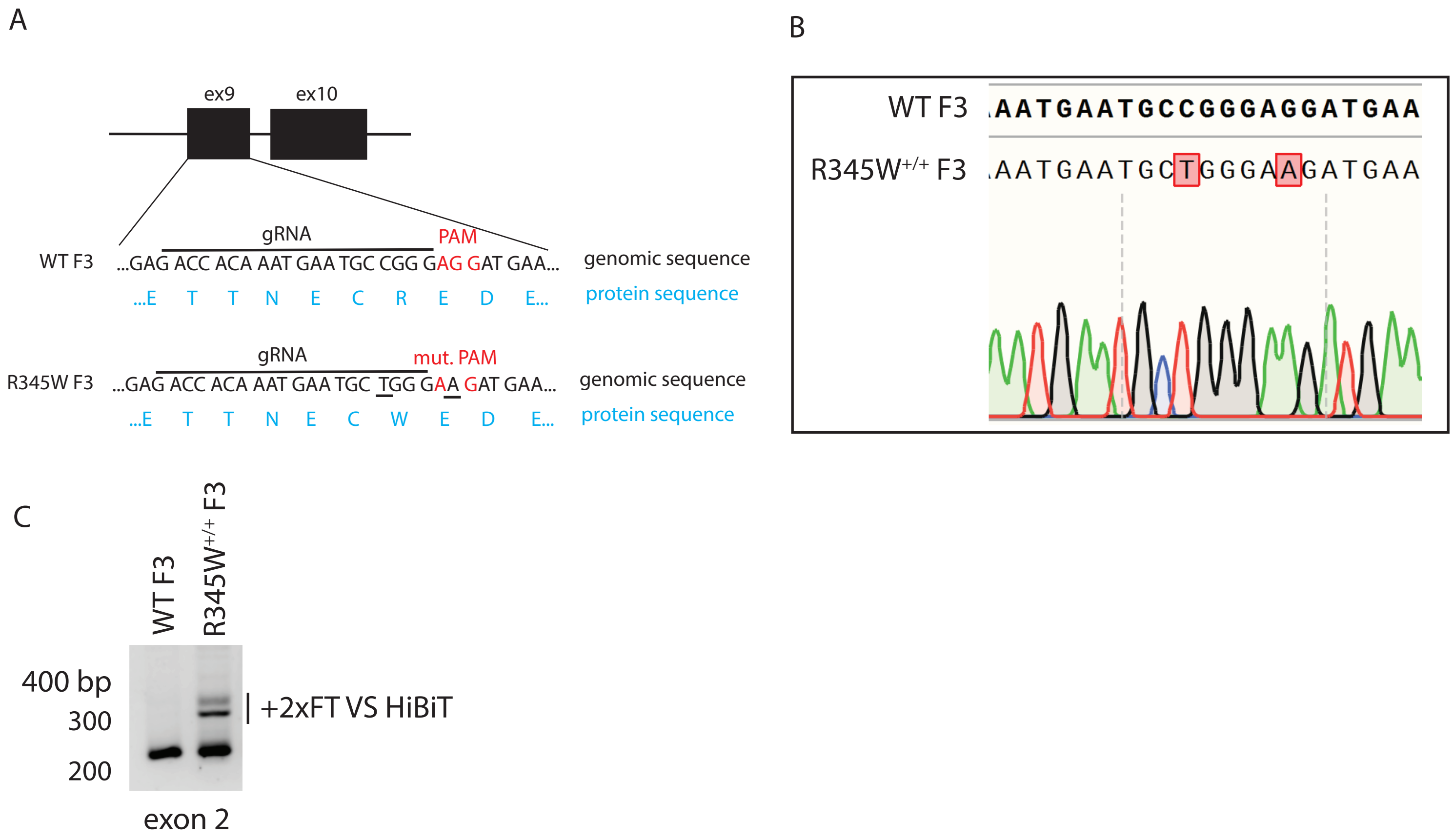

Supplemental Figure 11.

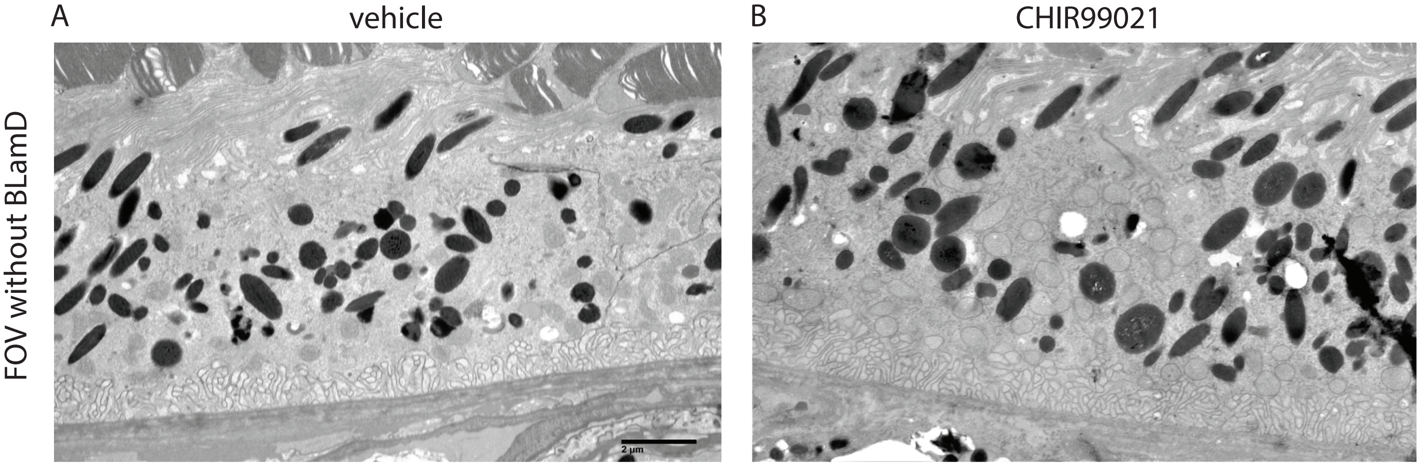

Supplemental Figure 12.

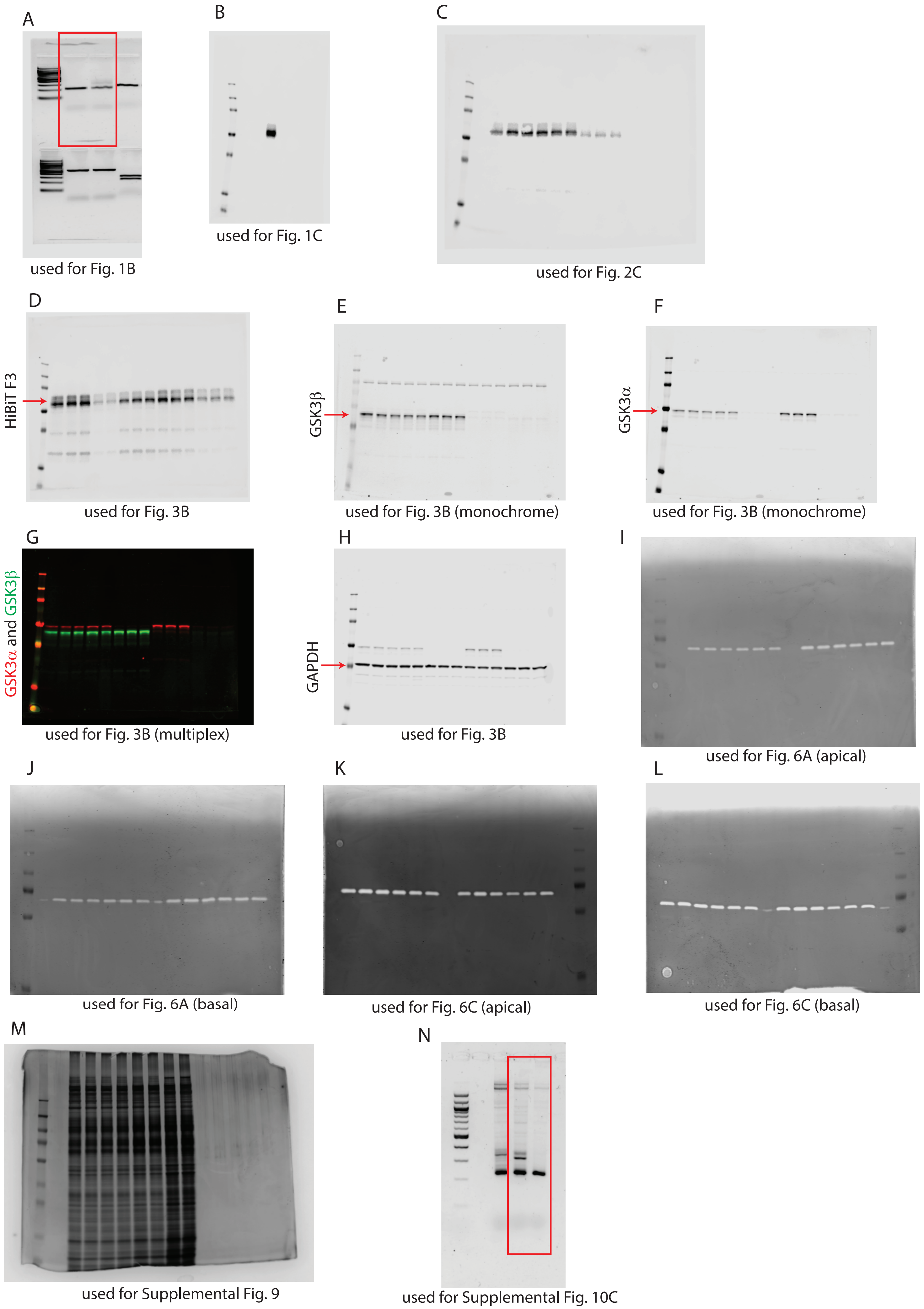

Supplemental Table 1:

**HiBiT edit**	**gRNA**	**PAM**	**ssODN**
2xFLAG VS HiBiT F3 (human)	CGTGTACGTGATGGTTTCTT	CGG	TGTTGAAAGCCCTTTTCCTAACTATGCTGACTCTGGCGCTGGTCAAGTCAG<br>ATTACAAGGATGACGACGATAAGGATTACAAGGATGACGACGATAAGGT<br>GAGCGTGAGCGGCTGGCGGCTGTTCAAGAAGATTAGCCAGGACACtGAA<br>GAAACCATCACGTACACGGTAAGGGGTGATGGAATTTG
2xFLAG VS HiBiT F3 (mouse)	GGTCAAGTCACAGTACACCG	AGG	TGTTGCAAACACTTTTCCTAACTATGCTGACTCTGGCGCTGGTCAAGTCAG<br>ATTACAAGGATGACGACGATAAGGATTACAAGGATGACGACGATAAGGT<br>GAGCGTGAGCGGCTGGCGGCTGTTCAAGAAGATTAGCCAGTACACCGAG<br>GAAACCATCACATACACGGTAAGGCATGATGACATTTG
2xFLAG VS HiBiT F5 (human)	AATCTTACCTGTGCATTCCC	AGG	ACAGGATACTCACTGTTACCATTCTGGCTCTCTGTCTTCCAAGCCCTGGGA<br>ATGCAGATTACAAGGATGACGACGATAAGGATTACAAGGATGACGACGA<br>TAAGGTGAGCGTGAGCGGCTGGCGGCTGTTCAAGAAGATTAGCCAGGTA<br>AGATTTTTATTTTTAAAAAATATCTACAAGCGTATTAAAATCCGGGGTGA

Supplemental Table 2:

**HiBiT edit**	**exon**	**forward primer**	**reverse primer**
2xFLAG VS HiBiT F3 (human)	2	TAAAGTTTTGTTACTTTCCCCGCAGCAGA	GTGTGTAAAGTCTTTTTTTGTCACAGAATCCCG

Supplemental Table 3:

Target	sense sequence
siTOX transfection control	proprietary
non-targeting siRNA control	proprietary
siF3#1 (human)	GCAAUGCACUGACGGAUAUtt
siF3#2 (human)	CAUCCGAUCUGAUAGGUCUtt
siF3 (mouse)	CAUUAAUACUUUUCGGAUUtt
siF5 (human)	GCAGCUCUGUGCGAAUGUUtt
siGSK3α (human)	GAAAGACGAGCUUUACCUAtt
siGSK3β (human)	CUCAAGAACUGUCAAGUAAtt

Supplemental Table 4 - Information on selected hit compounds based on primary, confirmatory, and counterscreening

Structure, SMILES	common name	Target	Pathway	whole well HiBiT F3 (1.66 µM)	whole well HiBiT F3 (5 µM)	media only HiBiT F3 (1.66 µM, counterscreen)	media only HiBiT F3 (5 µM, counterscreen)	CTG (1.66 µM, counterscreen, 48 h Rx)	CTG (5 counte[illegible] , 48 h
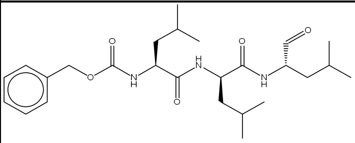 CC(C)C[C@H](NC(=O)[C@@H](CC(C)C)NC(=O)[C@H](CC(C)C)NC(=O)OCc1ccccc1)C=O	MG132	Proteasome, Cysteine Protease	Proteases/Proteasome; Ubiquitination	-55.52	-69.49	-1.61	-1.32	-2.25	-26
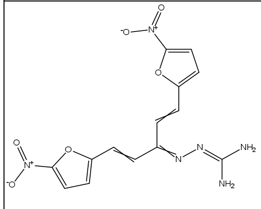 NC(N)=NN=C(C=CC1=CC=C(O1)[N+]([O-])=O)C=CC1=CC=C(O1)[N+]([O-])=O		unknown	unknown	-47.01	-69.62	-5.07	-7.72	7.42	-40
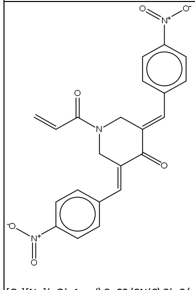 [O-][N+](=O)c1ccc(\C=C2/CN(C\C(=C/c3ccc(cc3)[N+]([O-])=O)C2=O)C(=O)C=C)cc1	B-AP15	DUB inhibitor	Ubiquitination	-38.36	-63.01	-5.24	-5.82	-5.54	-7.
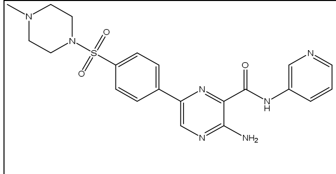 CN1CCN(CC1)S(=O)(=O)C1=CC=C(C=C1)C1=CN=C(N)C(=N1)C(=O)NC1=CN=CC=C1	AZD2858	GSK-3 inhibitor	PI3K/Akt/mTOR signaling; Stem Cells	-37.85	-46.41	-3.60	-6.55	9.55	16.
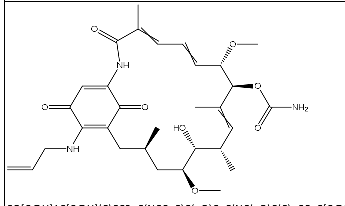 CO[C@H]1C[C@H](C)CC2=C(NCC=C)C(=O)C=C(NC(=O)C(C)=CC=C[C@H](OC)[C@@H](OC(N)=O)C(C)=C[C@H](C)[C@H]1O)C2=	Tanespimycin (17-AAG)	HSP (e.g. HSP90)	Cytoskeletal Signaling	-36.00	-34.14	-0.30	-5.02	-9.89	-12
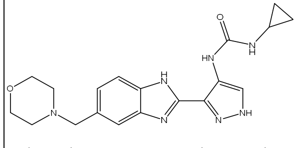 O=C(NC1CC1)NC1=CNN=C1C1=NC2=CC(CN3CCOCC3)=CC=C2N1	AT9283	Aurora Kinase inhibitor; Bcr-Abl inhibitor; JAK inhibitor	Angiogenesis; Cell Cycle/Checkpoint; Chromatin/Epigenetic; Cytoskeletal Signaling; JAK/STAT signaling; Stem Cells	-29.84	-37.86	-0.68	-4.14	10.02	7.4
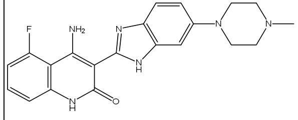 CN1CCN(CC1)C1=CC2=C(C=C1)N=C(N2)C1=C(N)C2=C(NC1=O)C=CC=C2F	Dovitinib (TKI-258, CHIR-258)	c-Kit,FGFR,FLT3,PDGFR,VEGFR	Angiogenesis	-26.64	-44.03	1.94	2.43	-5.14	-21
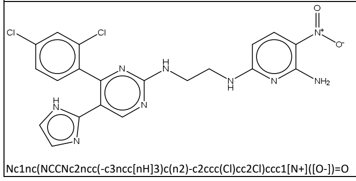 Nc1nc(NCCNc2ncc(-c3ncc[nH]3)c(n2)-c2ccc(Cl)cc2Cl)ccc1[N+]([O-])=O	CHIR98014	GSK-3 inhibitor; S6 Kinase inhibitor	PI3K/Akt/mTOR signaling; Stem Cells	-26.41	-37.93	-4.02	-3.26	32.09	37.

Supplemental Table 5.

accession #	gene name	description	fold change vs. DMSO
Q99470	SDF2	Stromal cell-derived factor 2	13.910
O43660	PLRG1	Pleiotropic regulator 1	9.797
Q9NRV9	HEBP1	Heme-binding protein 1	9.605
P02452	COL1A1	Collagen alpha-1(I)	8.871
P48307	TFPI2	Tissue factor pathway inhibitor 2	4.974
P36955	SERPINF1	Pigment epithelium-derived factor	4.923
P52803	EFNA5	Ephrin-A5	4.225
Q14376	GALE	UDP-glucose 4-epimerase	3.823
P81605	DCD	Dermcidin	3.809
Q5D862	FLG2	Filaggrin-2	3.777
P07311	ACYP1	Acylphosphatase-1	3.681
P61081	UBE2M	NEDD8-conjugating enzyme Ubc12	3.608
O60245	PCDH7	Protocadherin-7	3.484
P39748	FEN1	Flap endonuclease 1	3.471
P49770	EIF2B2	Translation initiation factor eIF-2B subunit beta	3.456
P62266	RPS23	40S ribosomal protein S23	3.443
P20930	FLG	Filaggrin	3.417
Q14112	NID2	Nidogen-2	3.395
O14817	TSPAN4	Tetraspanin-4	3.372
Q92520	FAM3C	Protein FAM3C	3.330
P83916	CBX1	Chromobox protein homolog 1	3.301
Q9UHJ6	SHPK	Sedoheptulokinase	3.228
P61769	B2M	Beta-2-microglobulin	3.169
Q6FI81	CIAPIN1	Anamorsin	3.092
O43488	AKR7A2	Aflatoxin B1 aldehyde reductase member 2	3.060
Q9BXJ0	C1QTNF5	Complement C1q tumor necrosis factor-related protein 5	3.031
Q9NRX4	PHPT1	14 kDa phosphohistidine phosphatase	3.014
P15104	GLUL	Glutamine synthetase	2.966
P21926	CD9	CD9 antigen	2.966
Q9UJU6	DBNL	Drebrin-like protein	2.962
O75663	TIPRL	TIP41-like protein	2.873
P10909	CLU	Clusterin	2.861
P53611	RABGGTB	Geranylgeranyl transferase type-2 subunit beta	2.844
P61204	ARF3	ADP-ribosylation factor 3	2.834
P53990	IST1	IST1 homolog	2.804
P01009	SERPINA1	Alpha-1-antitrypsin	2.801
P27986	PIK3R1	Phosphatidylinositol 3-kinase regulatory subunit alpha	2.793
P52758	RIDA	2-iminobutanoate/2-iminopropanoate deaminase	2.774
P45877	PPIC	Peptidyl-prolyl cis-trans isomerase C	2.748
P33908	MAN1A1	Mannosyl-oligosaccharide 1,2-alpha-mannosidase IA	2.728
O75144	ICOSLG	ICOS ligand	2.727
P17900	GM2A	Ganglioside GM2 activator	2.710
Q13045	FLII	Protein flightless-1 homolog	2.706
Q14956	GPNMB	Transmembrane glycoprotein NMB	2.684
O15400	STX7	Syntaxin-7	2.684
Q8NBJ4	GOLM1	Golgi membrane protein 1	2.665
Q969H8	MYDGF	Myeloid-derived growth factor	2.653
Q9UII2	ATP5IF1	ATPase inhibitor, mitochondrial	2.645
P55196	AFDN	Afadin	2.638
Q92824	PCSK5	Proprotein convertase subtilisin/kexin type 5	2.622

Supplemental Table 6.

GO cellular component complete	# of genes in H. sapiens reference list	# of genes in dataset	expected frequency	fold enrichment	raw p value	FDR
keratohyalin granule (GO:0036457)	4	2	0.03	66.85	8.06E-04	2.94E-02
retromer complex (GO:0030904)	12	3	0.09	33.42	1.71E-04	9.71E-03
platelet alpha granule membrane (GO:0031092)	17	3	0.13	23.59	4.17E-04	1.74E-02
cytosolic small ribosomal subunit (GO:0022627)	45	5	0.34	14.85	3.42E-05	2.59E-03
chaperone complex (GO:0101031)	42	4	0.31	12.73	3.74E-04	1.70E-02
tertiary granule lumen (GO:1904724)	55	5	0.41	12.15	8.31E-05	5.47E-03
lysosomal lumen (GO:0043202)	98	7	0.73	9.55	1.34E-05	1.10E-03
specific granule lumen (GO:0035580)	61	4	0.46	8.77	1.39E-03	4.59E-02
small ribosomal subunit (GO:0015935)	78	5	0.58	8.57	3.85E-04	1.71E-02
platelet alpha granule (GO:0031091)	91	5	0.68	7.35	7.50E-04	2.79E-02

Supplemental Table 7 - List of down regulated genes by RNAseq

gene_id	CH_0323	CH_0407	CH_0603	DMSO_0323	DMSO_0407	DMSO_0603	CHIR	DMSO	log2FoldChange	pvalue	padj
ENSG00000109193	0	0	0	35.89291575	49.51437596	10.73089607	0	32.04606259	-7.527989742	2.41E-07	3.71E-05
ENSG00000101203	0	0	0	27.68882072	18.18895443	24.68106096	0	23.51961204	-7.080422905	1.80E-06	0.000204984
ENSG00000198959	40.78680295	66.11933289	103.386971	681.9653992	685.1172837	321.9268821	70.09770229	563.0031883	-2.996849739	2.01E-18	2.11E-15
ENSG00000188869	121.2870719	55.42355845	54.92432837	431.7405008	645.7078824	692.1427966	77.21165292	589.8637266	-2.942125725	4.17E-21	5.97E-18
ENSG00000159251	55.81351983	79.73213672	116.3103424	380.4649069	790.2090204	600.93018	83.95199966	590.5347024	-2.807025293	2.40E-19	3.15E-16
ENSG00000111186	4.293347679	5.834058784	8.884817824	48.19905829	37.38840634	40.77740507	6.337408096	42.12162323	-2.71346216	5.71E-05	0.003320259
ENSG00000130720	10.7333692	19.44686261	8.884817824	69.73480773	122.2701937	45.0697635	13.02168321	79.02492164	-2.607611205	3.65E-06	0.000378607
ENSG00000156466	13.95337996	25.2809214	6.46168569	75.88787901	184.9210367	13.95016489	15.23199568	91.58636021	-2.596109033	0.000259883	0.010823946
ENSG00000179772	31.12677067	56.39590158	23.42361063	210.2299351	272.8343165	121.2591256	36.98209429	201.4411257	-2.450598997	2.19E-09	5.30E-07
ENSG00000172575	157.7805272	140.0174108	196.2737028	1041.920069	775.0515584	876.714209	164.690547	897.8952786	-2.443875171	3.30E-29	1.73E-25
ENSG00000259207	69.76689978	36.94903897	128.4260031	425.5874296	349.6321241	439.9667389	78.38064728	405.0620975	-2.361751506	1.36E-11	5.22E-09
ENSG00000186994	10.7333692	6.806401915	7.269396402	36.91842762	54.5668633	32.19268821	8.269722505	41.22599305	-2.326471352	0.000386502	0.01439377
ENSG00000173597	16.1000538	35.00435271	10.50023925	136.3930798	97.00775698	61.16610761	20.53488192	98.18898147	-2.264741002	1.45E-05	0.001156829
ENSG00000171385	89.08696434	47.64481341	110.6563674	396.873097	403.1884899	346.6079431	82.46271506	382.2231767	-2.208572081	2.06E-13	9.55E-11
ENSG00000238271	10.7333692	18.47451948	8.077107113	48.19905829	72.75581773	46.14285311	12.42833193	55.69924304	-2.171203285	0.000184593	0.008284583
ENSG00000081052	2553.468532	1604.366166	1811.695125	9845.939545	9411.773422	7119.949543	1989.843274	8792.55417	-2.14401394	3.24E-27	1.28E-23
ENSG00000168398	16.1000538	18.47451948	7.269396402	73.83685525	39.40940127	69.75082446	13.94798989	60.999027	-2.141118718	0.000185631	0.008307528
ENSG00000166448	12.88004304	9.723431307	25.84674276	25.63779696	93.97626457	94.43188543	16.15007237	71.34864899	-2.130761546	0.000475409	0.016849972
ENSG00000113578	51.52017215	20.41920575	15.34650351	113.8318185	145.5116355	116.9667672	29.0952938	125.4367404	-2.124047646	3.97E-06	0.000406559
ENSG00000165879	26.83342299	4.861715654	16.96192494	60.50520083	83.87128989	66.53155564	16.21902119	70.30268212	-2.122518655	0.000216576	0.009450763
ENSG00000146674	10175.234	11419.19773	7134.508713	42943.30991	52576.18329	28831.77156	9576.31348	41450.42159	-2.113920173	8.20E-22	1.29E-18
ENSG00000169891	47.22682447	33.05966644	58.96288192	237.9187558	200.0784988	163.1096203	46.41645761	200.3689583	-2.10525471	6.53E-10	1.77E-07
ENSG00000120937	1270.830913	2004.971536	931.2904501	3894.894114	7738.389614	6373.079177	1402.3643	6002.120968	-2.097734356	7.13E-07	9.94E-05
ENSG00000236333	9.660032278	3.889372523	29.07758561	49.22457017	55.57736077	78.33554132	14.2089968	61.04582409	-2.08164626	0.001331583	0.036369598
ENSG00000160963	16.1000538	10.69577444	9.692528536	54.35212956	62.65084305	34.33886743	12.16278559	50.44728001	-2.062556551	0.000459204	0.016403251
ENSG00000139946	69.76689978	56.39590158	33.11613916	336.3678961	206.1414836	104.0896919	53.09298018	215.5330239	-2.028186736	1.34E-06	0.000162561
ENSG00000164849	13.95337996	13.61280383	17.76963565	55.37764144	90.94477217	38.63122586	15.11193981	61.65121315	-2.024561623	0.000250924	0.010625811
ENSG00000072657	68.69356286	53.47887219	128.4260031	244.0718271	259.6978494	515.0830114	83.53281272	339.6175626	-2.017013697	4.04E-08	7.00E-06
ENSG00000101605	8.586695358	13.61280383	12.11566067	34.86740387	52.54586836	51.50830114	11.43838662	46.30719112	-2.012208742	0.000738219	0.023493267
ENSG00000170379	47.22682447	25.2809214	29.07758561	202.0258401	133.3856658	60.093018	33.86177716	131.8348413	-1.966933613	2.78E-05	0.001960099
ENSG00000141052	45.08015063	14.58514696	59.77059264	83.06646215	189.9735241	193.1561293	39.81196341	155.3987052	-1.959580871	3.86E-05	0.002503575
ENSG00000138623	252.2341761	240.1687533	206.7739421	854.2513948	964.014585	874.5680298	233.0589572	897.6113365	-1.947439929	3.61E-24	8.12E-21
ENSG00000010310	12.88004304	4.861715654	26.65445347	38.96945138	70.7348228	63.31228682	14.79873739	57.672187	-1.946877622	0.001890538	0.046900229
ENSG00000233818	16.1000538	32.08732331	12.92337138	109.729771	75.78731014	48.28903232	20.3702495	77.93537115	-1.940866785	0.000252985	0.01068437
ENSG00000176678	15.02671688	12.6404607	37.15469272	72.81134337	86.90278229	89.06643739	21.6072901	82.92685435	-1.92369429	0.000146571	0.006975633
ENSG00000186479	1028.256769	425.8862913	1159.872581	3808.751117	2687.923266	3225.707359	871.3385473	3240.793914	-1.894898438	1.88E-05	0.001434154
ENSG00000149591	971.3699124	1516.855284	858.5964861	3300.097225	5703.247712	3397.401696	1115.607227	4133.582211	-1.8898532	8.24E-15	4.06E-12
ENSG00000172061	7.513358438	14.58514696	31.50071774	42.04598702	86.90278229	63.31228682	17.86640771	64.08701871	-1.82476002	0.001984651	0.048850328
ENSG00000235750	9.660032278	14.58514696	19.38505707	45.12252265	64.67183799	45.0697635	14.5434121	51.62137471	-1.816521157	0.001321055	0.036192298
ENSG00000198542	51.52017215	56.39590158	64.6168569	227.663637	272.8343165	107.3089607	57.51097688	202.6023047	-1.814981423	1.06E-06	0.000133348
ENSG00000169031	1150.617178	1079.300875	641.3223048	3563.653778	3927.80366	2572.195788	957.0801193	3354.551075	-1.810464455	1.59E-15	9.25E-13
ENSG00000100027	147.047158	47.64481341	84.00191397	406.1027039	303.1492406	248.9567889	92.89796179	319.4029111	-1.78584993	5.71E-07	8.03E-05
ENSG00000152822	27.90675991	37.9213821	29.07758561	114.8573304	147.5326304	63.31228682	31.63524254	108.5674159	-1.78007471	4.81E-05	0.002904996
ENSG00000106366	5799.239377	6297.866458	7290.39688	18092.08056	26781.21441	21594.85525	6462.500905	22156.05007	-1.777435712	4.90E-24	9.65E-21
ENSG00000213694	22.54007531	11.66811757	17.76963565	49.22457017	69.72432533	57.94683879	17.32594284	58.96524476	-1.770262374	0.000775055	0.024370148
ENSG00000135318	716.9890624	535.761065	921.5979126	2801.698452	2140.233638	2449.863573	724.782683	2463.931888	-1.764367706	2.29E-17	1.90E-14
ENSG00000165072	847.9361666	1108.471169	828.7111898	2834.514832	4182.449022	2331.823716	928.3728418	3116.262524	-1.747253279	1.19E-15	7.23E-13
ENSG00000126561	45.08015063	7.778745046	33.11613916	73.83685525	73.7663152	140.5747385	28.65834495	96.059303	-1.746612643	0.001374506	0.037012981
ENSG00000113083	9751.265916	8842.488431	6430.992683	32886.11492	30966.69492	18184.57648	8341.582343	27345.79544	-1.713026163	6.12E-15	3.21E-12
ENSG00000005187	34.34678143	35.97669584	29.07758561	148.6992224	139.4486507	35.41195704	33.13368762	107.8532767	-1.705443103	0.000734519	0.023422817
ENSG00000118523	11214.22414	12467.38362	10999.40447	33537.31496	45229.86669	33349.47881	11560.33741	37372.22015	-1.69272902	6.41E-25	1.68E-21
ENSG00000111859	668.688901	588.2675941	772.9791507	2302.274167	2068.488318	2160.129379	676.6452153	2176.963955	-1.685014264	3.65E-23	6.39E-20
ENSG00000249456	31.12677067	13.61280383	42.8086677	56.40315331	115.1967114	109.4551999	29.1827474	93.68500155	-1.676335011	0.000758411	0.023990451
ENSG00000131738	1355.62453	1385.588961	2075.008817	3976.935065	6000.333968	5338.620796	1605.407436	5105.296609	-1.66844345	8.76E-16	5.52E-13
ENSG00000165959	68.69356286	7.778745046	55.73203908	137.4185917	147.5326304	129.8438425	44.06811566	138.2650215	-1.650321147	0.001429585	0.038234727
ENSG00000187689	595.7019904	1207.650168	656.6688083	2079.73809	3493.289749	2062.478225	820.066989	2545.168688	-1.634169926	7.06E-10	1.86E-07
ENSG00000169991	642.9288149	445.3331539	530.6659373	1388.543083	1914.892703	1716.943371	539.6426354	1673.459719	-1.633440365	2.04E-16	1.53E-13
ENSG00000140015	33.27344451	47.64481341	76.73251757	175.3625312	148.5431279	164.1827099	52.5502585	162.696123	-1.620348178	9.98E-06	0.000850083
ENSG00000260604	500.1750046	397.6883405	518.5502767	1627.487351	1385.392029	1279.122812	472.1378739	1430.667397	-1.599063337	1.69E-17	1.48E-14
ENSG00000079841	129.8737673	213.9154888	334.3922345	502.5008204	776.0620558	772.6245171	226.0604968	683.7291311	-1.592726917	9.63E-08	1.60E-05
ENSG00000261051	31.12677067	27.22560766	19.38505707	102.5511878	68.71382786	57.94683879	25.91247847	76.4039515	-1.56720895	0.000876285	0.026641313
ENSG00000106688	170.6605702	146.8238127	124.3874495	467.6334166	458.7658507	374.5082729	147.2906108	433.6358467	-1.561210427	5.75E-11	1.93E-08
ENSG00000164694	98.74699661	97.23431307	43.61637841	243.0463152	350.6426216	106.2358711	79.86589603	233.3082693	-1.55181048	0.000195497	0.008675108
ENSG00000139211	157.7805272	144.8791265	270.5830883	543.5212956	682.0857913	446.4052766	191.080914	557.3374545	-1.540690469	2.00E-08	3.80E-06
ENSG00000146005	24.68674915	22.36389201	30.69300703	92.29606906	58.60885318	75.1162725	25.9145494	75.34039825	-1.535424291	0.00078188	0.024414154
ENSG00000206538	6245.747536	4304.56304	5523.933555	17357.81405	14030.75735	15154.17143	5358.081377	15514.24761	-1.53385329	6.89E-19	8.35E-16
ENSG00000166106	718.0623993	483.254536	555.7049694	1559.803567	1844.15788	1631.096203	585.6739682	1678.35255	-1.519859595	1.89E-15	1.06E-12
ENSG00000171401	85.86695358	57.36824471	80.7710717	172.2859956	285.9707836	178.1328748	74.66875647	212.1298847	-1.506851334	4.33E-06	0.000431712
ENSG00000186352	68.69356286	135.1556952	105.0023925	352.7760862	275.8658089	245.73752	102.9505502	291.459805	-1.499065883	7.35E-07	0.000101338
ENSG00000011028	287.6542945	140.0174108	270.5830883	614.2816152	658.8443495	699.6544239	232.7515979	657.5934628	-1.49821201	7.36E-09	1.61E-06
ENSG00000082497	310.1943698	268.3667041	303.6992274	837.8432047	825.5764318	819.8404599	294.0867671	827.7533654	-1.492966695	6.46E-16	4.24E-13
ENSG00000118515	232.9141116	358.7946152	316.6225988	692.220518	835.6814065	963.6344672	302.7771085	830.5121306	-1.454300028	6.93E-11	2.28E-08
ENSG00000108821	1792.472656	2918.001735	1432.878802	4397.394935	8115.30517	4202.218902	2047.784398	5571.639669	-1.44410746	0.001532744	0.040175245
ENSG00000049192	27.90675991	24.30857827	31.50071774	64.60724834	78.81880255	82.62789975	27.90535197	75.35131688	-1.43039434	0.001154711	0.032598867
ENSG00000171219	37.56679219	42.78309775	81.57878184	115.8828423	134.3961633	188.8637709	53.97622393	146.3809255	-1.430104757	0.00027673	0.011411844
ENSG00000103647	64.40021518	53.47887219	37.15469272	179.4645787	148.5431279	85.84716857	51.6779267	137.9516251	-1.422864007	0.000306309	0.012154356
ENSG00000166482	90.16030126	78.75979359	49.27035339	149.7247343	262.7293418	163.1096203	72.73014941	191.8545655	-1.405302866	6.61E-05	0.003734219
ENSG00000123843	31.12677067	90.42791116	46.84722126	143.571663	178.8580519	123.4053048	56.13396769	148.6116732	-1.403785946	0.000536644	0.018407569
ENSG00000138356	297.3143268	244.0581258	441.8177591	950.6495113	847.8073761	804.8172054	327.7300706	867.7580309	-1.402359644	1.59E-09	3.97E-07
ENSG00000159263	79.42693206	48.61715654	72.69396402	232.7911964	191.994519	99.79733346	66.9126842	174.8610163	-1.386744014	0.000229401	0.009846741
ENSG00000166145	48.30016139	60.2852741	55.73203908	103.5766997	122.2701937	198.5215773	54.77249152	141.4561569	-1.366827787	0.000219911	0.009569777
ENSG00000113389	1628.252107	1080.273218	1870.658007	4008.725933	3347.778143	4363.182343	1526.394444	3906.56213	-1.35542502	3.81E-11	1.30E-08
ENSG00000115380	11189.53739	10246.55191	7366.321687	30725.36139	27095.47912	15691.78933	9600.803662	24504.20995	-1.35188662	2.75E-09	6.57E-07
ENSG00000101333	5509.438409	3896.178925	5698.399068	15208.34116	11337.7816	11901.63683	5034.672134	12815.91986	-1.347923124	3.86E-13	1.74E-10
ENSG00000079931	97.6736597	98.2066562	134.8876888	328.1638011	300.1177482	210.325563	110.2560016	279.5357041	-1.339133599	3.01E-06	0.000316285
ENSG00000119681	4977.063297	3911.736415	4549.026726	10741.21141	12504.90617	10745.91693	4479.275479	11330.67897	-1.338959653	8.70E-18	8.56E-15
ENSG00000146094	111.6270397	90.42791116	79.96336042	280.9902547	291.0232709	131.9900217	94.00610374	234.6678491	-1.323013241	9.85E-05	0.005135705
ENSG00000243753	63.32687826	72.9257348	35.5392713	152.8012699	199.0680013	74.04318289	57.26396145	141.970818	-1.315299516	0.001966987	0.048491303
ENSG00000027075	75.13358438	67.09167602	75.92480686	186.6431619	203.1099912	152.3787242	72.71668909	180.7106258	-1.31332388	1.52E-05	0.001201646
ENSG00000175274	991.7633138	788.570279	482.2032946	1778.237597	2268.566817	1513.056346	754.1789625	1853.28692	-1.298541401	9.00E-08	1.51E-05
ENSG00000162373	114.8470504	81.67682298	65.42456762	236.8932439	226.351433	177.0597852	87.316147	213.4348207	-1.295314878	3.31E-05	0.002248249
ENSG00000074590	472.2682447	290.7305961	547.6278623	949.6239994	1140.851642	1113.867012	436.8755677	1068.114218	-1.288802278	2.32E-08	4.30E-06
ENSG00000153162	2153.113861	1736.604831	1340.799781	4845.543626	4712.960193	3214.976463	1743.506158	4257.826761	-1.288711946	7.19E-10	1.86E-07
ENSG00000004799	59.03353058	75.8427642	63.80914619	236.8932439	123.2806912	124.4783944	66.22848032	161.5507765	-1.286366043	0.000387476	0.014396019
ENSG00000175161	172.8072441	246.0028121	160.7344315	598.898937	434.5139115	366.9966456	193.1814959	466.8031647	-1.273895983	1.38E-06	0.000164452
ENSG00000141738	201.7873409	151.6855284	114.694921	251.2504102	552.7421153	314.4152549	156.0559301	372.8025935	-1.259635868	7.85E-05	0.004279865
ENSG00000198948	326.2944236	316.0115175	358.6235558	741.4450881	816.4819546	831.6444455	333.6431656	796.5238294	-1.254542314	1.39E-11	5.22E-09
ENSG00000177181	728.7957685	286.8412236	455.5488412	1592.619947	1076.179804	784.4285028	490.3952777	1151.076085	-1.231968955	4.00E-05	0.002552983
ENSG00000157680	38.64012911	47.64481341	84.00191397	102.5511878	162.6900924	136.2823801	56.7622855	133.8412201	-1.229424144	0.001703509	0.043169597
ENSG00000075618	825.3960913	1175.562845	1155.834028	1600.824042	2479.760788	3296.531273	1052.264321	2459.038701	-1.224098213	2.73E-07	4.14E-05
ENSG00000187498	11348.39125	8946.529146	9572.98735	20878.39633	27637.10576	21216.05462	9955.969249	23243.85224	-1.2232707	4.40E-13	1.93E-10
ENSG00000257671	136.3137888	129.3216364	127.6182924	306.6280517	364.7895861	245.73752	131.0845725	305.7183859	-1.222734888	2.25E-06	0.000247918
ENSG00000144583	186.760624	188.6345674	250.3903205	316.8831704	587.0990292	546.20261	208.5951706	483.3949366	-1.210547865	6.67E-06	0.000636951
ENSG00000115414	8505.121752	4717.80887	8098.107591	19262.18961	16107.32965	13969.48051	7107.012738	16446.33326	-1.210450708	1.20E-08	2.42E-06
ENSG00000058335	48.30016139	67.09167602	63.00143548	149.7247343	127.322681	136.2823801	59.4644243	137.7765985	-1.209506455	0.000304785	0.012154356
ENSG00000108846	64.40021518	59.31293097	90.46359967	123.0614254	244.5403874	125.551484	71.39224861	164.3844323	-1.20024909	0.001223801	0.034155058
ENSG00000185100	134.167115	224.6112632	180.1194886	410.2047514	447.6503786	377.7275417	179.6326223	411.8608906	-1.195768963	1.62E-06	0.000187538
ENSG00000079308	774.949256	317.9562037	460.3951054	1237.792837	1215.628455	1099.916847	517.7668551	1184.446046	-1.195149851	4.66E-06	0.000458463
ENSG00000020181	84.79361666	64.17464663	102.5792603	179.4645787	190.9840216	200.6677565	83.84917454	190.3721189	-1.179858231	0.000103935	0.005382374
ENSG00000073464	169.5872333	142.9344402	206.7739421	414.3067989	403.1884899	360.558108	173.0985385	392.6844656	-1.179647212	7.40E-07	0.000101338
ENSG00000136490	49.37349831	81.67682298	51.69348552	95.3726047	181.8895443	135.2092905	60.91460227	137.4904798	-1.175158083	0.001962981	0.048468412
ENSG00000214517	3160.977229	3618.088789	3577.35074	7084.236056	8574.071021	7705.856469	3452.138919	7788.054515	-1.173657304	6.43E-15	3.27E-12
ENSG00000198053	2294.794334	1920.377683	2408.593341	4212.802797	5352.605091	5347.205512	2207.921786	4970.871133	-1.170679729	2.78E-12	1.15E-09
ENSG00000181274	65.4735521	102.0960287	82.38649255	175.3625312	222.3094431	162.0365307	83.31869113	186.5695017	-1.161838892	0.000203564	0.008982479
ENSG00000112139	5478.311638	4825.738958	4252.596895	9041.938232	12606.96642	10903.6635	4852.21583	10850.85605	-1.161253741	1.28E-11	5.02E-09
ENSG00000261068	46.15348755	70.98104854	52.50119623	139.4696155	94.98676204	144.867097	56.54524411	126.4411582	-1.160036435	0.001516947	0.039893922
ENSG00000144642	829.6894389	567.8483883	702.7083188	1865.406107	1446.021877	1364.96998	700.0820487	1558.799322	-1.155313054	8.00E-09	1.70E-06
ENSG00000235609	134.167115	125.4322639	151.041903	355.8526218	240.4983975	318.7076133	136.8804273	305.0195442	-1.154477366	9.68E-06	0.000833403
ENSG00000125730	137.3871257	60.2852741	165.5806958	247.1483627	233.4249152	328.3654198	121.0843652	269.6462326	-1.152602425	0.00076951	0.024244185
ENSG00000105856	968.1499016	519.2312318	877.9815432	2053.074781	1694.604255	1505.544719	788.4542255	1751.074585	-1.151254728	4.22E-07	6.16E-05
ENSG00000133424	431.4814417	288.7859098	493.5112446	808.1033602	1053.94886	833.7906248	404.5928654	898.6142816	-1.150386474	4.38E-07	6.33E-05
ENSG00000164176	1421.098082	1062.771042	1109.794517	3338.041164	2520.180687	2111.840347	1197.88788	2656.687399	-1.149616446	1.02E-08	2.09E-06
ENSG00000054277	238.2807962	174.0494204	189.0043064	452.2507384	501.2067444	378.8006313	200.444841	444.086038	-1.149551869	9.28E-07	0.000120873
ENSG00000185499	368.1545635	246.9751552	233.4243956	733.2409931	747.7681267	398.1162433	282.8527047	626.3751214	-1.149115796	2.58E-05	0.001853962
ENSG00000213713	104.1136812	53.47887219	99.34841749	266.6330884	141.4696456	160.9634411	85.6469903	189.688725	-1.146944454	0.001488031	0.039330447
ENSG00000164197	447.5814955	346.1541545	421.6249913	1078.838496	815.4714571	793.0132197	405.1202138	895.774391	-1.145001197	2.41E-08	4.41E-06
ENSG00000001617	155.6338534	103.0683719	96.11757464	232.7911964	284.9602661	263.9800434	118.2732666	260.5771753	-1.14388702	5.36E-05	0.003160929
ENSG00000114796	1245.070827	1129.862718	1303.645088	3276.510452	2240.272888	2595.80376	1226.192878	2704.1957	-1.140860585	2.36E-10	7.02E-08
ENSG00000133216	792.1226468	543.5398101	657.476519	1396.747178	1836.0739	1148.20588	664.3796586	1460.342319	-1.136817909	1.27E-07	2.03E-05
ENSG00000152409	807.1493636	457.9736146	854.5579326	1755.676336	1342.951136	1535.591228	706.5603036	1544.739566	-1.128101963	8.28E-07	0.000109619
ENSG00000165312	805.0026898	612.5761724	763.2866222	1122.935507	1473.305309	2151.544662	726.9551614	1582.595159	-1.122251601	1.04E-06	0.000133022
ENSG00000108379	64.40021518	68.06401915	134.0799781	170.2349718	174.8160621	236.0797136	88.8480708	193.7102492	-1.117466144	0.001369055	0.036929322
ENSG00000170442	562.4285459	484.2268791	240.697792	820.4095028	1110.536718	841.302252	429.117739	924.0828242	-1.10891946	4.00E-05	0.002552983
ENSG00000168453	370.3012373	260.587959	277.044774	351.7505743	735.6421571	868.1294922	302.6446568	651.8407412	-1.107661965	0.000141977	0.006818793
ENSG00000278948	62.25354134	139.0450677	185.7734636	244.0718271	320.3276975	271.4916706	129.0240242	278.6303984	-1.105433193	0.001254424	0.034801769
ENSG00000133107	485.1482877	315.0391744	479.7801625	1128.063066	748.7786242	852.0331481	426.6558749	909.6249462	-1.092002709	2.12E-06	0.00023634
ENSG00000102452	183.5406133	179.8834792	195.4659921	481.9905829	410.2619722	298.3189108	186.2966949	396.8571553	-1.09081472	1.05E-05	0.000887127
ENSG00000154736	3217.864085	2346.263974	4669.375622	6398.16861	6503.561708	8684.514191	3411.167894	7195.414836	-1.076532279	1.51E-06	0.000177623
ENSG00000173801	2054.366864	1566.444784	1454.686991	3673.383549	3731.767151	3289.019646	1691.83288	3564.723449	-1.075810326	6.16E-10	1.70E-07
ENSG00000151414	2188.533979	1212.511884	2090.355321	4880.41103	3475.100794	3216.049553	1830.467061	3857.187125	-1.075333112	2.37E-06	0.000257643
ENSG00000073910	225.4007531	156.547244	268.1599562	517.8834986	408.2409773	445.332187	216.7026511	457.152221	-1.07522956	1.61E-05	0.001247406
ENSG00000011201	1721.632419	1203.760796	1459.533255	3670.307013	3082.017279	2472.398455	1461.642157	3074.907582	-1.073224863	3.40E-08	6.02E-06
ENSG00000091592	4218.214095	4394.018608	3739.700593	5959.249526	9073.25677	10909.02895	4117.311099	8647.178414	-1.070574562	1.23E-07	1.99E-05
ENSG00000127329	630.0487719	427.8309775	932.0981608	1600.824042	1277.2688	1293.072977	663.3259701	1390.388606	-1.066519387	1.34E-05	0.001090702
ENSG00000122707	934.8764571	640.7741231	830.3266112	1790.54374	1628.921919	1612.85368	801.9923971	1677.43978	-1.064878553	7.21E-09	1.60E-06
ENSG00000100302	75.13358438	116.6811757	139.7339531	223.5615895	298.0967532	172.7674268	110.5162377	231.4752565	-1.062786445	0.000970127	0.028889233
ENSG00000112414	296.2409898	205.1644006	545.2047301	830.6646215	557.7946026	798.3786677	348.8700402	728.945964	-1.060747876	0.000385316	0.014383616
ENSG00000169432	592.4819797	286.8412236	406.2784878	1080.88952	882.16429	695.3620654	428.533897	886.1386251	-1.049318107	5.11E-05	0.003036277
ENSG00000166046	317.7077282	281.0071648	357.0081344	832.7156453	514.3432115	627.7574202	318.5743425	658.2720923	-1.046234932	4.82E-06	0.000471893
ENSG00000173894	105.1870181	119.5982051	97.73299607	230.7401727	243.5298899	189.9368605	107.5060731	221.4023077	-1.043631547	0.000149536	0.007073973
ENSG00000132434	833.9827866	799.2660535	703.5160295	1528.012699	1887.609271	1391.79722	778.9216232	1602.473064	-1.041462531	8.78E-09	1.82E-06
ENSG00000092295	954.1965216	1031.656062	838.4037183	1764.905943	2130.128664	1900.441694	941.4187672	1931.825434	-1.037566978	7.12E-10	1.86E-07
ENSG00000211455	1326.644433	1239.737492	1401.378084	3057.05091	2691.965256	2396.209093	1322.58667	2715.075086	-1.03750029	1.68E-10	5.20E-08
ENSG00000095951	909.116371	721.478603	1267.298106	2189.46786	1753.213108	2002.385207	965.96436	1981.688725	-1.035781778	8.50E-07	0.000111639
ENSG00000120162	68.69356286	124.4599207	118.7334746	255.3524577	191.994519	192.0830397	103.9623194	213.1433388	-1.032240917	0.00098687	0.029167296
ENSG00000100311	191.0539717	197.3856555	181.73491	382.5159307	452.7028659	325.146151	190.0581791	386.7883158	-1.025928969	1.05E-05	0.000887127
ENSG00000135245	306.974359	265.4496747	186.5811743	563.0060213	621.4559432	354.1195704	253.001736	512.8605116	-1.022102001	0.000135882	0.006579728
ENSG00000152779	374.594585	290.7305961	570.2437622	727.0879218	761.9150913	1016.215858	411.8563144	835.072957	-1.017772277	5.17E-05	0.003061266
ENSG00000091986	28882.42317	13070.23636	17390.81932	47428.89887	41577.92884	30748.3096	19781.15962	39918.3791	-1.012967809	2.97E-05	0.002071007
ENSG00000122786	14457.84831	10156.124	11257.87189	26035.69557	25997.06837	20338.26732	11957.2814	24123.67709	-1.012623704	8.60E-09	1.81E-06
ENSG00000187398	236.1341223	238.224067	369.1237951	543.5212956	647.7288773	511.8637426	281.1606615	567.7046385	-1.011092187	2.09E-05	0.001550309
ENSG00000004399	2775.649274	2085.676015	1749.501401	4156.399643	5795.202982	3354.478112	2203.608897	4435.360246	-1.009610767	4.27E-06	0.000428885
ENSG00000138641	249.0141654	217.8048613	218.8896628	626.5877577	399.1465081	353.0464808	228.5695414	459.5935795	-1.008626007	8.27E-05	0.004461722
ENSG00000130518	130.9471042	121.5428913	140.5416638	158.9543412	285.9707836	344.4617639	131.0105531	263.1289629	-1.005091903	0.001163263	0.032722998
ENSG00000163235	341.3211405	268.3667041	273.0062204	653.2510666	708.3587254	407.7740507	294.231355	589.7946162	-1.004642258	4.09E-05	0.002591123

gene_id	gene_name	gene_chr	gene_start	gene_end	gene_strand	gene_length	gene_biotype	gene_description	tf_family
ENSG00000109193	SULT1E1	4	69841212	69860152	-	2091	protein_coding	sulfotransferase family 1E member 1 [Source:HGNC Symbol;Acc:HGNC:11377]	-
ENSG00000101203	COL20A1	20	63293186	63334851	+	10974	protein_coding	collagen type XX alpha 1 chain [Source:HGNC Symbol;Acc:HGNC:14670]	-
ENSG00000198959	TGM2	20	38127387	38166578	-	8036	protein_coding	transglutaminase 2 [Source:HGNC Symbol;Acc:HGNC:11778]	-
ENSG00000188869	TMC3	15	81331217	81374213	-	4848	protein_coding	transmembrane channel like 3 [Source:HGNC Symbol;Acc:HGNC:22995]	-
ENSG00000159251	ACTC1	15	34788095	34795726	-	4602	protein_coding	actin, alpha, cardiac muscle 1 [Source:HGNC Symbol;Acc:HGNC:143]	-
ENSG00000111186	WNT5B	12	1529891	1647243	+	4305	protein_coding	Wnt family member 5B [Source:HGNC Symbol;Acc:HGNC:16265]	-
ENSG00000130720	FIBCD1	9	130902438	130939286	-	3831	protein_coding	fibrinogen C domain containing 1 [Source:HGNC Symbol;Acc:HGNC:25922]	-
ENSG00000156466	GDF6	8	96142330	96160792	-	3701	protein_coding	growth differentiation factor 6 [Source:HGNC Symbol;Acc:HGNC:4221]	-
ENSG00000179772	FOXS1	20	31844301	31845619	-	1319	protein_coding	forkhead box S1 [Source:HGNC Symbol;Acc:HGNC:3735]	Fork
ENSG00000172575	RASGRP1	15	38488103	38565575	-	6097	protein_coding	RAS guanyl releasing protein 1 [Source:HGNC Symbol;Acc:HGNC:9878]	-
ENSG00000259207	ITGB3	17	47253846	47311816	+	4394	protein_coding	integrin subunit beta 3 [Source:HGNC Symbol;Acc:HGNC:6156]	-
ENSG00000186994	KANK3	19	8322584	8343262	-	3594	protein_coding	KN motif and ankyrin repeat domains 3 [Source:HGNC Symbol;Acc:HGNC:24796]	-
ENSG00000173597	SULT1B1	4	69721162	69787961	-	7356	protein_coding	sulfotransferase family 1B member 1 [Source:HGNC Symbol;Acc:HGNC:17845]	-
ENSG00000171385	KCND3	1	111770662	111989155	-	7863	protein_coding	potassium voltage-gated channel subfamily D member 3 [Source:HGNC Symbol;Acc:HGNC:6239]	-
ENSG00000238271	IFNWP19	9	21455484	21456049	+	566	unprocessed_pseudogene	interferon omega 1 pseudogene 19 [Source:HGNC Symbol;Acc:HGNC:5451]	-
ENSG00000081052	COL4A4	2	227002711	227164453	-	10076	protein_coding	collagen type IV alpha 4 chain [Source:HGNC Symbol;Acc:HGNC:2206]	-
ENSG00000168398	BDKRB2	14	96204679	96244166	+	6845	protein_coding	bradykinin receptor B2 [Source:HGNC Symbol;Acc:HGNC:1030]	-
ENSG00000166448	TMEM130	7	98846488	98870771	-	4663	protein_coding	transmembrane protein 130 [Source:HGNC Symbol;Acc:HGNC:25429]	-
ENSG00000113578	FGF1	5	142592178	142698070	-	5275	protein_coding	fibroblast growth factor 1 [Source:HGNC Symbol;Acc:HGNC:3665]	-
ENSG00000165879	FRAT1	10	97319267	97321915	+	2649	protein_coding	FRAT1, WNT signaling pathway regulator [Source:HGNC Symbol;Acc:HGNC:3944]	-
ENSG00000146674	IGFBP3	7	45912245	45921874	-	4571	protein_coding	insulin like growth factor binding protein 3 [Source:HGNC Symbol;Acc:HGNC:5472]	-
ENSG00000169891	REPS2	X	16946691	17153280	+	11311	protein_coding	RALBP1 associated Eps domain containing 2 [Source:HGNC Symbol;Acc:HGNC:9963]	-
ENSG00000120937	NPPB	1	11857464	11858931	-	694	protein_coding	natriuretic peptide B [Source:HGNC Symbol;Acc:HGNC:7940]	-
ENSG00000236333	TRHDE-AS1	12	72253508	72274907	-	8781	processed_transcript	TRHDE antisense RNA 1 [Source:HGNC Symbol;Acc:HGNC:27471]	-
ENSG00000160963	COL26A1	7	101362820	101559024	+	3156	protein_coding	collagen type XXVI alpha 1 chain [Source:HGNC Symbol;Acc:HGNC:18038]	-
ENSG00000139946	PELI2	14	56117814	56301526	+	6278	protein_coding	pellino E3 ubiquitin protein ligase family member 2 [Source:HGNC Symbol;Acc:HGNC:8828]	-
ENSG00000164849	GPR146	7	1044576	1059261	+	3160	protein_coding	G protein-coupled receptor 146 [Source:HGNC Symbol;Acc:HGNC:21718]	-
ENSG00000072657	TRHDE	12	72087266	72670757	+	11950	protein_coding	thyrotropin releasing hormone degrading enzyme [Source:HGNC Symbol;Acc:HGNC:30748]	-
ENSG00000101605	MYOM1	18	3066807	3220108	-	6574	protein_coding	myomesin 1 [Source:HGNC Symbol;Acc:HGNC:7613]	-
ENSG00000170379	TCAF2	7	143620952	143730409	+	6409	protein_coding	TRPM8 channel associated factor 2 [Source:HGNC Symbol;Acc:HGNC:26878]	-
ENSG00000141052	MYOCD	17	12665890	12768949	+	8875	protein_coding	myocardin [Source:HGNC Symbol;Acc:HGNC:16067]	-
ENSG00000138623	SEMA7A	15	74409289	74433958	-	3800	protein_coding	semaphorin 7A (John Milton Hagen blood group) [Source:HGNC Symbol;Acc:HGNC:10741]	-
ENSG00000010310	GIPR	19	45668244	45683724	+	4736	protein_coding	gastric inhibitory polypeptide receptor [Source:HGNC Symbol;Acc:HGNC:4271]	-
ENSG00000233818	AP000695.2	21	36445731	36532408	+	1178	antisense	novel transcript	-
ENSG00000176678	FOXL1	16	86576368	86582160	+	3742	protein_coding	forkhead box L1 [Source:HGNC Symbol;Acc:HGNC:3817]	Fork
ENSG00000186479	RGS7BP	5	64506257	64612312	+	4379	protein_coding	regulator of G protein signaling 7 binding protein [Source:HGNC Symbol;Acc:HGNC:23271]	-
ENSG00000149591	TAGLN	11	117199321	117204782	+	3917	protein_coding	transgelin [Source:HGNC Symbol;Acc:HGNC:11553]	-
ENSG00000172061	LRRC15	3	194355247	194369743	-	5940	protein_coding	leucine rich repeat containing 15 [Source:HGNC Symbol;Acc:HGNC:20818]	-
ENSG00000235750	KIAA0040	1	175156987	175192999	-	4783	protein_coding	KIAA0040 [Source:HGNC Symbol;Acc:HGNC:28950]	-
ENSG00000198542	ITGBL1	13	101452593	101720856	+	6174	protein_coding	integrin subunit beta like 1 [Source:HGNC Symbol;Acc:HGNC:6164]	-
ENSG00000169031	COL4A3	2	227164565	227314792	+	9124	protein_coding	collagen type IV alpha 3 chain [Source:HGNC Symbol;Acc:HGNC:2204]	-
ENSG00000100027	YPEL1	22	21697544	21735834	-	4713	protein_coding	yippee like 1 [Source:HGNC Symbol;Acc:HGNC:12845]	-
ENSG00000152822	GRM1	6	146027646	146437598	+	7272	protein_coding	glutamate metabotropic receptor 1 [Source:HGNC Symbol;Acc:HGNC:4593]	-
ENSG00000106366	SERPINE1	7	101127089	101139266	+	3190	protein_coding	serpin family E member 1 [Source:HGNC Symbol;Acc:HGNC:8583]	-
ENSG00000213694	S1PR3	9	88990863	89005010	+	13578	protein_coding	sphingosine-1-phosphate receptor 3 [Source:HGNC Symbol;Acc:HGNC:3167]	-
ENSG00000135318	NT5E	6	85449584	85495791	+	4533	protein_coding	5'-nucleotidase ecto [Source:HGNC Symbol;Acc:HGNC:8021]	-
ENSG00000165072	MAMDC2	9	70043581	70226970	+	3854	protein_coding	MAM domain containing 2 [Source:HGNC Symbol;Acc:HGNC:23673]	-
ENSG00000126561	STAT5A	17	42287547	42311943	+	5754	protein_coding	signal transducer and activator of transcription 5A [Source:HGNC Symbol;Acc:HGNC:11366]	STAT
ENSG00000113083	LOX	5	122063195	122078360	-	5547	protein_coding	lysyl oxidase [Source:HGNC Symbol;Acc:HGNC:6664]	-
ENSG00000005187	ACSM3	16	20610243	20797581	+	7822	protein_coding	acyl-CoA synthetase medium chain family member 3 [Source:HGNC Symbol;Acc:HGNC:10522]	-
ENSG00000118523	CTGF	6	131948176	131951373	-	2339	protein_coding	connective tissue growth factor [Source:HGNC Symbol;Acc:HGNC:2500]	-
ENSG00000111859	NEDD9	6	11183298	11382348	-	10359	protein_coding	neural precursor cell expressed, developmentally down-regulated 9 [Source:HGNC Symbol;Acc:HGNC:7733]	-
ENSG00000249456	AL731577.2	10	124917143	124942881	+	555	sense_overlapping	novel transcript, sense overlapping ZRANB1	-
ENSG00000131738	KRT33B	17	41363494	41369800	-	1607	protein_coding	keratin 33B [Source:HGNC Symbol;Acc:HGNC:6451]	-
ENSG00000165959	CLMN	14	95181940	95319906	-	15701	protein_coding	calmin [Source:HGNC Symbol;Acc:HGNC:19972]	-
ENSG00000187689	AMTN	4	70518540	70532742	+	1037	protein_coding	amelotin [Source:HGNC Symbol;Acc:HGNC:33188]	-
ENSG00000169991	IFFO2	1	18904281	18956686	-	6188	protein_coding	intermediate filament family orphan 2 [Source:HGNC Symbol;Acc:HGNC:27006]	-
ENSG00000140015	KCNH5	14	62699454	63102037	-	12320	protein_coding	potassium voltage-gated channel subfamily H member 5 [Source:HGNC Symbol;Acc:HGNC:6254]	-
ENSG00000260604	AL590004.3	6	3904920	3911979	-	7060	lincRNA	novel transcript	-
ENSG00000079841	RIMS1	6	71886703	72403143	+	12788	protein_coding	regulating synaptic membrane exocytosis 1 [Source:HGNC Symbol;Acc:HGNC:17282]	-
ENSG00000261051	AC107021.2	3	146059585	146061679	-	2095	sense_overlapping	novel transcript, overlapping to PLOD2	-
ENSG00000106688	SLC1A1	9	4490444	4587469	+	3998	protein_coding	solute carrier family 1 member 1 [Source:HGNC Symbol;Acc:HGNC:10939]	-
ENSG00000164694	FNDC1	6	159169397	159272109	+	6854	protein_coding	fibronectin type III domain containing 1 [Source:HGNC Symbol;Acc:HGNC:21184]	-
ENSG00000139211	AMIGO2	12	47075707	47079951	-	3956	protein_coding	adhesion molecule with Ig like domain 2 [Source:HGNC Symbol;Acc:HGNC:24073]	-
ENSG00000146005	PSD2	5	139795821	139844466	+	4513	protein_coding	pleckstrin and Sec7 domain containing 2 [Source:HGNC Symbol;Acc:HGNC:19092]	-
ENSG00000206538	VGLL3	3	86876388	86991119	-	12714	protein_coding	vestigial like family member 3 [Source:HGNC Symbol;Acc:HGNC:24327]	-
ENSG00000166106	ADAMTS15	11	130448974	130476641	+	5673	protein_coding	ADAM metallopeptidase with thrombospondin type 1 motif 15 [Source:HGNC Symbol;Acc:HGNC:16305]	-
ENSG00000171401	KRT13	17	41500981	41505705	-	2913	protein_coding	keratin 13 [Source:HGNC Symbol;Acc:HGNC:6415]	-
ENSG00000186352	ANKRD37	4	185396021	185400628	+	2580	protein_coding	ankyrin repeat domain 37 [Source:HGNC Symbol;Acc:HGNC:29593]	-
ENSG00000011028	MRC2	17	62627401	62693597	+	6860	protein_coding	mannose receptor C type 2 [Source:HGNC Symbol;Acc:HGNC:16875]	-
ENSG00000082497	SERTAD4	1	210232799	210246631	+	5467	protein_coding	SERTA domain containing 4 [Source:HGNC Symbol;Acc:HGNC:25236]	-
ENSG00000118515	SGK1	6	134169246	134318112	-	9992	protein_coding	serum/glucocorticoid regulated kinase 1 [Source:HGNC Symbol;Acc:HGNC:10810]	-
ENSG00000108821	COL1A1	17	50183289	50201632	-	9819	protein_coding	collagen type I alpha 1 chain [Source:HGNC Symbol;Acc:HGNC:2197]	-
ENSG00000049192	ADAMTS6	5	65148736	65481920	-	8567	protein_coding	ADAM metallopeptidase with thrombospondin type 1 motif 6 [Source:HGNC Symbol;Acc:HGNC:222]	-
ENSG00000171219	CDC42BPG	11	64823387	64844569	-	6112	protein_coding	CDC42 binding protein kinase gamma [Source:HGNC Symbol;Acc:HGNC:29829]	-
ENSG00000103647	CORO2B	15	68578969	68727806	+	3998	protein_coding	coronin 2B [Source:HGNC Symbol;Acc:HGNC:2256]	-
ENSG00000166482	MFAP4	17	19383442	19387240	-	2152	protein_coding	microfibril associated protein 4 [Source:HGNC Symbol;Acc:HGNC:7035]	-
ENSG00000123843	C4BPB	1	207088842	207099993	+	3219	protein_coding	complement component 4 binding protein beta [Source:HGNC Symbol;Acc:HGNC:1328]	-
ENSG00000138356	AOX1	2	200585868	200677064	+	5556	protein_coding	aldehyde oxidase 1 [Source:HGNC Symbol;Acc:HGNC:553]	-
ENSG00000159263	SIM2	21	36699133	36749917	+	7652	protein_coding	SIM bHLH transcription factor 2 [Source:HGNC Symbol;Acc:HGNC:10883]	Others
ENSG00000166145	SPINT1	15	40844018	40858207	+	3542	protein_coding	serine peptidase inhibitor, Kunitz type 1 [Source:HGNC Symbol;Acc:HGNC:11246]	-
ENSG00000113389	NPR3	5	32689070	32791724	+	9866	protein_coding	natriuretic peptide receptor 3 [Source:HGNC Symbol;Acc:HGNC:7945]	-
ENSG00000115380	EFEMP1	2	55865967	55924139	-	3962	protein_coding	EGF containing fibulin extracellular matrix protein 1 [Source:HGNC Symbol;Acc:HGNC:3218]	-
ENSG00000101333	PLCB4	20	9068763	9481242	+	6900	protein_coding	phospholipase C beta 4 [Source:HGNC Symbol;Acc:HGNC:9059]	-
ENSG00000079931	MOXD1	6	132296055	132401545	-	5293	protein_coding	monooxygenase DBH like 1 [Source:HGNC Symbol;Acc:HGNC:21063]	-
ENSG00000119681	LTBP2	14	74498170	74612378	-	9091	protein_coding	latent transforming growth factor beta binding protein 2 [Source:HGNC Symbol;Acc:HGNC:6715]	-
ENSG00000146094	DOK3	5	177501907	177511274	-	4823	protein_coding	docking protein 3 [Source:HGNC Symbol;Acc:HGNC:24583]	-
ENSG00000243753	HLA-L	6	30259584	30293014	+	5505	transcribed_unprocessed_pseudogene	major histocompatibility complex, class I, L (pseudogene) [Source:HGNC Symbol;Acc:HGNC:4970]	-
ENSG00000027075	PRKCH	14	61187559	61550976	+	8833	protein_coding	protein kinase C eta [Source:HGNC Symbol;Acc:HGNC:9403]	-
ENSG00000175274	TP53I11	11	44885903	44951306	-	7345	protein_coding	tumor protein p53 inducible protein 11 [Source:HGNC Symbol;Acc:HGNC:16842]	-
ENSG00000162373	BEND5	1	48727523	48776969	-	2138	protein_coding	BEN domain containing 5 [Source:HGNC Symbol;Acc:HGNC:25668]	-
ENSG00000074590	NUAK1	12	106063340	106140033	-	7008	protein_coding	NUAK family kinase 1 [Source:HGNC Symbol;Acc:HGNC:14311]	-
ENSG00000153162	BMP6	6	7726797	7881422	+	2780	protein_coding	bone morphogenetic protein 6 [Source:HGNC Symbol;Acc:HGNC:1073]	-
ENSG00000004799	PDK4	7	95583499	95596491	-	4519	protein_coding	pyruvate dehydrogenase kinase 4 [Source:HGNC Symbol;Acc:HGNC:8812]	-
ENSG00000175161	CADM2	3	84958981	86074429	+	9990	protein_coding	cell adhesion molecule 2 [Source:HGNC Symbol;Acc:HGNC:29849]	-
ENSG00000141738	GRB7	17	39737927	39747291	+	3597	protein_coding	growth factor receptor bound protein 7 [Source:HGNC Symbol;Acc:HGNC:4567]	-
ENSG00000198948	MFAP3L	4	169986597	170033031	-	8741	protein_coding	microfibril associated protein 3 like [Source:HGNC Symbol;Acc:HGNC:29083]	-
ENSG00000177181	RIMKLA	1	42380795	42422578	+	8981	protein_coding	ribosomal modification protein rimK like family member A [Source:HGNC Symbol;Acc:HGNC:28725]	-
ENSG00000157680	DGKI	7	137381037	137847092	-	15549	protein_coding	diacylglycerol kinase iota [Source:HGNC Symbol;Acc:HGNC:2855]	-
ENSG00000075618	FSCN1	7	5592823	5606655	+	3692	protein_coding	fascin actin-bundling protein 1 [Source:HGNC Symbol;Acc:HGNC:11148]	-
ENSG00000187498	COL4A1	13	110148963	110307233	-	14738	protein_coding	collagen type IV alpha 1 chain [Source:HGNC Symbol;Acc:HGNC:2202]	-
ENSG00000257671	KRT7-AS	12	52245048	52274448	-	1693	antisense	KRT7 antisense RNA 1 [Source:HGNC Symbol;Acc:HGNC:52643]	-
ENSG00000144583	4-Mar	2	216257865	216372027	-	4447	protein_coding	membrane associated ring-CH-type finger 4 [Source:HGNC Symbol;Acc:HGNC:29269]	-
ENSG00000115414	FN1	2	215360440	215436172	-	17315	protein_coding	fibronectin 1 [Source:HGNC Symbol;Acc:HGNC:3778]	-
ENSG00000058335	RASGRF1	15	78959947	79090773	-	9929	protein_coding	Ras protein specific guanine nucleotide releasing factor 1 [Source:HGNC Symbol;Acc:HGNC:9875]	-
ENSG00000108846	ABCC3	17	50634777	50692252	+	9614	protein_coding	ATP binding cassette subfamily C member 3 [Source:HGNC Symbol;Acc:HGNC:54]	-
ENSG00000185100	ADSSL1	14	104724186	104747325	+	5775	protein_coding	adenylosuccinate synthase like 1 [Source:HGNC Symbol;Acc:HGNC:20093]	-
ENSG00000079308	TNS1	2	217799588	218033982	-	16281	protein_coding	tensin 1 [Source:HGNC Symbol;Acc:HGNC:11973]	-
ENSG00000020181	ADGRA2	8	37784191	37844896	+	7061	protein_coding	adhesion G protein-coupled receptor A2 [Source:HGNC Symbol;Acc:HGNC:17849]	-
ENSG00000073464	CLCN4	X	10156945	10237660	+	6789	protein_coding	chloride voltage-gated channel 4 [Source:HGNC Symbol;Acc:HGNC:2022]	-
ENSG00000136490	LIMD2	17	63695902	63701172	-	4745	protein_coding	LIM domain containing 2 [Source:HGNC Symbol;Acc:HGNC:28142]	-
ENSG00000214517	PPME1	11	74171099	74254703	+	7917	protein_coding	protein phosphatase methylesterase 1 [Source:HGNC Symbol;Acc:HGNC:30178]	-
ENSG00000198053	SIRPA	20	1894167	1940592	+	5068	protein_coding	signal regulatory protein alpha [Source:HGNC Symbol;Acc:HGNC:9662]	-
ENSG00000181274	FRAT2	10	97332497	97334709	-	2213	protein_coding	FRAT2, WNT signaling pathway regulator [Source:HGNC Symbol;Acc:HGNC:16048]	-
ENSG00000112139	MDGA1	6	37630679	37699306	-	14187	protein_coding	MAM domain containing glycosylphosphatidylinositol anchor 1 [Source:HGNC Symbol;Acc:HGNC:19267]	-
ENSG00000261068	AL512274.1	6	42092233	42094259	-	2027	lincRNA	novel transcript	-
ENSG00000144642	RBMS3	3	28574791	30010391	+	18339	protein_coding	RNA binding motif single stranded interacting protein 3 [Source:HGNC Symbol;Acc:HGNC:13427]	-
ENSG00000235609	AF127577.4	21	14818843	15014430	-	5929	lincRNA	novel transcript	-
ENSG00000125730	C3	19	6677704	6730562	-	8328	protein_coding	complement C3 [Source:HGNC Symbol;Acc:HGNC:1318]	-
ENSG00000105856	HBP1	7	107168961	107202529	+	6351	protein_coding	HMG-box transcription factor 1 [Source:HGNC Symbol;Acc:HGNC:23200]	HMG
ENSG00000133424	LARGE1	22	33162226	33922841	-	10687	protein_coding	LARGE xylosyl- and glucuronyltransferase 1 [Source:HGNC Symbol;Acc:HGNC:6511]	-
ENSG00000164176	EDIL3	5	83940554	84384793	-	5738	protein_coding	EGF like repeats and discoidin domains 3 [Source:HGNC Symbol;Acc:HGNC:3173]	-
ENSG00000054277	OPN3	1	241590102	241677376	-	3716	protein_coding	opsin 3 [Source:HGNC Symbol;Acc:HGNC:14007]	-
ENSG00000185499	MUC1	1	155185824	155192916	-	4717	protein_coding	mucin 1, cell surface associated [Source:HGNC Symbol;Acc:HGNC:7508]	-
ENSG00000213713	PIGCP1	11	33075566	33076460	-	895	processed_pseudogene	phosphatidylinositol glycan anchor biosynthesis class C pseudogene 1 [Source:HGNC Symbol;Acc:HGNC:8961]	-
ENSG00000164197	RNF180	5	64165844	64372869	+	5434	protein_coding	ring finger protein 180 [Source:HGNC Symbol;Acc:HGNC:27752]	-
ENSG00000001617	SEMA3F	3	50155045	50189075	+	4826	protein_coding	semaphorin 3F [Source:HGNC Symbol;Acc:HGNC:10728]	-
ENSG00000114796	KLHL24	3	183635568	183684477	+	8650	protein_coding	kelch like family member 24 [Source:HGNC Symbol;Acc:HGNC:25947]	-
ENSG00000133216	EPHB2	1	22710839	22921500	+	12516	protein_coding	EPH receptor B2 [Source:HGNC Symbol;Acc:HGNC:3393]	-
ENSG00000152409	JMY	5	79236189	79327215	+	9042	protein_coding	junction mediating and regulatory protein, p53 cofactor [Source:HGNC Symbol;Acc:HGNC:28916]	-
ENSG00000165312	OTUD1	10	23439458	23442390	+	2933	protein_coding	OTU deubiquitinase 1 [Source:HGNC Symbol;Acc:HGNC:27346]	-
ENSG00000108379	WNT3	17	46762506	46833154	-	4334	protein_coding	Wnt family member 3 [Source:HGNC Symbol;Acc:HGNC:12782]	-
ENSG00000170442	KRT86	12	52249300	52309163	+	2394	protein_coding	keratin 86 [Source:HGNC Symbol;Acc:HGNC:6463]	-
ENSG00000168453	HR	8	22114415	22133384	-	8784	protein_coding	HR, lysine demethylase and nuclear receptor corepressor [Source:HGNC Symbol;Acc:HGNC:5172]	-
ENSG00000278948	AL031587.5	22	37943050	37944898	+	1849	TEC	TEC	-
ENSG00000133107	TRPC4	13	37636636	37870425	-	4205	protein_coding	transient receptor potential cation channel subfamily C member 4 [Source:HGNC Symbol;Acc:HGNC:12336]	-
ENSG00000102452	NALCN	13	101053776	101416492	-	9140	protein_coding	sodium leak channel, non-selective [Source:HGNC Symbol;Acc:HGNC:19082]	-
ENSG00000154736	ADAMTS5	21	26917912	26966513	-	9056	protein_coding	ADAM metallopeptidase with thrombospondin type 1 motif 5 [Source:HGNC Symbol;Acc:HGNC:221]	-
ENSG00000173801	JUP	17	41754604	41786931	-	4942	protein_coding	junction plakoglobin [Source:HGNC Symbol;Acc:HGNC:6207]	-
ENSG00000151414	NEK7	1	198156963	198322420	+	5485	protein_coding	NIMA related kinase 7 [Source:HGNC Symbol;Acc:HGNC:13386]	-
ENSG00000073910	FRY	13	31846713	32299122	+	18849	protein_coding	FRY microtubule binding protein [Source:HGNC Symbol;Acc:HGNC:20367]	-
ENSG00000011201	ANOS1	X	8528874	8732186	-	7131	protein_coding	anosmin 1 [Source:HGNC Symbol;Acc:HGNC:6211]	-
ENSG00000091592	NLRP1	17	5499427	5619424	-	12808	protein_coding	NLR family pyrin domain containing 1 [Source:HGNC Symbol;Acc:HGNC:14374]	-
ENSG00000127329	PTPRB	12	70515866	70637440	-	14542	protein_coding	protein tyrosine phosphatase, receptor type B [Source:HGNC Symbol;Acc:HGNC:9665]	-
ENSG00000122707	RECK	9	36036433	36124451	+	6045	protein_coding	reversion inducing cysteine rich protein with kazal motifs [Source:HGNC Symbol;Acc:HGNC:11345]	-
ENSG00000100302	RASD2	22	35540868	35554001	+	3412	protein_coding	RASD family member 2 [Source:HGNC Symbol;Acc:HGNC:18229]	-
ENSG00000112414	ADGRG6	6	142301854	142446266	+	8473	protein_coding	adhesion G protein-coupled receptor G6 [Source:HGNC Symbol;Acc:HGNC:13841]	-
ENSG00000169432	SCN9A	2	166195185	166376001	-	13741	protein_coding	sodium voltage-gated channel alpha subunit 9 [Source:HGNC Symbol;Acc:HGNC:10597]	-
ENSG00000166046	TCP11L2	12	106301929	106347015	+	6558	protein_coding	t-complex 11 like 2 [Source:HGNC Symbol;Acc:HGNC:28627]	-
ENSG00000173894	CBX2	17	79778132	79787983	+	5687	protein_coding	chromobox 2 [Source:HGNC Symbol;Acc:HGNC:1552]	-
ENSG00000132434	LANCL2	7	55365448	55433742	+	5235	protein_coding	LanC like 2 [Source:HGNC Symbol;Acc:HGNC:6509]	-
ENSG00000092295	TGM1	14	24249114	24264432	-	3735	protein_coding	transglutaminase 1 [Source:HGNC Symbol;Acc:HGNC:11777]	-
ENSG00000211455	STK38L	12	27243968	27325959	+	6921	protein_coding	serine/threonine kinase 38 like [Source:HGNC Symbol;Acc:HGNC:17848]	-
ENSG00000095951	HIVEP1	6	12008762	12164999	+	10979	protein_coding	human immunodeficiency virus type I enhancer binding protein 1 [Source:HGNC Symbol;Acc:HGNC:4920]	zf-C2H2
ENSG00000120162	MOB3B	9	27325209	27529781	-	7087	protein_coding	MOB kinase activator 3B [Source:HGNC Symbol;Acc:HGNC:23825]	-
ENSG00000100311	PDGFB	22	39223359	39244751	-	3761	protein_coding	platelet derived growth factor subunit B [Source:HGNC Symbol;Acc:HGNC:8800]	-
ENSG00000135245	HILPDA	7	128455849	128458418	+	2070	protein_coding	hypoxia inducible lipid droplet associated [Source:HGNC Symbol;Acc:HGNC:28859]	-
ENSG00000152779	SLC16A12	10	89430299	89556641	-	4835	protein_coding	solute carrier family 16 member 12 [Source:HGNC Symbol;Acc:HGNC:23094]	-
ENSG00000091986	CCDC80	3	112596794	112649530	-	13071	protein_coding	coiled-coil domain containing 80 [Source:HGNC Symbol;Acc:HGNC:30649]	-
ENSG00000122786	CALD1	7	134744252	134970728	+	9843	protein_coding	caldesmon 1 [Source:HGNC Symbol;Acc:HGNC:1441]	-
ENSG00000187398	LUZP2	11	24496970	25082631	+	8851	protein_coding	leucine zipper protein 2 [Source:HGNC Symbol;Acc:HGNC:23206]	-
ENSG00000004399	PLXND1	3	129555175	129606818	-	9738	protein_coding	plexin D1 [Source:HGNC Symbol;Acc:HGNC:9107]	-
ENSG00000138641	HERC3	4	88592423	88708542	+	6981	protein_coding	HECT and RLD domain containing E3 ubiquitin protein ligase 3 [Source:HGNC Symbol;Acc:HGNC:4876]	-
ENSG00000130518	IQCN	19	18257097	18274509	-	9154	protein_coding	IQ motif containing N [Source:HGNC Symbol;Acc:HGNC:29350]	-
ENSG00000163235	TGFA	2	70447280	70554193	-	4963	protein_coding	transforming growth factor alpha [Source:HGNC Symbol;Acc:HGNC:11765]	-

gene_id	CH_0323_count	CH_0407_count	CH_0603_count	DMSO_0323_count	DMSO_0407_count	DMSO_0603_count	CH_0323_fpkm	CH_0407_fpkm	CH_0603_fpkm	DMSO_0323_fpkm	DMSO_0407_fpkm	DMSO_0603_fpkm
ENSG00000109193	0	0	0	35	49	10	0	0	0	0.738979616	1.019424413	0.220932552
ENSG00000101203	0	0	0	27	18	23	0	0	0	0.108621865	0.071354363	0.096822573
ENSG00000198959	38	68	128	665	678	300	0.218503043	0.354214461	0.553864636	3.653424736	3.67031001	1.724626551
ENSG00000188869	113	57	68	421	639	645	1.077034594	0.492163664	0.487730479	3.833874853	5.733914719	6.146258823
ENSG00000159251	52	82	144	371	782	560	0.522120265	0.745872407	1.088051551	3.559145503	7.392190003	5.621538041
ENSG00000111186	4	6	11	47	37	38	0.042933931	0.058341205	0.088849119	0.481995684	0.373888021	0.407778367
ENSG00000130720	10	20	11	68	121	42	0.120615096	0.218532053	0.099842197	0.783637496	1.373998317	0.506466681
ENSG00000156466	13	26	8	74	183	13	0.162307313	0.294070572	0.075163068	0.882736496	2.151022667	0.162269915
ENSG00000179772	29	58	29	205	270	113	1.015937574	1.840689356	0.764516384	6.861633433	8.904959548	3.957741175
ENSG00000172575	147	144	243	1016	767	817	1.114076364	0.988652343	1.385873764	7.356918764	5.472580408	6.190412691
ENSG00000259207	65	38	159	415	346	410	0.683545022	0.362010233	1.258260799	4.16971615	3.425539885	4.310598221
ENSG00000186994	10	7	9	36	54	30	0.128568847	0.081529968	0.087075912	0.442224577	0.653625022	0.385617667
ENSG00000173597	15	36	13	133	96	57	0.094224395	0.204860431	0.061451887	0.798230592	0.567730851	0.357970202
ENSG00000171385	83	49	137	387	399	323	0.487757142	0.260858569	0.60585108	2.17290699	2.207484193	1.89770188
ENSG00000238271	10	19	10	47	72	43	0.816389461	1.40518813	0.61435184	3.666062582	5.53387121	3.509665815
ENSG00000081052	2379	1650	2243	9601	9314	6635	10.90988327	6.854773171	7.740601495	42.06750534	40.21249845	30.42051132
ENSG00000168398	15	19	9	72	39	65	0.101258532	0.116192328	0.045719624	0.46438426	0.247858683	0.438685869
ENSG00000166448	12	10	32	25	93	88	0.118913086	0.089770137	0.238626218	0.236697157	0.86762192	0.871828371
ENSG00000113578	48	21	19	111	144	109	0.420467656	0.166645708	0.12524625	0.929006949	1.187553026	0.954591967
ENSG00000165879	25	5	21	59	83	62	0.436085725	0.079010598	0.27565821	0.983305574	1.363041618	1.081243407
ENSG00000146674	9480	11744	8833	41875	52030	26868	95.8320849	107.5479469	67.19401684	404.4473986	495.1714389	271.5425297
ENSG00000169891	44	34	73	232	198	152	0.179748591	0.125827398	0.224416845	0.905535394	0.761512734	0.620806602
ENSG00000120937	1184	2062	1153	3798	7658	5939	78.83263669	124.3731098	57.77014154	241.6094616	480.030546	395.3371218
ENSG00000236333	9	4	36	48	55	73	0.047360072	0.019068359	0.142558172	0.241332442	0.272478158	0.384054293
ENSG00000160963	15	11	12	53	62	32	0.219618077	0.145899228	0.132214122	0.741408094	0.854609423	0.468410611
ENSG00000139946	65	58	41	328	204	97	0.478416188	0.386726547	0.227089022	2.306593055	1.413584708	0.713779654
ENSG00000164849	13	14	22	54	90	36	0.190094736	0.185454876	0.242085731	0.754440727	1.238991735	0.526294897
ENSG00000072657	64	55	159	238	257	480	0.247471898	0.192660236	0.462660916	0.879280617	0.935574121	1.855611577
ENSG00000101605	8	14	15	34	52	48	0.05623078	0.089144723	0.07934054	0.228332465	0.344101548	0.337306942
ENSG00000170379	44	26	36	197	132	56	0.317231442	0.169816693	0.195319599	1.357045478	0.895976547	0.403656078
ENSG00000141052	42	15	74	81	188	180	0.218672792	0.070748983	0.289932535	0.402935104	0.92151513	0.936953169
ENSG00000138623	235	247	256	833	954	815	2.857577951	2.720887964	2.342555901	9.677871523	10.92138609	9.908040049
ENSG00000010310	12	5	33	38	70	59	0.117080178	0.044193217	0.242290196	0.354234088	0.64298276	0.57551157
ENSG00000233818	15	33	16	107	75	45	0.588382557	1.17264337	0.472289496	4.010115994	2.769675921	1.764740955
ENSG00000176678	14	13	46	71	86	83	0.172877341	0.145424263	0.427452285	0.837670099	0.99978738	1.024679507
ENSG00000186479	958	438	1436	3714	2660	3006	10.10891127	4.186937405	11.40284155	37.4442729	26.42528463	31.71230227
ENSG00000149591	905	1560	1063	3218	5644	3166	10.67600647	16.67125635	9.43655092	36.27028063	62.68251538	37.339722
ENSG00000172061	7	15	39	41	86	59	0.054453452	0.105706603	0.228303073	0.304730454	0.629832386	0.458859057
ENSG00000235750	9	15	24	44	64	42	0.08694727	0.13127686	0.174479519	0.406135304	0.582093266	0.405660434
ENSG00000198542	48	58	80	222	270	100	0.359243098	0.393240891	0.450564485	1.587467332	1.902436288	0.748250673
ENSG00000169031	1072	1110	794	3475	3887	2397	5.429043599	5.092546522	3.025999281	16.81465574	18.53285152	12.13658491
ENSG00000100027	137	49	104	396	300	232	1.343188448	0.435207071	0.76730759	3.70950699	2.769088253	2.274072395
ENSG00000152822	26	39	36	112	146	59	0.165208846	0.224495707	0.172140169	0.679958802	0.873397547	0.374810616
ENSG00000106366	5403	6477	9026	17642	26503	20124	78.2632908	84.99248297	98.38711837	244.1605993	361.4242894	291.4320873
ENSG00000213694	21	12	22	48	69	54	0.071465644	0.036994976	0.056340471	0.156071599	0.221068197	0.183726456
ENSG00000135318	668	551	1141	2732	2118	2283	6.809332856	5.08818839	8.752528228	26.60807301	20.32606074	23.26665412
ENSG00000165072	790	1140	1026	2764	4139	2173	9.471727644	12.38198986	9.25697829	31.66246889	46.71933995	26.04724273
ENSG00000126561	42	8	41	72	73	131	0.337282069	0.058199256	0.247769357	0.552434874	0.551907105	1.051756439
ENSG00000113083	9085	9094	7962	32068	30645	16946	75.67990642	68.62685345	49.91115294	255.2302564	240.3335725	141.1311165
ENSG00000005187	32	37	36	145	138	33	0.189036639	0.198007306	0.16003622	0.818405686	0.767492707	0.194899116
ENSG00000118523	10448	12822	13618	32703	44760	31078	206.40336	229.4683821	202.4494973	617.2709237	832.4781404	613.8137062
ENSG00000111859	623	605	957	2245	2047	2013	2.778971125	2.444752194	3.212385814	9.567907321	8.596328276	8.977174829
ENSG00000249456	29	14	53	55	114	102	2.414453442	1.055923257	3.32059937	4.375101713	8.935623273	8.490258825
ENSG00000131738	1263	1425	2569	3878	5938	4975	36.31627486	37.11900195	55.58809899	106.5394316	160.7449355	143.018082
ENSG00000165959	64	8	69	134	146	121	0.188350371	0.021328483	0.152811265	0.376787019	0.404518627	0.35601787
ENSG00000187689	555	1242	813	2028	3457	1922	24.73022384	50.13489877	27.26122605	86.33912478	145.0219049	85.62259148
ENSG00000169991	599	458	657	1354	1895	1600	4.472911835	3.098221589	3.691889205	9.660215326	13.32207553	11.94492477
ENSG00000140015	31	49	95	171	147	153	0.116269233	0.1664879	0.268130669	0.612778967	0.519062446	0.573712701
ENSG00000260604	466	409	642	1587	1371	1192	3.049966269	2.425023267	3.16201497	9.924089524	8.447841094	7.799832845
ENSG00000079841	121	220	414	490	768	720	0.43721652	0.720140699	1.125722401	1.691655403	2.612591893	2.601019512
ENSG00000261051	29	28	24	100	68	54	0.639628478	0.559462919	0.39834632	2.107339076	1.41201031	1.190757906
ENSG00000106688	159	151	154	456	454	349	1.837672664	1.580998509	1.339403797	5.035475654	4.939989723	4.032704301
ENSG00000164694	92	100	54	237	347	99	0.620236825	0.610735553	0.273957538	1.526590986	2.202410947	0.667274972
ENSG00000139211	147	149	335	530	675	416	1.717018096	1.576620933	2.944571598	5.914772354	7.422675458	4.857924814
ENSG00000146005	23	23	38	90	58	70	0.235492089	0.213333867	0.292787046	0.880431604	0.55908217	0.716549913
ENSG00000206538	5819	4427	6839	16926	13885	14122	21.14851953	14.5755388	18.70440904	58.77472111	47.50908425	51.31303958
ENSG00000166106	669	497	688	1521	1825	1520	5.449129822	3.6672533	4.217054846	11.83681549	13.99468307	12.37783091
ENSG00000171401	80	59	100	168	283	166	1.26900497	0.847830098	1.193694273	2.546169108	4.226286488	2.632578588
ENSG00000186352	64	139	130	344	273	229	1.146236117	2.255238086	1.752093349	5.886500486	4.603158439	4.100431089
ENSG00000011028	268	144	335	599	652	652	1.805196567	0.878689991	1.698064904	3.854971346	4.1346282	4.390734949
ENSG00000082497	289	276	376	817	817	764	2.442657575	2.113281305	2.391510906	6.597682775	6.501086806	6.455918303
ENSG00000118515	217	369	392	675	827	898	1.00350867	1.545863858	1.364166048	2.982426813	3.600526955	4.151811739
ENSG00000108821	1670	3001	1774	4288	8031	3916	7.858922965	12.79369633	6.282318497	19.27995271	35.58077048	18.42422227
ENSG00000049192	26	25	39	63	78	77	0.14023564	0.12215424	0.158295816	0.324661081	0.396076265	0.415217548
ENSG00000171219	35	44	101	113	133	176	0.264605288	0.301346835	0.57460794	0.816231866	0.94663221	1.330280005
ENSG00000103647	60	55	46	175	147	80	0.693461383	0.575860384	0.400081654	1.932474209	1.599512091	0.924402132
ENSG00000166482	84	81	61	146	260	152	1.803644076	1.575578532	0.985646452	2.995222134	5.255863327	3.262984886
ENSG00000123843	29	93	58	140	177	115	0.416285076	1.209370233	0.626528183	1.920107335	2.392022564	1.650405282
ENSG00000138356	277	251	547	927	839	750	2.303728805	1.891075148	3.42340818	7.366071748	6.569203357	6.236095646
ENSG00000159263	74	50	90	227	190	93	0.446859072	0.273522052	0.408979126	1.309692509	1.080168783	0.561463758
ENSG00000166145	45	62	69	101	121	185	0.58705363	0.732724031	0.677382743	1.25890009	1.486106028	2.412886607
ENSG00000113389	1517	1111	2316	3909	3313	4066	7.104905244	4.713790217	8.162647435	17.49214251	14.60808567	19.03881897
ENSG00000115380	10425	10538	9120	29961	26814	14623	121.5837161	111.3373874	80.04126833	333.856842	294.4151243	170.5044625
ENSG00000101333	5133	4007	7055	14830	11220	11091	34.37446868	24.3090258	35.55343136	94.88782849	70.73864694	74.25665054
ENSG00000079931	91	101	167	320	297	196	0.794425761	0.798760872	1.097104943	2.669110366	2.440998641	1.710676615
ENSG00000119681	4637	4023	5632	10474	12375	10014	23.56889701	18.52403862	21.54192865	50.86503638	59.21701779	50.8873307
ENSG00000146094	104	93	99	274	288	123	0.996391234	0.807166241	0.713758885	2.508139851	2.597695298	1.178152721
ENSG00000243753	59	75	44	149	197	69	0.495231783	0.570297205	0.277925853	1.194943559	1.556760792	0.579035924
ENSG00000027075	70	69	94	182	201	142	0.366187597	0.32699278	0.370043873	0.909665251	0.98992162	0.742666536
ENSG00000175274	924	811	597	1734	2245	1410	5.812915257	4.621961855	2.82628612	10.42259208	13.29650581	8.868313837
ENSG00000162373	107	84	81	231	224	165	2.312543429	1.644632575	1.317379536	4.770047752	4.557779386	3.565249967
ENSG00000074590	440	299	678	926	1129	1038	2.90116483	1.785970981	3.364102312	5.83358246	7.008302372	6.842534591
ENSG00000153162	2006	1786	1660	4725	4664	2996	33.3426377	26.89267241	20.76332428	75.03700036	72.9838431	49.78640347
ENSG00000004799	55	78	79	231	122	116	0.56238557	0.722519486	0.607880686	2.256774086	1.174439019	1.185848993
ENSG00000175161	161	253	199	584	430	342	0.744687748	1.060113429	0.692661713	2.580868081	1.872474662	1.581518801
ENSG00000141738	188	156	142	245	547	293	2.415078391	1.815438175	1.372718546	3.007073852	6.615457304	3.763058101
ENSG00000198948	304	325	444	723	808	775	1.60703851	1.556393985	1.766263298	3.651704483	4.021269898	4.095946954
ENSG00000177181	679	295	564	1553	1065	731	3.493485126	1.374974432	2.183675001	7.634229419	5.158671744	3.760160837
ENSG00000157680	36	49	104	100	161	127	0.106982775	0.131914009	0.232575772	0.283933074	0.450439424	0.377324494
ENSG00000075618	769	1209	1431	1561	2454	3072	9.624506453	13.70761542	13.47756814	18.6663609	28.91517655	38.43910439
ENSG00000187498	10573	9201	11852	20359	27350	19771	33.14923425	26.13327156	27.96318818	60.98686901	80.72940672	61.97318624
ENSG00000257671	127	133	158	299	361	229	3.466255594	3.288455625	3.245142137	7.797092345	9.276053102	6.248737277
ENSG00000144583	174	194	310	309	581	509	1.807989648	1.826130892	2.423975127	3.067678182	5.683580104	5.287670619
ENSG00000115414	7924	4852	10026	18783	15940	13018	21.14636828	11.72993483	20.13440495	47.89177244	40.04781292	34.73245747
ENSG00000058335	45	69	78	146	126	127	0.209421287	0.290898099	0.273163511	0.649180988	0.552049495	0.590897226
ENSG00000108846	60	61	112	120	242	117	0.288377222	0.265596911	0.405086248	0.551055798	1.095025494	0.562206012
ENSG00000185100	125	231	223	400	443	352	1.000165443	1.674392593	1.342723127	3.057922331	3.337065415	2.815816932
ENSG00000079308	722	327	570	1207	1203	1025	2.04913203	0.840744392	1.217383396	3.272989721	3.21438234	2.908416035
ENSG00000020181	79	66	127	175	189	187	0.516981141	0.391268628	0.625419048	1.094183811	1.164417101	1.223458201
ENSG00000073464	158	147	256	404	399	336	1.075387784	0.906376901	1.31119641	2.627205255	2.556701754	2.286373669
ENSG00000136490	46	84	64	93	180	126	0.44795608	0.741037818	0.46900487	0.865296963	1.650248211	1.226727411
ENSG00000214517	2945	3721	4429	6908	8485	7181	17.18851964	19.67416584	19.4526436	38.52211573	46.62342608	41.90231555
ENSG00000198053	2138	1975	2982	4108	5297	4983	19.49327974	16.31277314	20.45995281	35.78592739	45.46805211	45.42218504
ENSG00000181274	61	105	102	171	220	151	1.273685608	1.986118643	1.602700427	3.41140392	4.324682703	3.15216741
ENSG00000112139	5104	4963	5265	8817	12476	10161	16.62393827	14.64370628	12.90450652	27.43776422	38.25584329	33.08717008
ENSG00000261068	43	73	65	136	94	135	0.980231213	1.507531565	1.115047075	2.96212654	2.017377103	3.076760994
ENSG00000144642	773	584	870	1819	1431	1272	1.947680266	1.333013352	1.649594488	4.379005556	3.394509009	3.204241213
ENSG00000235609	125	129	187	347	238	297	0.97418712	0.910763386	1.096714918	2.583845086	1.746258323	2.314135263
ENSG00000125730	128	62	205	241	231	306	0.710203934	0.31163647	0.855946734	1.277599619	1.206658137	1.697440075
ENSG00000105856	902	534	1087	2002	1677	1403	6.562634925	3.519625432	5.951425837	13.91683275	11.48692888	10.2053828
ENSG00000133424	402	297	611	788	1043	777	1.738137239	1.163316647	1.988011973	3.255283791	4.245623528	3.358759831
ENSG00000164176	1324	1093	1374	3255	2494	1968	10.66206343	7.973645453	8.32644818	25.04429995	18.90814341	15.84449097
ENSG00000054277	222	179	234	441	496	353	2.760521219	2.016390435	2.189645187	5.239397298	5.806560475	4.388466034
ENSG00000185499	343	254	289	715	740	371	3.360021562	2.254058293	2.130421622	6.69204131	6.824625521	3.633471639
ENSG00000213713	97	55	123	260	140	150	5.007979236	2.572390856	4.778764963	12.82533626	6.804841011	7.742513388
ENSG00000164197	417	356	522	1052	807	739	3.545930682	2.742380213	3.340292233	8.547016717	6.460511189	6.282587495
ENSG00000001617	145	106	119	227	282	246	1.388335744	0.919424031	0.857419268	2.076619784	2.541995475	2.354840686
ENSG00000114796	1160	1162	1614	3195	2217	2419	6.196631956	5.623249114	6.488152029	16.30696739	11.14968423	12.91913695
ENSG00000133216	738	559	814	1362	1817	1070	2.724611767	1.86957786	2.261478406	4.804298695	6.315421702	3.949407663
ENSG00000152409	752	471	1058	1712	1329	1431	3.842971454	2.180488032	4.068691482	8.359065057	6.3940122	7.311203501
ENSG00000165312	750	630	945	1095	1458	2005	11.815797	8.991368341	11.20349024	16.48240206	21.62511588	31.58028574
ENSG00000108379	60	70	166	166	173	220	0.639699725	0.676092995	1.331842213	1.69097672	1.736481571	2.345025205
ENSG00000170442	524	498	298	800	1099	784	10.11395371	8.70768078	4.3283833	14.75313405	19.97038919	15.12884097
ENSG00000168453	345	268	343	343	728	809	1.814849385	1.277143713	1.357798697	1.723932434	3.605388213	4.254709723
ENSG00000278948	58	143	230	238	317	253	1.449455555	3.237400498	4.325382508	5.682749253	7.458222465	6.321168267
ENSG00000133107	452	324	594	1100	741	794	4.966909595	3.225346018	4.911951156	11.54901998	7.665936018	8.723047624
ENSG00000102452	171	185	242	470	406	278	0.864497487	0.847271963	0.920667399	2.270231315	1.932381274	1.405116525
ENSG00000154736	2998	2413	5781	6239	6436	8093	15.29709752	11.15368079	22.19729993	30.41564422	30.91666242	41.28448466
ENSG00000173801	1914	1611	1801	3582	3693	3065	17.89587811	13.64552038	12.67198256	31.99935969	32.50794744	28.65111178
ENSG00000151414	2039	1247	2588	4759	3439	2997	17.17728077	9.516716335	16.40669992	38.30518115	27.27523658	25.24200522
ENSG00000073910	210	161	332	505	404	415	0.514807424	0.357548421	0.612467945	1.182827768	0.93240809	1.017123114
ENSG00000011201	1604	1238	1807	3579	3050	2304	10.39364186	7.26720667	8.811326839	22.15795671	18.60640137	14.9260805
ENSG00000091592	3930	4519	4630	5811	8979	10166	14.17832908	14.76924604	12.5699418	20.03032538	30.49717663	36.6676036
ENSG00000127329	587	440	1154	1561	1264	1205	1.865210199	1.266560207	2.759403833	4.739114595	3.781254562	3.828041593
ENSG00000122707	871	659	1028	1746	1612	1503	6.65787551	4.563377663	5.913306693	12.75164994	11.60063372	11.48620113
ENSG00000100302	70	120	173	218	295	161	0.947987996	1.472209197	1.763074546	2.820758586	3.761196089	2.179870001
ENSG00000112414	276	211	675	810	552	744	1.505170494	1.04241956	2.770130067	4.220522891	2.834097935	4.056481226
ENSG00000169432	552	295	503	1054	873	648	1.856241845	0.898671521	1.272867624	3.386419209	2.763814471	2.178564425
ENSG00000166046	296	289	442	812	509	585	2.085614893	1.844691443	2.343605193	5.466420854	3.376442455	4.120958063
ENSG00000173894	98	123	121	225	241	177	0.796263242	0.905355587	0.739836471	1.746697656	1.843515514	1.437817547
ENSG00000132434	777	822	871	1490	1868	1297	6.858326452	6.572830524	5.78542228	12.56573886	15.52291103	11.4455596
ENSG00000092295	889	1061	1038	1721	2108	1771	10.99828515	11.89110135	9.663631084	20.3427055	24.55234527	21.90492125
ENSG00000211455	1236	1275	1735	2981	2664	2233	8.252080238	7.711496014	8.716943371	19.01566748	16.74473788	14.9050561
ENSG00000095951	847	742	1569	2135	1735	1866	3.564794063	2.829035669	4.96928326	8.585261776	6.8746355	7.851680076
ENSG00000120162	64	128	147	249	190	179	0.417283644	0.756040115	0.721254435	1.551155589	1.166283551	1.166821276
ENSG00000100311	178	203	225	373	448	303	2.18690788	2.259383784	2.080236822	4.378485805	5.181883716	3.721800041
ENSG00000135245	286	273	231	549	615	330	6.38424446	5.520642246	3.880388682	11.70901727	12.92461909	7.36473858
ENSG00000152779	349	299	706	709	754	947	3.335360407	2.588642117	5.077405129	6.473933058	6.784031407	9.048305218
ENSG00000091986	26909	13442	21531	46249	41146	28654	95.12672925	43.04794055	57.27814981	156.2111321	136.9404622	101.2721857
ENSG00000122786	13470	10445	13938	25388	25727	18953	63.23447704	44.41997012	49.23869905	113.8726565	113.7037122	88.9537413
ENSG00000187398	220	245	457	530	641	477	1.148534805	1.158700106	1.795384428	2.643637943	3.150494104	2.489658497
ENSG00000004399	2586	2145	2166	4053	5735	3126	12.27079133	9.22050758	7.734322496	18.37491256	25.61985305	14.82971978
ENSG00000138641	[illegible]	[illegible]	[illegible]	[illegible]	[illegible]	[illegible]	[illegible]	[illegible]	[illegible]	[illegible]	[illegible]	[illegible]
ENSG00000130518	[illegible]	[illegible]	[illegible]	[illegible]	[illegible]	[illegible]	[illegible]	[illegible]	[illegible]	[illegible]	[illegible]	[illegible]
ENSG00000163235	[illegible]	[illegible]	[illegible]	[illegible]	[illegible]	[illegible]	[illegible]	[illegible]	[illegible]	[illegible]	[illegible]	[illegible]

Supplemental Table 8 - List of up regulated genes by RNAseq

gene_id	CH_0323	CH_0407	CH_0603	DMSO_0323	DMSO_0407	DMSO_0603	CHIR	DMSO	log2FoldChange	pvalue	padj	gene_name	gene_chr	gene_start	gene_end	gene_strand	gene_length	gene_biotype	gene_description	tf_family	CH_0323_count	CH_0407_count	CH_0603_count	DMSO_0323_count	DMSO_0407_count	DMSO_0603_count	CH_0323_fpkm	CH_0407_fpkm	CH_0603_fpkm	DMSO_0323_fpkm	DMSO_0407_fpkm	DMSO_0603_fpkm
ENSG00000203837	34.3467814	6.80640192	67.039989	3.076535635	0	2.146179214	36.0643908	1.74090495	4.382717783	4.69E-05	0.00285117	PNLIPRP3	10	116427867	116477957	+	2354	protein_coding	pancreatic lipase related protein 3 [Source:HGNC Symbol;Acc:HGNC:23492]	-	32	7	83	3	0	2	0.628141288	0.124476935	1.226041663	0.056264342	0	0.039249785
ENSG00000259070	30.0534338	21.3915489	27.4621642	2.051023757	3.031492406	0	26.3023823	1.69417205	3.940635771	0.00013616	0.00657973	LINC00639	14	38749339	38948273	-	4860	lincRNA	long intergenic non-protein coding RNA 639 [Source:HGNC Symbol;Acc:HGNC:27502]	-	28	22	34	2	3	0	0.266216876	0.189488874	0.243263103	0.018168211	0.026853319	0
ENSG00000154545	22.5400753	294.619969	70.2708319	8.204095028	18.18895443	10.73089607	129.143625	12.3746485	3.382914894	0.00114513	0.03244458	MAGED4	X	52184823	52192268	+	4809	protein_coding	MAGE family member D4 [Source:HGNC Symbol;Acc:HGNC:23793]	-	21	303	87	8	18	10	0.201780102	2.637455582	0.62906869	0.073443549	0.162828608	0.096063624
ENSG00000172348	24.6867492	16.5298332	23.4236106	1.025511878	4.041989874	2.146179214	21.546731	2.40456032	3.160243114	0.00156051	0.04076723	RCAN2	6	46220736	46491972	-	3712	protein_coding	regulator of calcineurin 2 [Source:HGNC Symbol;Acc:HGNC:3041]	-	23	17	29	1	4	2	0.286308136	0.191707126	0.271658704	0.011893522	0.046877561	0.024890623
ENSG00000187950	24.6867492	40.8384115	16.1542142	4.102047514	0	5.365448036	27.2264583	3.15583185	3.115987071	0.00126038	0.03489426	OVCH1	12	29412474	29497686	-	3958	protein_coding	ovochymase 1 [Source:HGNC Symbol;Acc:HGNC:23080]	-	23	42	20	4	0	5	0.26851334	0.444192072	0.175706489	0.044617235	0	0.058359015
ENSG00000119686	59.0335306	59.312931	67.039989	0	10.10497469	13.95016489	61.7954835	8.01837986	2.951150115	2.50E-05	0.00181347	FLVCR2	14	75578617	75663214	+	4913	protein_coding	feline leukemia virus subgroup C cellular receptor family member 2 [Source:HGNC Symbol;Acc:HGNC:20105]	-	55	61	83	0	10	13	0.517284834	0.519733097	0.587441904	0	0.088545444	0.122239152
ENSG00000042781	16.1000538	35.0043527	58.1551712	0	6.062984811	8.584716857	36.4198592	4.88256722	2.908127233	0.00103856	0.0301593	USH2A	1	215622894	216423396	-	20506	protein_coding	usherin [Source:HGNC Symbol;Acc:HGNC:12601]	-	15	36	72	0	6	8	0.033800578	0.07348841	0.122091418	0	0.012728677	0.018022821
ENSG00000223861	15.0267169	49.5894997	20.1927678	3.076535635	4.041989874	4.292358429	28.2696614	3.80362798	2.894722021	0.00104095	0.0301593	AL365436.1	1	151557446	151557940	+	495	processed_pseudogene	ribosomal protein S10 (RPS10) pseudogene	-	14	51	25	3	4	4	1.306882846	4.312829407	1.756177483	0.267568204	0.351534355	0.373309063
ENSG00000196872	86.9402905	117.653519	144.580217	9.229606906	32.33591899	10.73089607	116.391342	17.4321407	2.736786501	6.14E-08	1.04E-05	KIAA1211L	2	98793846	98936259	-	4705	protein_coding	KIAA1211 like [Source:HGNC Symbol;Acc:HGNC:33454]	-	81	121	179	9	32	10	0.795498219	1.076522337	1.322899944	0.084450326	0.295871636	0.098187028
ENSG00000133083	165.293886	33.0596664	189.004306	19.48472569	12.12596962	26.82724018	129.119286	19.4793118	2.731855813	1.38E-06	0.00016445	DCLK1	13	35768652	36131306	-	14375	protein_coding	doublecortin like kinase 1 [Source:HGNC Symbol;Acc:HGNC:2700]	-	154	34	234	19	12	25	0.495024493	0.099007562	0.566032801	0.058353135	0.036315027	0.080342603
ENSG00000183036	1811.79272	2897.58253	2070.16255	279.9647428	323.3591899	430.3089325	2259.84593	344.544288	2.713988665	1.20E-34	1.89E-30	PCP4	21	39867317	39929397	+	1106	protein_coding	Purkinje cell protein 4 [Source:HGNC Symbol;Acc:HGNC:8742]	-	1688	2980	2563	273	320	401	70.52305802	112.7868429	80.57996491	10.89747717	12.5865827	16.74954396
ENSG00000251003	125.58042	122.515234	142.157085	15.38267818	22.23094431	22.53488175	130.084246	20.0495014	2.699260167	2.26E-11	8.09E-09	ZFPM2-AS1	8	105546089	106060524	-	2667	processed_transcript	ZFPM2 antisense RNA 1 [Source:HGNC Symbol;Acc:HGNC:50698]	-	117	126	176	15	22	21	2.027106969	1.977629047	2.294685899	0.248305701	0.35884975	0.363755878
ENSG00000270605	18.2467276	29.1702939	31.5007177	3.076535635	6.062984811	3.219268821	26.3059131	4.11959642	2.674367472	0.00166726	0.04256784	AL353622.1	1	28239509	28241453	-	1945	antisense	novel transcript	-	17	30	39	3	6	3	0.403871434	0.645652671	0.697234063	0.068095764	0.134197562	0.071255008
ENSG00000129151	24.6867492	65.1469898	26.6544535	3.076535635	11.11547215	5.365448036	38.8293975	6.51915194	2.570987394	0.00079579	0.02467741	BBOX1	11	27040725	27127809	+	2464	protein_coding	gamma-butyrobetaine hydroxylase 1 [Source:HGNC Symbol;Acc:HGNC:964]	-	23	67	33	3	11	5	0.431321347	1.138233601	0.465700636	0.053752541	0.194207038	0.093743905
ENSG00000261235	21.4667384	53.4788722	24.2313213	6.153071271	2.020994937	8.584716857	33.0589773	5.58626102	2.570244509	0.00149089	0.03933995	AC092142.1	16	82044336	82139631	-	1030	antisense	uncharacterized LOC105371363 [Source:NCBI gene;Acc:105371363]	-	20	55	30	6	2	8	0.897235795	2.235232831	1.012785849	0.257177206	0.084470634	0.358811624
ENSG00000270127	19.3200646	58.3405878	22.6158999	5.127559392	7.07348228	5.365448036	33.4255174	5.85549657	2.511512252	0.00141079	0.03779626	AC027020.2	15	100547765	100550153	-	2389	lincRNA	novel transcript	-	18	60	28	5	7	5	0.348153027	1.051313893	0.407544913	0.09240007	0.127466151	0.096686891
ENSG00000107562	48.3001614	98.2066562	102.57926	15.38267818	9.094477217	25.75415057	83.0286926	16.7437687	2.316564444	1.56E-05	0.00122651	CXCL12	10	44370165	44386493	-	6077	protein_coding	C-X-C motif chemokine ligand 12 [Source:HGNC Symbol;Acc:HGNC:10672]	-	45	101	127	15	9	24	0.342166193	0.695711913	0.726688152	0.108973392	0.064426755	0.182446588
ENSG00000077498	845.789493	1160.9777	1184.1039	255.3524577	223.3199405	182.4252332	1063.6237	220.365877	2.271061013	7.85E-26	2.47E-22	TYR	11	89177452	89295759	+	3016	protein_coding	tyrosinase [Source:HGNC Symbol;Acc:HGNC:12442]	-	788	1194	1466	249	221	170	12.07281931	16.57182325	16.90192724	3.644907049	3.187674137	2.603942113
ENSG00000165092	42.9334768	132.238666	105.810103	17.43370193	13.13646709	27.90032979	93.6607486	19.4901663	2.269669866	1.75E-05	0.00134478	ALDH1A1	9	72900662	73080442	-	2849	protein_coding	aldehyde dehydrogenase 1 family member A1 [Source:HGNC Symbol;Acc:HGNC:402]	-	40	136	131	17	13	26	0.648755963	1.998222119	1.598867376	0.263435876	0.198501542	0.421594212
ENSG00000140961	129.873767	141.962097	119.541185	6.153071271	35.3674114	41.85049468	130.459017	27.7903258	2.232403235	8.00E-06	0.00072817	OSGIN1	16	83948282	83966332	+	3460	protein_coding	oxidative stress induced growth inhibitor 1 [Source:HGNC Symbol;Acc:HGNC:30093]	-	121	146	148	6	35	39	1.61593204	1.766339007	1.487370663	0.076558532	0.440052941	0.520717591
ENSG00000166473	1142.03048	1455.59767	1228.52799	258.4289934	324.3696874	263.9800434	1275.38538	282.259575	2.175584295	1.76E-29	1.39E-25	PKD1L2	16	81100875	81220370	-	18637	polymorphic_pseudogene	polycystin 1 like 2 (gene/pseudogene) [Source:HGNC Symbol;Acc:HGNC:21715]	-	1064	1497	1521	252	321	246	2.638028258	3.362351387	2.837832797	0.596956909	0.749276324	0.609779533
ENSG00000278035	41.8601399	66.1193329	31.5007177	4.102047514	16.1679595	10.73089607	46.4933968	10.3336344	2.167197855	0.0011618	0.032723	AL121895.2	20	36233851	36234297	+	447	lincRNA	novel transcript	-	39	68	39	4	16	10	4.031539363	6.367936037	3.033826068	0.395067147	1.557132043	1.033489856
ENSG00000107165	2462.23489	1786.19433	2062.08545	767.0828851	428.4509267	314.4152549	2103.50489	503.316356	2.062867039	4.13E-15	2.25E-12	TYRP1	9	12685439	12710290	+	3741	protein_coding	tyrosinase related protein 1 [Source:HGNC Symbol;Acc:HGNC:12450]	-	2294	1837	2553	748	424	293	28.33475919	20.5550601	23.72994334	8.827390465	4.930501903	3.618209032
ENSG00000283031	46.1534875	35.0043527	52.5011962	8.204095028	7.07348228	17.16943371	44.5530122	10.8156703	2.048021661	0.00123416	0.03434928	AC009242.1	2	23667208	23685453	-	2706	lincRNA	novel transcript	-	43	36	65	8	7	16	0.734267801	0.556893324	0.835255144	0.130521075	0.112533864	0.273152973
ENSG00000132386	383.18128	317.956204	683.323262	111.7807948	63.66134052	160.9634411	461.486915	112.135192	2.043586767	2.95E-09	6.93E-07	SERPINF1	17	1761959	1777574	+	3069	protein_coding	serpin family F member 1 [Source:HGNC Symbol;Acc:HGNC:8824]	-	357	327	846	109	63	150	5.375082671	4.460136673	9.585330003	1.568007217	0.89301066	2.25791772
ENSG00000164175	398.207997	412.273487	457.971973	112.8063066	101.0497469	103.0166023	422.817819	105.624219	2.001665261	1.26E-17	1.17E-14	SLC45A2	5	33944616	33984730	-	3267	protein_coding	solute carrier family 45 member 2 [Source:HGNC Symbol;Acc:HGNC:16472]	-	371	424	567	110	100	96	5.247332637	5.432678754	6.034864441	1.486490022	1.331569527	1.357487502
ENSG00000041353	787.829299	387.964909	1192.18101	155.8778055	151.5746203	298.3189108	789.325073	201.923779	1.967341127	0.0003037	0.01215436	RAB27B	18	54717860	54895516	+	7432	protein_coding	RAB27B, member RAS oncogene family [Source:HGNC Symbol;Acc:HGNC:9767]	-	734	399	1476	152	150	278	4.563564357	2.247317831	6.90580405	0.902934682	0.878008136	1.7280362
ENSG00000109819	266.187556	281.979508	453.125709	61.53071271	83.87128989	113.7474984	333.764258	86.383167	1.952853394	5.43E-10	1.56E-07	PPARGC1A	4	23755041	23904089	-	10870	protein_coding	PPARG coactivator 1 alpha [Source:HGNC Symbol;Acc:HGNC:9237]	-	248	290	561	60	83	106	1.054231424	1.116775189	1.794596895	0.243691372	0.33217086	0.450495091
ENSG00000125965	191.053972	165.298332	182.542621	23.5867732	78.81880255	42.92358429	179.631642	48.4430533	1.889381645	2.11E-06	0.00023634	GDF5	20	35433347	35454746	-	2646	protein_coding	growth differentiation factor 5 [Source:HGNC Symbol;Acc:HGNC:4220]	-	178	170	226	23	78	40	3.108450694	2.689406093	2.9699709	0.383757118	1.28238298	0.698367295
ENSG00000148053	103.040344	103.068372	163.157564	17.43370193	44.46188862	40.77740507	123.08876	34.2243319	1.849223341	2.16E-05	0.00158636	NTRK2	9	84668551	85027070	+	20363	protein_coding	neurotrophic receptor tyrosine kinase 2 [Source:HGNC Symbol;Acc:HGNC:8032]	-	96	106	202	17	44	38	0.217842841	0.217902096	0.344939717	0.036857477	0.093999144	0.086209589
ENSG00000197915	81.5736059	94.3172837	299.660674	34.86740387	33.34641646	65.45846604	158.517188	44.5574288	1.836037585	0.00018297	0.00823513	HRNR	1	152212082	152224193	-	9623	protein_coding	hornerin [Source:HGNC Symbol;Acc:HGNC:20846]	-	76	97	371	34	33	61	0.364936185	0.42194763	1.34059322	0.155986452	0.149182004	0.292841815
ENSG00000139289	1193.55065	1363.22507	1890.85078	470.7099522	326.3906823	474.3056064	1482.54217	423.80208	1.807500206	4.47E-16	3.20E-13	PHLDA1	12	76025447	76033932	-	8069	protein_coding	pleckstrin homology like domain family A member 1 [Source:HGNC Symbol;Acc:HGNC:8933]	-	1112	1402	2341	459	323	442	6.367938968	7.273201189	10.08823738	2.511374139	1.74138897	2.530557997
ENSG00000118322	827.542765	592.156967	682.515551	226.6381251	211.1939709	183.4983228	700.738428	207.11014	1.757627217	5.53E-16	3.79E-13	ATP10B	5	160563120	160852214	-	11310	protein_coding	ATPase phospholipid transporting 10B (putative) [Source:HGNC Symbol;Acc:HGNC:13543]	-	771	609	845	221	209	171	3.149964024	2.253990029	2.597931518	0.862676795	0.803890069	0.698469179
ENSG00000158270	203.934015	175.021764	165.580696	76.91339088	46.48288355	38.63122586	181.512158	54.0091668	1.746830662	5.30E-07	7.51E-05	COLEC12	18	316740	500722	-	7340	protein_coding	collectin subfamily member 12 [Source:HGNC Symbol;Acc:HGNC:16016]	-	190	180	205	75	46	36	1.196110662	1.026534969	0.971161363	0.451111243	0.272630697	0.226579275
ENSG00000135253	1702.31235	2653.5244	2284.20589	477.8885354	853.8703609	696.435155	2213.34755	676.064684	1.711117603	5.74E-14	2.74E-11	KCP	7	128862451	128910719	-	8259	protein_coding	kielin/chordin-like protein [Source:HGNC Symbol;Acc:HGNC:17585]	-	1586	2729	2828	466	845	649	8.873389338	13.8316303	11.90653886	2.491018186	4.450842488	3.630203506
ENSG00000198203	701.962346	921.781288	951.483218	273.8116715	252.6243671	261.8338641	858.40895	262.756634	1.708685395	2.92E-17	2.30E-14	SULT1C2	2	108288639	108309915	+	8399	protein_coding	sulfotransferase family 1C member 2 [Source:HGNC Symbol;Acc:HGNC:11456]	-	654	948	1178	267	250	244	3.598023435	4.724741572	4.876983698	1.403466749	1.294867736	1.342072527
ENSG00000182612	6755.58257	9798.30173	7038.39114	2289.968025	2872.844303	2653.750598	7864.09181	2605.52098	1.593694902	1.35E-18	1.52E-15	TSPAN10	17	81637171	81648749	+	4745	protein_coding	tetraspanin 10 [Source:HGNC Symbol;Acc:HGNC:29942]	-	6294	10077	8714	2233	2843	2473	61.29207755	88.89807251	63.85794428	20.77643138	26.06475369	24.07695942
ENSG00000257681	82.6469428	80.7044798	63.8091462	13.33165442	38.3989038	23.60797136	75.7201896	25.1128432	1.589790061	0.00134214	0.03645298	AC025265.1	12	103746315	103768858	-	1063	antisense	novel transcript, antisense to STAB2	-	77	83	79	13	38	22	3.347119988	3.268452146	2.584207732	0.539918906	1.555117879	0.956099646
ENSG00000276772	96.6003228	77.7874505	71.8862533	18.45921381	40.41989874	24.68106096	82.0913422	27.8533912	1.556525477	0.000766	0.02418185	AC025271.4	15	57553955	57555680	+	1726	lincRNA	novel transcript	-	90	80	89	18	40	23	2.409436797	1.940199992	1.793010406	0.460415739	1.008166314	0.615603083
ENSG00000272899	582.821947	830.381034	823.057215	272.7861597	244.5403874	250.0298785	745.420065	255.785475	1.543894571	5.44E-13	2.32E-10	ATP6V1FNB	7	128866308	128872044	+	3902	protein_coding	ATP6V1F neighbor [Source:HGNC Symbol;Acc:HGNC:52392]	-	543	854	1019	266	242	233	6.430228191	9.1615279	9.08072479	3.009628004	2.697994645	2.758559763
ENSG00000170989	319.854402	263.504988	462.818238	133.3165442	74.77681267	167.4019787	348.725876	125.165112	1.480566735	3.96E-06	0.00040656	S1PR1	1	101236865	101243713	+	5223	protein_coding	sphingosine-1-phosphate receptor 1 [Source:HGNC Symbol;Acc:HGNC:3165]	-	298	271	573	130	74	156	2.636392447	2.17193372	3.814768526	1.098858506	0.616346134	1.379806904
ENSG00000166963	205.007352	75.8427642	105.002392	39.99496326	44.46188862	53.65448036	128.617503	46.0371107	1.48012574	0.00053066	0.0183165	MAP1A	15	43510958	43531620	+	10629	protein_coding	microtubule associated protein 1A [Source:HGNC Symbol;Acc:HGNC:6835]	-	191	78	130	39	44	50	0.830337746	0.307184642	0.425289382	0.161990911	0.180083222	0.217315818
ENSG00000143772	1230.04411	784.680906	1268.91353	388.6690019	361.7580937	434.6012909	1094.54618	395.009462	1.470764805	1.51E-11	5.54E-09	ITPKB	1	226631690	226739323	-	7036	protein_coding	inositol-trisphosphate 3-kinase B [Source:HGNC Symbol;Acc:HGNC:6179]	-	1146	807	1571	379	358	405	7.526145454	4.801147039	7.763971794	2.378109385	2.213452355	2.659150597
ENSG00000164627	247.940828	255.726243	251.198031	113.8318185	82.86079242	90.139527	251.621701	95.6107126	1.395995701	2.73E-07	4.14E-05	KIF6	6	39329990	39725405	-	11868	protein_coding	kinesin family member 6 [Source:HGNC Symbol;Acc:HGNC:21202]	-	231	263	311	111	82	84	0.899390431	0.927631556	0.911205738	0.41291807	0.300572537	0.326975709
ENSG00000180332	81.5736059	107.930088	90.4635997	41.02047514	39.40940127	27.90032979	93.322431	36.1100687	1.368915697	0.00077666	0.02437207	KCTD4	13	45192853	45194717	-	1865	protein_coding	potassium channel tetramerization domain containing 4 [Source:HGNC Symbol;Acc:HGNC:23227]	-	76	111	112	40	39	26	1.882992442	2.491388443	2.088203317	0.946890159	0.90970117	0.644033196
ENSG00000285437	97.6736597	106.957744	76.7325176	27.68882072	27.28343165	54.72756996	93.7879739	36.5666074	1.359961176	0.00183551	0.04567909	POLR2J3	7	102562133	102572583	-	7195	protein_coding	RNA polymerase II subunit J3 [Source:HGNC Symbol;Acc:HGNC:33853]	-	91	110	95	27	27	51	0.584419118	0.639969372	0.459120201	0.165672877	0.163247278	0.327456126
ENSG00000170289	172.807244	96.2619699	125.19516	69.73480773	43.45139115	41.85049468	131.421458	51.6788979	1.344596851	0.00051303	0.01787994	CNGB3	8	86553977	86743675	-	5025	protein_coding	cyclic nucleotide gated channel beta 3 [Source:HGNC Symbol;Acc:HGNC:2153]	-	161	99	155	68	43	39	1.480483701	0.82470083	1.072578845	0.59743587	0.372259142	0.358543854
ENSG00000138316	481.928277	601.880398	538.743044	184.5921381	190.9840216	276.8571186	540.850573	217.477759	1.31540053	1.93E-08	3.70E-06	ADAMTS14	10	70672803	70762441	+	5269	protein_coding	ADAM metallopeptidase with thrombospondin type 1 motif 14 [Source:HGNC Symbol;Acc:HGNC:14899]	-	449	619	667	180	189	258	3.937603324	4.917674204	4.401809365	1.508213258	1.560438252	2.262065878
ENSG00000163762	257.600861	256.698587	440.202338	181.5156025	127.322681	79.40863093	318.167262	129.415638	1.298578395	8.70E-05	0.00464823	TM4SF18	3	149318498	149334414	-	4482	protein_coding	transmembrane 4 L six family member 18 [Source:HGNC Symbol;Acc:HGNC:25181]	-	240	264	545	177	126	74	2.474304871	2.465638357	4.22822651	1.74349161	1.222958374	0.762734883
ENSG00000108924	122.360409	246.002812	243.120924	48.19905829	95.99725951	105.1627815	203.828048	83.1196998	1.296409044	0.00052311	0.01815092	HLF	17	55265012	55325065	+	6602	protein_coding	HLF, PAR bZIP transcription factor [Source:HGNC Symbol;Acc:HGNC:4977]	TF_bZIP	114	253	301	47	95	98	0.797890239	1.604140132	1.585347859	0.314297398	0.625980879	0.685747601
ENSG00000138642	148.120495	81.676823	142.157085	42.04598702	42.44089368	67.60464525	123.984801	50.6971753	1.291822173	0.00108243	0.03105918	HERC6	4	88378739	88443111	+	5793	protein_coding	HECT and RLD domain containing E3 ubiquitin protein ligase family member 6 [Source:HGNC Symbol;Acc:HGNC:26072]	-	138	84	176	41	42	63	1.100751735	0.606978154	1.056434886	0.312463128	0.315397861	0.502401309
ENSG00000235718	1907.31971	1865.92647	2276.9365	648.1235072	696.2327558	1136.401894	2016.72756	826.919386	1.2867246	3.55E-09	8.09E-07	MFRP	11	119338942	119346673	-	4064	protein_coding	membrane frizzled-related protein [Source:HGNC Symbol;Acc:HGNC:18121]	-	1777	1919	2819	632	689	1059	20.20447403	19.76599032	24.11987048	6.865652634	7.375279867	12.0380461
ENSG00000248174	173.880581	175.994107	189.812017	66.6582721	65.68233545	93.35879582	179.895568	75.2331345	1.259420555	4.20E-05	0.00262486	LINC02268	4	174094660	174220398	-	1659	lincRNA	long intergenic non-protein coding RNA 2268 [Source:HGNC Symbol;Acc:HGNC:53183]	-	162	181	235	65	65	87	4.512138783	4.566984017	4.925553844	1.72975828	1.704433074	2.422627305
ENSG00000119922	180.320603	92.3725974	136.50311	65.63276022	58.60885318	49.36212193	136.39877	57.8679118	1.235211934	0.00084628	0.02593672	IFIT2	10	89283694	89309276	+	4101	protein_coding	interferon induced protein with tetratricopeptide repeats 2 [Source:HGNC Symbol;Acc:HGNC:5409]	-	168	95	169	64	58	46	1.892924678	0.96968603	1.432948328	0.688983232	0.615249411	0.51818138
ENSG00000210194	153.48718	399.633027	177.696356	89.21953343	101.0497469	121.2591256	243.605521	103.842802	1.230375667	0.00070239	0.02267361	MT-TE	MT	14674	14742	-	69	Mt_tRNA	mitochondrially encoded tRNA glutamic acid [Source:HGNC Symbol;Acc:HGNC:7479]	-	143	411	220	87	100	113	95.7636669	249.338897	110.868248	55.66581982	63.04692241	75.65595087
ENSG00000189007	178.173929	292.675282	276.237063	115.8828423	94.98676204	121.2591256	249.028758	110.709577	1.171504615	6.25E-05	0.00355509	ADAT2	6	143422832	143450673	-	7175	protein_coding	adenosine deaminase, tRNA specific 2 [Source:HGNC Symbol;Acc:HGNC:21172]	-	166	301	342	113	94	113	1.069054887	1.756070281	1.657439922	0.695304413	0.569926604	0.727562454
ENSG00000135069	275.847588	449.222526	479.780163	196.8982807	181.8895443	156.6710826	401.616759	178.486303	1.171247177	9.98E-06	0.00085008	PSAT1	9	78297143	78330093	+	2188	protein_coding	phosphoserine aminotransferase 1 [Source:HGNC Symbol;Acc:HGNC:19129]	-	257	462	594	192	180	146	5.427497427	8.838772602	9.440015819	3.874113666	3.578806107	3.082614943
ENSG00000132481	373.521248	478.39282	534.704491	176.3880431	162.6900924	277.9302083	462.206186	205.669448	1.170084577	8.61E-06	0.00075805	TRIM47	17	75874161	75878575	-	3142	protein_coding	tripartite motif containing 47 [Source:HGNC Symbol;Acc:HGNC:19020]	-	348	492	662	172	161	259	5.117842115	6.5547514	7.326311895	2.416800009	2.229116044	3.808091059
ENSG00000155761	343.467814	482.282193	446.664023	234.8422202	189.9735241	147.0132762	424.13801	190.609673	1.154049239	5.43E-06	0.00052847	SPAG17	1	117953861	118185223	-	9552	protein_coding	sperm associated antigen 17 [Source:HGNC Symbol;Acc:HGNC:26620]	-	320	496	553	229	188	137	1.547994756	2.173625226	2.013095659	1.058423847	0.856202552	0.662582551
ENSG00000253417	2250.78752	2000.10982	2468.36393	1068.583377	892.2692647	1068.797249	2239.75376	1009.8833	1.149431286	7.58E-12	3.06E-09	LINC02159	5	160931778	160938626	-	2323	lincRNA	long intergenic non-protein coding RNA 2159 [Source:HGNC Symbol;Acc:HGNC:27749]	-	2097	2057	3056	1042	883	996	41.71219473	37.06656871	45.74437885	19.80327219	16.53577203	19.80723571
ENSG00000087303	1070.11691	436.582066	864.250461	495.3222373	360.7475963	212.4717422	790.316479	356.180525	1.149294698	0.0002972	0.01203176	NID2	14	52004803	52069228	-	6392	protein_coding	nidogen 2 [Source:HGNC Symbol;Acc:HGNC:13389]	-	997	449	1070	483	357	198	7.207293576	2.940403138	5.820772239	3.336021279	2.429654004	1.431008341
ENSG00000203727	457.241528	540.622781	382.047166	173.3115075	174.8160621	274.7109394	459.970492	207.612836	1.147860492	7.68E-06	0.00071177	SAMD5	6	147508927	147737547	+	6320	protein_coding	sterile alpha motif domain containing 5 [Source:HGNC Symbol;Acc:HGNC:21180]	-	426	556	473	169	173	256	3.114629132	3.682603963	2.602421614	1.180560026	1.190808723	1.871270746
ENSG00000210174	157.780527	194.468626	124.38745	56.40315331	76.79780761	82.62789975	158.878868	71.9429536	1.142077005	0.00066135	0.02156993	MT-TR	MT	10405	10469	+	65	Mt_tRNA	mitochondrially encoded tRNA arginine [Source:HGNC Symbol;Acc:HGNC:7496]	-	147	200	154	55	76	77	104.5003629	128.7994303	82.38363663	37.3566377	50.86431709	54.72567283
ENSG00000143387	1970.64658	1620.896	1788.27151	943.4709282	784.1460356	726.481664	1793.27137	818.032876	1.132113549	1.20E-10	3.78E-08	CTSK	1	150796208	150808323	-	1887	protein_coding	cathepsin K [Source:HGNC Symbol;Acc:HGNC:2536]	-	1836	1667	2214	920	776	677	44.95878824	36.97949726	40.7980411	21.52456458	17.88968952	16.57412118
ENSG00000280744	177.100592	159.464273	220.505024	87.16850967	57.59835571	118.0398568	185.689963	87.6022407	1.086462512	0.00117806	0.03308018	LINC01173	2	234682668	234717764	+	331	lincRNA	long intergenic non-protein coding RNA 1173 [Source:HGNC Symbol;Acc:HGNC:49545]	-	165	164	273	85	57	110	23.03402167	20.74021037	28.67928026	11.33729323	7.491345795	15.35247619
ENSG00000183828	459.388202	467.697046	264.121403	170.2349718	240.4983975	151.3056346	397.068883	187.346335	1.081595047	0.00014838	0.00704037	NUDT14	14	105172938	105181323	-	1916	protein_coding	nudix hydrolase 14 [Source:HGNC Symbol;Acc:HGNC:20141]	-	428	481	327	166	238	141	10.32195794	10.50864871	5.934523346	3.824996402	5.403739873	3.399674589
ENSG00000175592	335.954456	534.788722	659.09194	156.9033174	259.6978494	310.1228965	509.945039	242.241354	1.075546006	0.0002611	0.01082395	FOSL1	11	65892049	65900573	-	1887	protein_coding	FOS like 1, AP-1 transcription factor subunit [Source:HGNC Symbol;Acc:HGNC:13718]	TF_bZIP	313	550	816	153	257	289	7.664542875	12.20079334	15.03667639	3.579628674	5.924806969	7.075215687
ENSG00000005249	217.887395	208.08143	315.007177	105.6277235	114.1862139	133.0631113	246.992001	117.625683	1.072708688	0.00016348	0.00757456	PRKAR2B	7	107044649	107161811	+	4318	protein_coding	protein kinase cAMP-dependent type II regulatory subunit beta [Source:HGNC Symbol;Acc:HGNC:9392]	-	203	214	390	103	113	124	2.172337106	2.074571648	3.140621242	1.053108297	1.138436438	1.326639086
ENSG00000157388	309.121033	317.956204	419.201859	170.2349718	107.1127317	221.0564591	348.759699	166.134721	1.071919118	0.00020916	0.00917812	CACNA1D	3	53328963	53813733	+	20588	protein_coding	calcium voltage-gated channel subunit alpha1 D [Source:HGNC Symbol;Acc:HGNC:1391]	-	288	327	519	166	106	206	0.646386308	0.664861057	0.876570383	0.355969162	0.223977652	0.462239231
ENSG00000002587	302.681011	303.371057	268.159956	125.1124492	173.8055646	116.9667672	291.404008	138.62826	1.070447162	4.40E-05	0.00271303	HS3ST1	4	11393150	11429765	-	8166	protein_coding	heparan sulfate-glucosamine 3-sulfotransferase 1 [Source:HGNC Symbol;Acc:HGNC:5194]	-	282	312	332	122	172	109	1.595708481	1.599346342	1.413716422	0.659582163	0.916288115	0.616638822
ENSG00000178445	178.173929	199.330342	231.812974	72.81134337	92.96576711	126.6245736	203.105748	97.467228	1.061565082	0.00056605	0.01909416	GLDC	9	6532464	6645783	-	8939	protein_coding	glycine decarboxylase [Source:HGNC Symbol;Acc:HGNC:4313]	-	166	205	287	71	92	118	0.858090258	0.959980092	1.116417291	0.350661317	0.447725544	0.609827228
ENSG00000132541	448.654832	723.423289	537.935334	257.4034815	253.6348646	313.3421653	570.004485	274.793504	1.05322331	7.28E-06	0.00068262	RIDA	8	98102344	98117241	-	1567	protein_coding	reactive intermediate imine deaminase A homolog [Source:HGNC Symbol;Acc:HGNC:16897]	-	418	744	666	251	251	292	12.32596999	19.87473021	14.77878828	7.071689321	6.968153473	8.608502228
ENSG00000184261	138.460463	125.432264	156.695878	62.55622459	60.62984811	80.48172054	140.196201	67.8892644	1.048055488	0.00144631	0.03855119	KCNK12	2	47516581	47570939	-	6685	protein_coding	potassium two pore domain channel subfamily K member 12 [Source:HGNC Symbol;Acc:HGNC:6274]	-	129	129	194	61	60	75	0.89166582	0.80776606	1.009099319	0.402853249	0.390447657	0.518290911
ENSG00000279759	229.694101	325.734949	237.466949	97.42362845	129.343676	162.0365307	264.298666	129.601278	1.028679375	0.00037156	0.01400299	AC118344.2	19	40355842	40360606	+	4765	TEC	TEC	-	214	335	294	95	128	151	2.075222603	2.942925072	2.145448135	0.880195508	1.168584299	1.46395519
ENSG00000209082	271.554241	718.561574	378.816324	273.8116715	199.0680013	203.8870254	456.310713	225.588899	1.016426974	0.00128568	0.03542995	MT-TL1	MT	3230	3304	+	75	Mt_tRNA	mitochondrially encoded tRNA leucine 1 (UUA/G) [Source:HGNC Symbol;Acc:HGNC:7490]	-	253	739	469	267	197	190	155.873784	412.4587088	217.4428712	157.169563	114.2662422	117.0323913
ENSG00000166501	212.52071	192.52394	224.543578	134.3420561	91.95526964	85.84716857	209.862743	104.048165	1.012128098	0.00057178	0.01924613	PRKCB	16	23835946	24220611	+	11086	protein_coding	protein kinase C beta [Source:HGNC Symbol;Acc:HGNC:9395]	-	198	198	278	131	91	80	0.825285351	0.747631548	0.871973961	0.521692831	0.35709149	0.333371795
ENSG00000210140	1570.29191	3304.9943	1899.73559	759.9043019	1232.806912	1369.262339	2258.3406	1120.65785	1.01101256	0.00013402	0.00656877	MT-TC	MT	5761	5826	-	66	Mt_tRNA	mitochondrially encoded tRNA cysteine [Source:HGNC Symbol;Acc:HGNC:7477]	-	1463	3399	2352	741	1220	1276	1024.26943	2155.780464	1239.158832	495.6700978	804.1348376	893.1419333
ENSG00000223812	186.760624	265.449675	267.352245	104.6022116	102.0602443	152.3787242	239.854181	119.680393	1.005307915	0.00059742	0.01981293	AC073365.1	3	191425526	191591097	+	2535	lincRNA	novel transcript	-	174	273	331	102	101	142	3.171648901	4.507980059	4.54029033	1.776399555	1.733230778	2.587760753
ENSG00000071575	778.169267	775.929818	826.288058	424.5619177	383.989038	377.7275417	793.462381	395.426166	1.004901399	2.84E-08	5.14E-06	TRIB2	2	12716889	12742734	+	4588	protein_coding	tribbles pseudokinase 2 [Source:HGNC Symbol;Acc:HGNC:30809]	-	725	798	1023	414	380	352	7.301774523	7.280761167	7.753286265	3.983780299	3.6030739	3.544320572
ENSG00000138061	6313.36776	4449.44217	4922.99679	2765.805536	2253.409355	2801.836964	5228.60224	2607.01729	1.00394551	1.47E-08	2.92E-06	CYP1B1	2	38066973	38109902	-	6014	protein_coding	cytochrome P450 family 1 subfamily B member 1 [Source:HGNC Symbol;Acc:HGNC:2597]	-	5882	4576	6095	2697	2230	2611	45.19344178	31.85076699	35.24064762	19.79866787	16.13074485	20.05659428
ENSG00000033122	450.801506	528.954663	554.089548	206.1278876	242.5193924	318.7076133	511.281906	255.784964	1.000424229	1.52E-05	0.00120165	LRRC7	1	69568398	70151945	+	14652	protein_coding	leucine rich repeat containing 7 [Source:HGNC Symbol;Acc:HGNC:18531]	-	420	544	686	201	240	297	1.324543425	1.554172759	1.628023991	0.605644245	0.712569639	0.936425606
