# Supplementary material for: GSK3 inhibition reduces ECM production and prevents age-related macular degeneration–like pathology
Source: JCI Insight. 2024 Aug 8;9(15):e178050. doi: 10.1172/jci.insight.178050 (PMC11383595; doi:10.1172/jci.insight.178050)

Supplemental Figure 12.

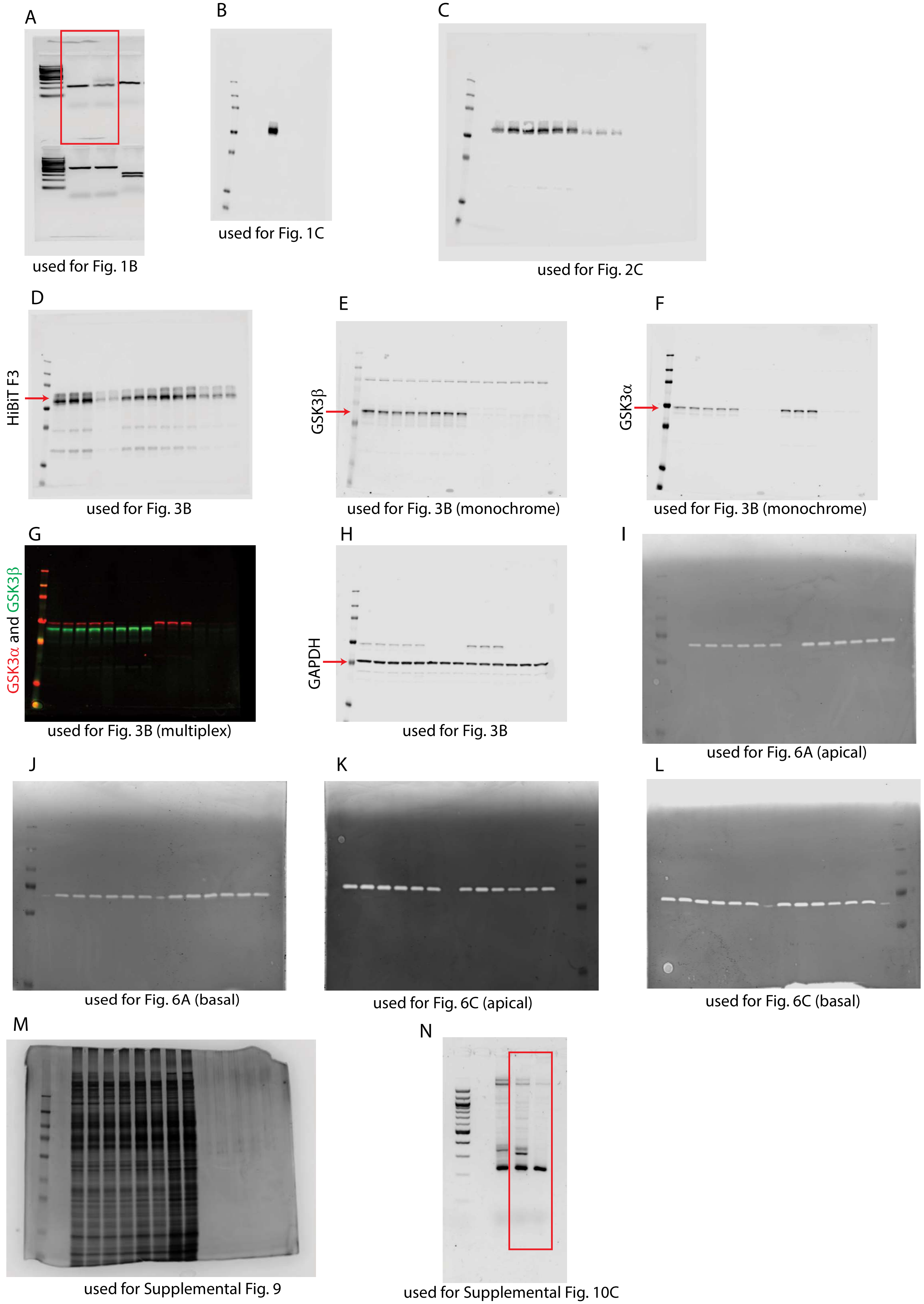

Supplement: Unedited blot and gel images [file jciinsight-9-178050-s221.pdf]
